# Orientational Chirality, Its Asymmetric Control, and Computational Study

**DOI:** 10.34133/research.0012

**Published:** 2022-12-19

**Authors:** Shengzhou Jin, Yu Wang, Yao Tang, Jia-Yin Wang, Ting Xu, Junyi Pan, Sai Zhang, Qiankai Yuan, Anis Ur Rahman, James D. McDonald, Guo-Qiang Wang, Shuhua Li, Guigen Li

**Affiliations:** ^1^ School of Chemistry and Chemical Engineering, Nanjing University, Nanjing, 210093, China.; ^2^ Department of Chemistry and Biochemistry, Texas Tech University, Lubbock, TX 79409-1061, USA.; ^3^ Continuous Flow Engineering Laboratory of National Petroleum and Chemical Industry, Changzhou University, Changzhou, Jiangsu 213164, China.

## Abstract

Orientational chirality was discovered and characterized by a C(sp)–C(sp^3^) axis-anchored chiral center and a remotely anchored blocker. X-ray structural analysis proved that orientatiomers are stabilized by through-space functional groups, making it possible for 1 *R*- or *S*-chiral center to exhibit 3 orientational isomers simply by rotating operations. A new model system was proposed, fundamentally different from the traditional Felkin–Ahn-type or Cram-type models. In these traditional models, chiral C(sp^3^) center and blocking C(sp^2^) carbons are connected adjacently, and there exist 6 energy barriers during rotating along the C(sp^2^)−C(sp^3^) axis. In comparison, the present orientational chirality model shows that a chiral C(sp)–C(sp^3^) carbon is remotely located from a blocking group. Thus, it is focused on the steric dialog between a chiral C(sp^3^) center and a remotely anchored functional group. There exist 3 energy barriers for either (*R*)– or (*S*)–C(sp)–C(sp^3^) stereogenicity in the new model. Chiral amide auxiliary was proven to be an excellent chiral auxiliary in controlling rotations of orientatiomers to give complete stereoselectivity. The asymmetric synthesis of individual orientatiomers was conducted via multistep synthesis by taking advantage of the Suzuki–Miyaura cross-coupling and Sonogashira coupling reactions. Density functional theory computational study presented optimized conformers and relative energies for individual orientatiomers. This discovery would be anticipated to result in a new stereochemistry topic and have a broad impact on chemical, biomedical, and material sciences in the future.

## Introduction

**Fig. 1.**
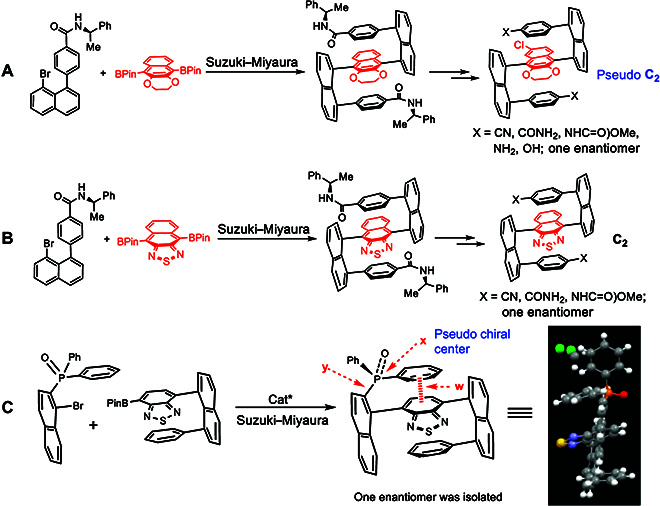
Asymmetric synthesis and catalysis of multilayer chirality. (A) Folding chirality with electron-efficient bridge; (B) Folding chirality with electron- deficient; (C) Folding chirality with pseudo chiral center.

Chirality phenomena has been existing in nature from the beginning of Earth’s lives in forms varying from microscopic living organisms (e.g., helical bacteria) to macroscopic objects (e.g., sea shells) [1–5]. Functional biomolecules, such as peptides/proteins, DNA/RNA, and carbohydrates, contain several types of chirality [6–8]. Modern pharmaceuticals heavily depend on chirality to govern their potency and selectivity so as to reduce dosages and unwanted side effects [8,9]. In modern material science, the control of chirality is necessitated so as to achieve challenging optoelectronic properties [10–13]. Asymmetric synthesis and catalysis have played important roles in these fields in the past several decades [14–41]. In general, chirality has been divided into the following categories: central [15], axial [17], spiral [14], sandwich (metallic [33,34] and organo [35,36]), and multilayer (rigid helical [11,37] and flexible folding [42,43]) chirality and inherent chirality [44]. In chirality research, *C*_2_ symmetry has been paid special attention concerning asymmetric control of designing chiral ligands and auxiliaries. In the meanwhile, multilayer *C*_2_ and pseudo *C*_2_ symmetry have become a new addition to the *C*_2_ symmetry category, which was represented by organo sandwich targets consisting of unique “S” and “Ƨ” patterns (Fig. 1) [45–47].

Very recently, we have reported the asymmetric assembly of multilayer chirality using chiral auxiliaries and catalysts (Fig. 1A to C). One single C–C bond formation can led to multilayer chirality in asymmetric catalysis (Fig. 1C) [42]. The resulting new chirality pattern is stabilized by aromatic/aromatic interaction (w in Fig. 1C) as shown in x-ray structures. The chiral multilayer framework consists of a pseudo-chiral center (x in Fig. 1C) and an orientational axis (y in Fig. 1C). The pseudo-chiral center on the phosphorus atom was directly connected to the naphthalenyl ring and 2 differentiated phenyl groups by parallel packing. The atropisomerism along the C–P axis is made jointly possible by Ar–Ar interaction and the parallel arrangement of the phenyl ring on the bottom of the structural framework. Concurrently, Sparr and Jørgensen laboratories [48,49] have successfully designed and achieved an asymmetric catalytic approach to stable atropisomers containing C(sp^2^)−cyclized C(sp^3^) σ bonds as axes. So far, the tetrahedron/plane-based rotamers had not become atropisomers until when the aforementioned laboratories were involved in this area, which is due to the low rotational energy barriers around the tetrahedron center plane axis.

During our ongoing research on multilayer folding chirality, we discovered a new type of chirality—orientational chirality in which a chiral tetrahedron center and a blocking group are anchored remotely through space. The orientational chiral isomers can be stabilized and asymmetrically synthesized by taking advantage of the structural analysis and design. To the best of our knowledge, noncyclized chiral C(sp^3^)-derived orientatiomers have not yet appeared in literature. Here, we would like to report our preliminary results of this discovery.

## Results

**Fig. 2.**
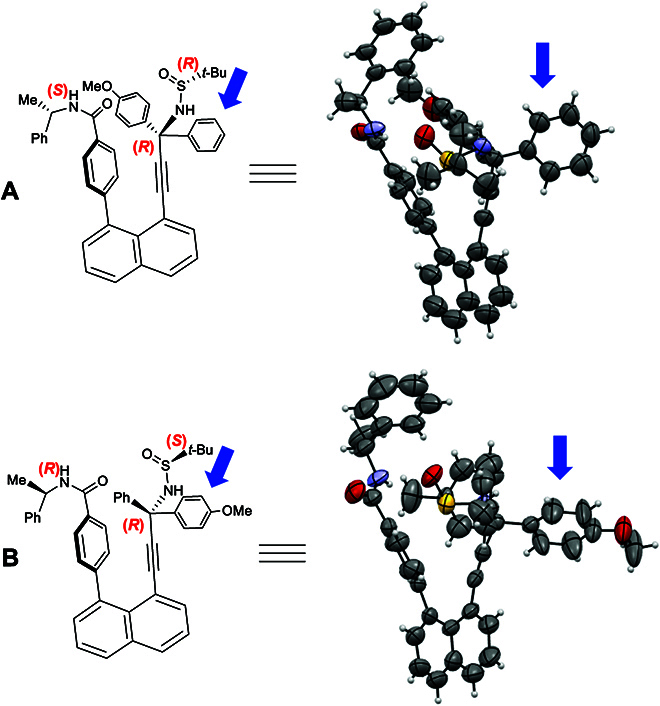
Differentiated orientational isomers confirmed by x-ray diffraction analysis. (A) Orientational isomer I with Ph group directed away; (B) Orientational isomer I with 4-MeOPh group directed away.

As described in Fig. 1, the chiral amide auxiliary has been utilized to control the asymmetric synthesis of multilayer folding chirality. In the resulting products, the naphthyl piers on bridge ends, forcing the chiral amide to rotate, leading different conformers as revealed by their x-ray structural analysis. We wondered whether a chiral tetrahedron moiety was introduced to replace the planar naphthyl pier, and we would like to know how the chiral amide subunit would be restricted. In the meanwhile, we would like to explore the orientational relationship between 2 chiral subunits of 2 levers/arms of the through-space framework (Fig. 2). After we obtained the initial products and their x-ray structures, we found that, while the chiral C(sp^3^) center anchored on the C(sp)–C(sp) lever on the right side was subjected to dialog with (*S*)- and (*R*)-chiral amides on the left lever (Fig. 2), 2 orientational isomers were observed for the same tetrahedron chiral C(sp^3^) center. The individual orientatiomers with 2 types of rotations have been unambiguously confirmed by their x-ray diffraction analysis. This observation indicates that other derivatives of these atropisomers would be stable enough to be synthesized asymmetrically. It should be pointed out that this atropisomerism is based on 4 independent flexible motifs attached to the C(sp^3^) carbon center, which made the present atropisomerism to be differentiated from previous systems containing cyclized rigid substituents centered on the C(sp^3^) carbon center [48,49].

### Asymmetric synthesis of orientatiomers

**Fig. 3.**
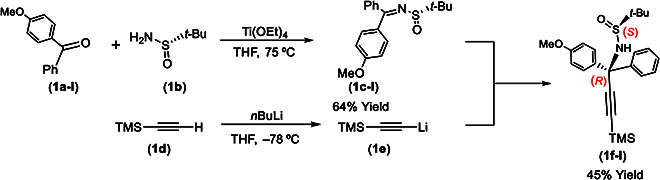
Asymmetric synthesis of (*S*)-*N*-(*R*)-precursors.

**Fig. 4.**
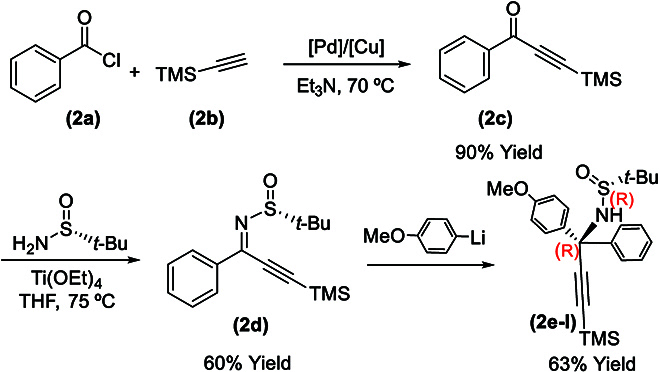
Asymmetric synthesis of (*R*)-*N*-((*R*)-precursors.

The asymmetric synthesis of orientatiomers is represented by the assembly of **4a** and **5a**, which were started from the formation of (*S*)-*N*-((*R*)-1-(4-methoxyphenyl)-1-phenyl-3-(trimethylsilyl)prop-2-yn-1-yl)-2-methylpropane-2-sulfinamide (**1f-I**) (Fig. 3). Dehydration reaction of (*S*)-2-methylpropane-2-sulfinamide (**1b**) with (4-methoxyphenyl)(phenyl)methanone (**1a-I**) was performed using Ti(OEt)_4_ in dry tetrahydrofuran (THF) at 75 °C for 24 h to give 91% yield [46,50]. The resulting *N*-sulfonyl ketimine (**1c-I**) was treated by ((trimethylsilyl)ethynyl)lithium, which was pre-generated from deprotonation of ethynyltrimethylsilane by *n*BuLi in THF at −78 °C, to afford (*S*)-*N*-((*R*)-1-(4-methoxyphenyl)-1-phenyl-3-(trimethylsilyl)prop-2-yn-1-yl)-2-methylpropane-2-sulfinamide (**1f-I**) in an overall yield of 32% from (4-methoxyphenyl)(phenyl)methanone.

(*R*)-*N*-((*R*)-1-(4-methoxyphenyl)-1-phenyl-3-(trimethylsilyl)prop-2-yn-1-yl)-2-methylpropane-2-sulfinamide (**2e-I**) was synthesized starting by forming 1-phenyl-3-(trimethylsilyl)prop-2-yn-1-one (**2c**) via the reaction between benzoyl chloride (**2a**) and ethynyltrimethylsilane (**2b**) in the presence of Pd/Cu cocatalysts (Fig. 4). Dehydration reaction of (*R*)-2-methylpropane-2-sulfinamide with 1-phenyl-3-(trimethylsilyl)prop-2-yn-1-one (**2c**) was conducted using Ti(OEt)_4_ in dry THF at 75 °C for 24 h to give (*R*,*Z*)-2-methyl-*N*-(1-phenyl-3-(trimethylsilyl)prop-2-yn-1-ylidene)propane-2-sulfinamide (**2d**) (54% yield), which was treated with (4-methoxyphenyl)lithium to afford (*R*)-*N*-((*R*)-1-(4-methoxyphenyl)-1-phenyl-3-(trimethylsilyl)prop-2-yn-1-yl)-2- methylpropane-2-sulfinamide (**2e-I**) in an overall yield of 34% from **2a**.

While the asymmetric synthesis of (*R*)-*N*-((*R*)-1-(4-methoxyphenyl)-1-phenyl-3-(trimethylsilyl)prop-2-yn-1-yl)-2-methylpropane-2-sulfinamide (**2e-I**) resulted in 1 diastereoisomer selectively, the generation of its (*R*)-*N*-((*R*)-counterpart (**1f-I**) was deemed challenging in regard to purification. The latter showed a diastereoselectivity of 2:1 at the key step of the electrophilic carbonyl addition reaction. The diastereo mixture was directly utilized for the following the Suzuki–Miyaura cross-coupling systems.

**Fig. 5.**
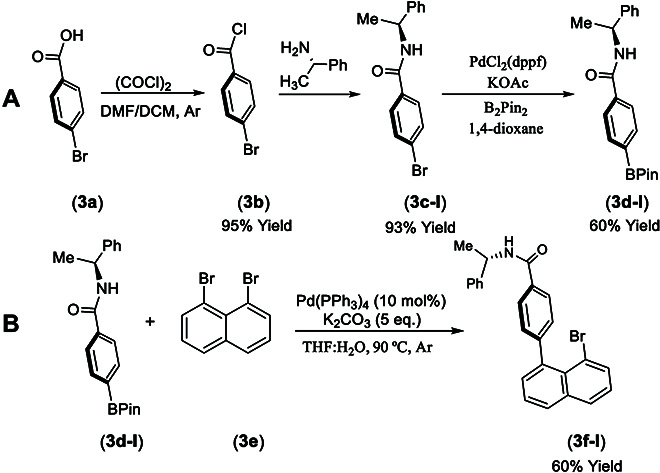
Synthesis of chiral amide-anchored precursor.

**Fig. 6.**
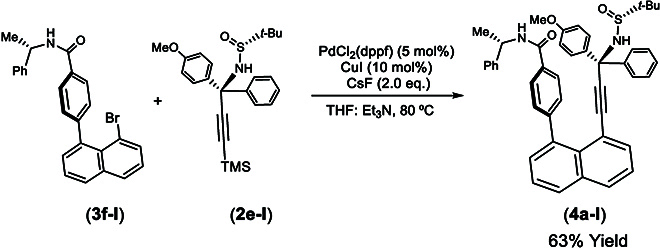
Assembly of orientational isomeric products.

4-Bromobenzoic acid (**3a**) was subjected to the treatment with oxalyl dichloride to give 4-bromobenzoyl chloride (**3b**), which was directly coupled with (*S*)-1-phenylethan-1-amine (Fig. 5A) [40]. The resulting (*S*)-4-bromo-*N*-(1-phenylethyl)benzamide (**3c-I**) was converted into (*S*)-*N*-(1-phenylethyl)-4-(4,4,5,5-tetramethyl-1,3,2-dioxaborolan-2-yl)benzamide (**3d-I**) by treating with B_2_Pin_2_ in the presence of PdCl_2_(dppf) as the catalyst and KOAc as an additive in 1,4-dioxane to give an overall yield of 64% from 4-bromobenzoic acid (**3a**).

(*S*)-4-(8-Bromonaphthalen-1-yl)-*N*-(1-phenylethyl)benzamide (**3f-I**) was obtained by reacting (*S*)-*N*-(1-phenylethyl)-4-(4,4,5,5-tetramethyl-1,3,2-dioxaborolan-2-yl)benzamide (**3d-I**) with 1,8-dibromonaphtalene (**3e**) under the Suzuki–Miyaura cross-coupling condition to give a yield of 61% (Fig. 5B) [51] The final assembly of the orientatiomeric target was conducted by coupling (*R*)-*N*-((*R*)-1-(4-methoxyphenyl)-1-phenyl-3-(trimethylsilyl)prop-2-yn-1-yl)-2-methylpropane-2-sulfinamide (**2e-I**) with (*S*)-4-(8-bromonaphthalen-1-yl)-*N*-(1-phenylethyl)benzamide (**3f-I**) in the presence of PdCl_2_(PPh_3_)_2_ (5 mol%) as the catalyst and CsF as the base (2.0 equiv) and CuI (10 mol%) as an additive in co-solvents of THF and NEt_3_ (4:1) at 80 °C for 12 h to give a yield of 50% (Fig. 6) [47,52,53]. As shown in Fig. 2, the absolute configurations of the isomeric products with 2 orientational directions have been unambiguously determined by x-ray crystallographic analysis.

**Fig. 7.**
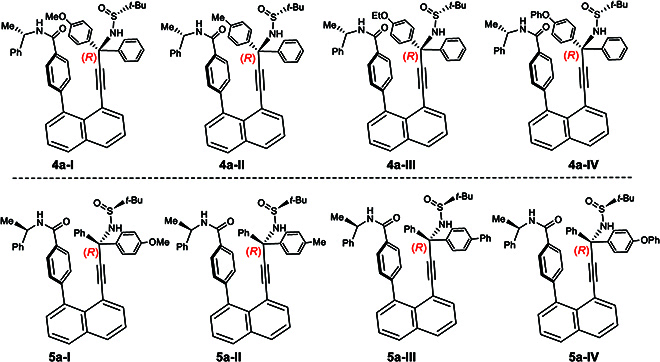
Orientational isomers controlled by chiral amide and *N*-sulfinyl groups.

Following the above multistep synthesis, various derivatives of **4a-I** and **5a-I** were obtained as 3 additional pairs of orientatiomers **4a-I** to **4a-IV** and **5a-I** to **5a-IV**, respectively (Fig. 7). Not only *p*-MeO substituent on benzene ring is anticipated to differentiate rotations along the C(chiral sp^3^)–C(sp) bond. Other 4 groups, such as Me-, EtO-, PhO-, and Ph-, can also result in orientatiomers. In the 4 derivatives on the first line in Fig. 7, the phenyl ring was pushed away toward the outside by (*S*)-amide auxiliary, whereas the para-substituted phenyl groups in the rest of the other 4 derivatives (on the second line in Fig. 7) are dictated toward outside by (*R*)-amide auxiliary. Similar to the first case, the final step gave a complete diastereoselectivity as well.

### Structural analysis and computation study

**Fig. 8.**
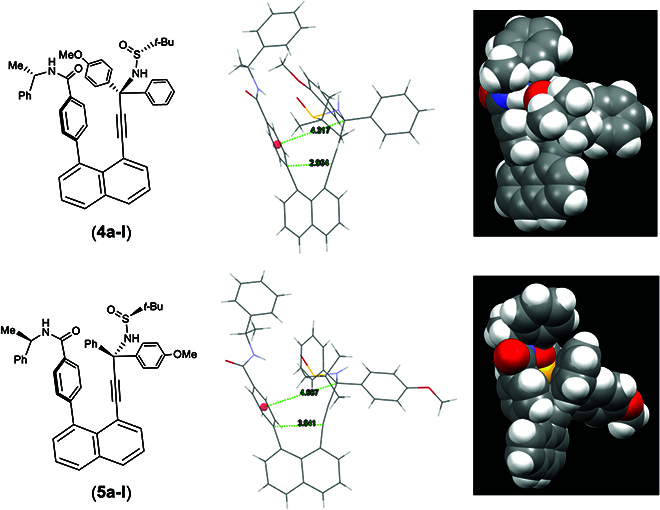
Distance measurements of orientational isomers.

As shown in x-ray diffraction analysis of single crystals of **4a-I** and **5a-I**, the (*S*)-amide auxiliary on the left side forces the chiral C(sp^3^) center away from the left aromatic center at a distance of 4.317 Å and the bottom C(sp^2^) on phenyl ring away from that of alkynyl moiety at a distances of 2.964 Å, respectively (Fig. 8A). However, the (*R*)-amide auxiliary on the left side resulted in these distances as 4.687 and 3.041 Å, respectively (Fig. 8B). The steric effects of the *p*-MeO- group on the phenyl ring of amide auxiliary, together with the orientation of the *t*-Bu group of 2-methylpropane-2-sulfinamide, are responsible for the differentiated distances. Interestingly, in both cases, H-C(sp^3^) of chiral amide auxiliary is directed toward the outside in the same direction as the O=S bond of 2-sulfinamide. The phenyl ring on the chiral amide auxiliary prefers to be on the same side of an aromatic ring of the chiral C(sp^3^) center, but away from the bulky *t*-Bu–S=O scaffold as anticipated. This is the key factor to control the orientation of the chiral C(sp^3^) center.

**Fig. 9.**
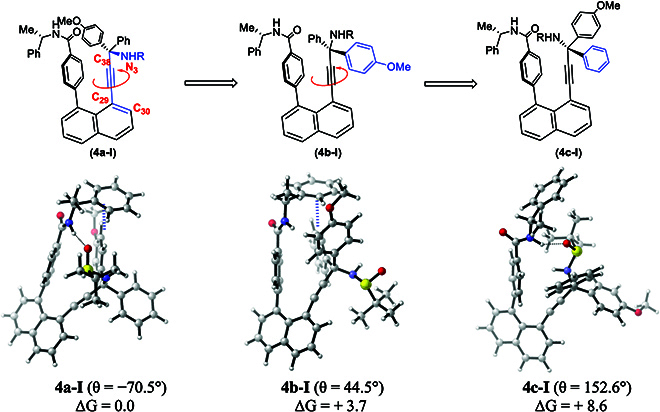
Optimized conformers and relative energies for orientatiomer 4a-I. R, (*R*)-tert-butylsulfinyl. Calculated at the M06-2X/cc-pVTZ (PCM, CH_3_CN)//M06-2X/6-31G (d,p) (PCM, CH_3_CN) level of theory (distances are in angstrom, and energies are given in kilocalorie per mole). H, white; C, gray; N, blue; O, red; S, yellow.

Computational investigation was conducted on orientational isomers **4a-I** and **5a-I** in regard to their relative energy (Fig. 9). Meanwhile, a rotational profile for **4a-I** by scanning the rotational dihedral angle θ was also obtained. All the density functional theory (DFT) calculations were performed at the M062x/cc-pVTZ//M062x/6-31G(d, p) level of theory [54,55] using the PCM solvation model [56]. Three-dimensional graphics on optimized structures were generated using CYLview (see the Supplementary Materials for computational details) [57,58].

The calculation results fully support and explain asymmetric synthetic induction, i.e., (*S*)-chiral amide auxiliary results in orientatiomer 1 in which the phenyl ring on the C(sp^3^) chiral center is directed away from the left lever of the predominant isomer **4a-I**, and (*R*)-chiral amide auxiliary results in orientatiomer **5a-I** in which para-MeO-phenyl ring on C(sp^3^) chiral center is directed away from the left lever. In both cases, energy is increased for forming isomers in which the *N*-sulfinyl group on the C(sp^3^) chiral center is directed away from the left lever of the products.

As shown in Fig. 9, isomer **4a-I** exists as the most stable isomer among 3 orientatiomers with the lowest energy setting at 0 kcal/mol for comparison convenience. The rotational operation around the C(sp)–C(sp^3^) bond leads to isomers **4b-I** and **4c-I**, with relative energies being 3.7 and 8.6 kcal/mol, respectively. This result indicates that this mostly accessible conformer is generated through the sterically favored pathway during the asymmetric controlling process. In isomer **4a-I**, there is intramolecular H bonding formation between amide hydrogen and sulfinyl oxygen with a length of 1.86 Å and edge-to-face *π*–*π* interactions [57] between amide Ph and 4-MeOPh groups (a distance of 2.48 Å). Interestingly, in isomer **4b-I**, there is a similar edge-to-face stacking (a distance of 2.55 Å), but no H bonding formation. However, in isomer **4c-I**, there is a similar H bonding formation (a length of 1.91 Å) but no *π* stacking. For orientatiomer **4a-I**, a conformational isomer without intramolecular *π*-*π* stacking between amide Ph and 4-MeOPh groups **4e-I** (see Supplementary Materials) could be located, and this species is predicted to be endothermic by 3.7 kcal/mol with respect to isomer **4a-I** if the rotational operation is enforced (see Fig. S1). On the basis of these computational analysis data, we believe that 2 types of noncovalent interactions contribute to the stability of the orientatiomer **4a-I**: H bonding interaction between amide hydrogen and sulfinyl oxygen and *π*-*π* interaction between amide Ph and 4-MeOPh groups. Similar noncovalent interactions also exist in orientatiomer **5a-I**; see Fig. S2 for details.

In addition, with isomer **4a-I** as the starting geometry, a relaxed scan on rotating the dihedral angle (C_30_–C_29_–C_38_–N_3_, θ = −70.5) around C(sp)–C(sp^3^), the isomerization of **4a-I** to **4b-I** (θ = 44.5°) requires to overcome a barrier of about 29 kcal/mol (θ = 34.5°), which is obviously higher to occur under room temperature (see Fig. S3). Our computational investigation would confirm that the asymmetric control is achieved with less-hindered interactions between the chiral amide auxiliary and the C(sp3) chiral center on the right anchor.

### Orientational chirality model

**Fig. 10.**
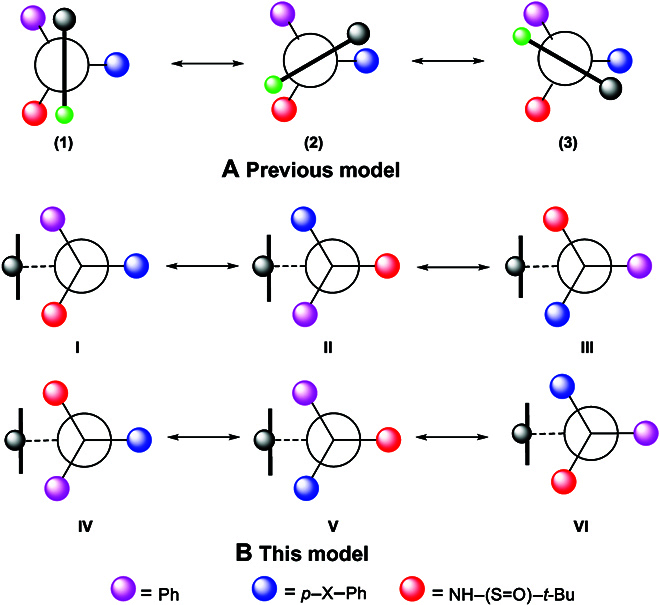
Models of previous (A) and new orientational chirality (B); There exist three pairs of enantiomers and six pairs of diastereomers.

Our previous orientational atropisomerism was based on the direct connections of C(sp^2^)−phosphorous tetrahedron center [38] or C(sp^2^)−C(sp^3^) [43,45] (Figs. 1C and 10A). X-ray diffraction analysis has proven that the former does not follow the Felkin–Ahn-type model, but the latter follows this model in which 1 of the 3 branches is arranged perpendicularly to the C(sp^2^)−planar ring. The reason for the former’s unusual behavior is due to the fact that the orientatiomer is stabilized by aromatic–aromatic interaction instead of the classic hyperconjugation or steric effects (Fig. 1C)

The previous multifold chirality shows a total of 6 energy barriers appearing during rotational operations caused by eclipsed conformational transition states (Fig. 10A) [43]. In contrast, in the present orientational chirality framework, there is no direct controlling force between the C(sp^3^) chiral stereogenicity and C(sp^2^) ring subunit (Fig. 10B). The remotely anchored aromatic ring is the only functional blocker that inhibits rotation along the C(sp)–C(sp^3^) axis. Therefore, the present orientational chirality is focused on the dialog relationship between the C(sp^3^) center and a remotely anchored steric blocker. Since only a single interaction (the heavy black line in the model) exists in each of the 3 atropisomers, there are 3 energy barriers instead of 6 in the previous 6-fold atropisomerism. It should also pointed out that the stereochemical measurements for the atropisomers, I to III, would not fit the classical *ee*/*er* or *de*/*dr* descriptions. Therefore, new descriptions would be suggested for measuring outcomes of asymmetric synthesis and catalysis for assembling these 3 chiral atropisomers, e.g., orientatiomeric selectivity of orientatiomeric excess (*oe*) and orientatiomeric ratios (*or*) would be utilized, respectively.

## Discussion

We have discovered the C(sp)–C(sp^3^) axis-based orientational chirality, showing that multiple orientations can be controlled by remotely anchored and through-space functional blockers. The rotamers along the C(sp)–C(sp^3^) axis were confirmed to become atropisomers. Chiral amide auxiliary was found to efficiently control rotations of orientatiomers in excellent stereoselectivity. The multistep synthesis resulted in differentiated chiral orientatiomers by conducting asymmetric nucleophilic addition, Suzuki–Miyaura cross-coupling, and Sonogashira coupling reactions in modest to good yields. The absolute configuration was confirmed by x-ray diffraction analysis of pure atropisomers. DFT computational study presented optimized conformers and relative energies for individual orientatiomers. A new model consisting of a remote blocking group was proposed, showing 3 main energy barriers during orientational rotation instead of 6 barriers in previous multifold systems. This discovery would be anticipated to result in a new stereochemistry area and to have a broad impact on chemical, biomedical, and material sciences in the future.

## Materials and Methods

Unless otherwise stated, all reactions were magnetically stirred and conducted in an oven-dried glassware in anhydrous solvents under Ar, applying standard Schlenk techniques. Solvents and liquid reagents, as well as solutions of solid or liquid reagents, were added via syringes, stainless steel cannulas, or polyethylene cannulas through rubber septa or through a weak Ar counterflow. Solvents were removed under reduced pressure at 40 to 65 °C using a Rotavapor. All given yields are isolated yields of chromatographically and nuclear magnetic resonance (NMR) spectroscopic materials. All commercially available chemicals were used as received without further purification.

^1^H and ^13^C NMR spectra were recorded in CDCl_3_ on 400- and 500-MHz instruments with TMS as the internal standard. For referencing of the ^1^H NMR spectra, the residual solvent signal (δ = 7.26 for CDCl_3_) was used. In the case of the ^13^C NMR spectra, the signal of solvent (δ = 7.16 for CDCl_3_) was used. Chemical shifts (δ) were reported in parts per million with respect to TMS. Data are represented as follows: chemical shift, multiplicity (s, singlet; d, doublet; t, triplet; m, multiplet), coupling constant (*J*, Hz), and integration. Optical rotations were measured with a Rudolph Research Analytical APIV/2W polarimeter at the indicated temperature with a sodium lamp. Measurements were performed in a 2-ml vessel with a concentration unit of g/100 ml in the corresponding solvents.

## Data Availability

All data are available in the manuscript or the Supplementary Materials.

## Supplementary Materials

Supplementary Materials Figs. S1 to S3. Sections S1 to S6.
