# Supplementary material for: Orientational Chirality, Its Asymmetric Control, and Computational Study
Source: Research (Wash D C). 2022 Dec 19;2022:0012. doi: 10.34133/research.0012 (PMC11407581; doi:10.34133/research.0012)
Supplement: Supplementary Materials — Figs. S1 to S3. Sections S1 to S6. [file research.0012.f1.pdf]

Supporting Information for

Orientational Chirality, its Asymmetric Control and Computational Study

Shengzhou Jin,[‡,§] Yu Wang,[‡,§] Yao Tang,[†,§] Jia-Yin Wang,[‡,#] Ting Xu,[‡,§] Junyi Pan,[‡] Sai Zhang,[†] Qiankai Yuan,[†] Anis Ur Rahman,[†] James D. McDonald,[†] Guo-Qiang Wang,[‡,]* Shuhua Li[‡,] and Guigen Li*,[†,‡]

[†] Department of Chemistry and Biochemistry, Texas Tech University, Lubbock, Texas 79409-1061, USA.

[‡] School of Chemistry and Chemical Engineering, Nanjing University, Nanjing, 210093, China.

[#] Continuous Flow Engineering Laboratory of National Petroleum and Chemical Industry, Changzhou University, Changzhou, Jiangsu 213164, China

*Email: guigen.li@ttu.edu

[§]These authors made equal contributions to this work

Table of Contents

1. General Information
2. Synthetic Procedures
3. NMR Spectra
4. X-ray and calculation Data
5. Calculation results
6. References

1. General Information

Unless otherwise stated, all reactions were magnetically stirred and conducted in oven-dried glassware in anhydrous solvents under Ar. Solvents and liquid reagents, as well as solutions of solid or liquid reagents were added directly or via syringes, or micropipette. Cooling baths were prepared in Dewar vessels filled with ice/water (0 °C). Heated oil baths were used for reactions requiring elevated temperatures. Solvents were removed under reduced pressure at 40-65 °C using a rotavapor. All given yields for small molecules are isolated yields of chromatographically and NMR spectroscopically materials.

All commercially available chemicals were used as received without further purification. Solvents as follows: MeOH, EtOH, toluene, EtOAc, DCM, dioxane, hexane, acetone and THF were used without further purification.

The ^{1}H and ^{13}C NMR spectra were recorded in $CDCl_3$ on 400 MHz and 100MHz instruments with TMS as internal standard. For referencing of the ^{1}H NMR spectra, the residual solvent signal (δ = 7.26 for $CDCl_3$) were used. In the case of the ^{13}C NMR spectra, the signal of solvents (δ = 77.06 ± 0.03 for $CDCl_3$) were used. Chemical shifts(δ) were reported in ppm with respect to TMS. Data are represented as follows: chemical shift, multiplicity (s = singlet, d = doublet, t = triplet, m = multiplet), coupling constant (*J*, Hz), and integration. MALDI-TOF analyses were carried out using an ABI/MDS SCIEX 4800 Mass Spectrometer with HABA matrix. UV-Vis spectra were collected on an Agilent 8453 UV-Visible Spectroscopy system. Fluorescence spectra were collected by Agilent Technologies Cary Eclipse Fluorescence Spectrophotometer G9800A and Eclipse ADL program.

X-ray data were collected on a Rigaku XtaLAB Synergy-*i* Kappa diffractometer equipped with a PhotonJet-*i* X-ray source operated at 50 W (50kV, 1 mA) to generate Cu Kα radiation (λ = 1.54178 Å) and a HyPix-6000HE HPC detector. Crystals were transferred from the vial and placed on a glass slide in polyisobutylene. A Zeiss Stemi 305 microscope was used to identify a suitable specimen for X-ray diffraction from a representative sample of the material. The crystal and a small amount of the oil were collected on a MĩTiGen cryoloop and transferred to the instrument where it was placed under a cold nitrogen stream (Oxford 700 series) maintained at 100K throughout the duration of the experiment. The sample was optically centered with the aid of a video camera to insure that no translations were observed as the crystal was rotated through all positions. A unit cell collection was then carried out. After it was determined that the unit cell was not present in the CCDC database a data collection strategy was calculated by *CrysAlis*^{Pro}[1]. The crystal was measured for size, morphology, and color. These values were reported in Tables.

2. Synthetic Procedures

2.1 General procedure for the synthesis of substrates **1c**

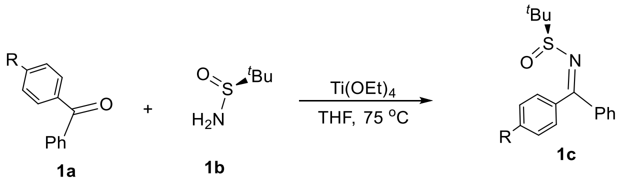

To a solution of (*S*)-(+)-2-methyl-2-propane sulfinamide (0.6 g, 5 mmol, 1 eq) and aryl ketones (6 mmol, 1.2 eq) in anhydrous THF (30 mL) at room temperature was added $Ti(OEt)_4$ (6 mL, 15 mmol, 3 eq). The mixture was heated at 75 °C for 24 h. After cooling to room temperature, the mixture was poured into 30 mL of brine under vigourous stirring. The resulting suspension was filtered through a pad of Celite and the solid was washed with EtOAc (3 x 20 mL). The filtrate was washed with brine (30 mL), dried (Na_2SO_4), and concentrated in vacuo. The residue was chromatographed on silica by eluting with PE/EA (10:1 to 5:1) to get the pure product.

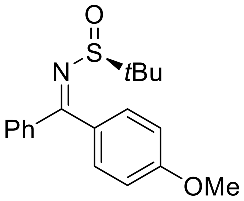

1c-I

(*S*,*Z*)-*N*-((4-methoxyphenyl)(phenyl)methylene)-2-methylpropane-2-sulfinamide (1c-I):
Purified by using a flash column chromatography (PE/EA = 10/1 to 5/1); isolated yield = 64%, 1.0093 g; yellow liquid. ^{1}H NMR (400 MHz, Chloroform-*d*) δ 7.64 (s, 2H), 7.51–7.31 (m, 5H), 6.90 (d, J = 8.4 Hz, 2H), 3.85 (s, 3H), 1.28 (s, 9H). ^{13}C NMR (101 MHz, Chloroform-*d*) δ 179.1, 131.6, 128.1, 113.6, 56.6, 55.4, 22.2.

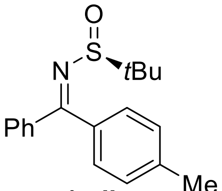

1c-II

(*S*,*Z*)-2-methyl-*N*-(phenyl(p-tolyl)methylene)propane-2-sulfinamide (1c-II):
Purified by using a flash column chromatography (PE/EA = 10/1 to 5/1); isolated yield = 60%, 0.8983 g; yellow liquid. ^{1}H NMR (400 MHz, Chloroform-*d*) δ 7.50–7.42 (m, 7H), 7.22 (d, J = 7.6 Hz, 2H), 2.41 (s, 3H), 1.29 (s, 9H). ^{13}C NMR (101 MHz, Chloroform-*d*) δ 179.5, 128.9, 128.1, 56.7, 22.3, 21.5.

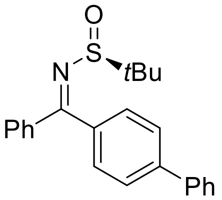

1c-III

(*S*,*Z*)-*N*-([1,1'-biphenyl]-4-yl(phenyl)methylene)-2-methylpropane-2-sulfinamide (1c-III):
Purified by using a flash column chromatography (PE/EA = 10/1 to 5/1); isolated yield = 60%, 0.9941 g; yellow liquid. ^{1}H NMR (400 MHz, Chloroform-*d*) δ 7.65–7.58 (m, 6H), 7.52–7.35 (m, 8H), 1.32 (s, 9H). ^{13}C NMR (101 MHz, Chloroform-*d*) δ 179.0, 140.0, 128.9, 128.2, 128.0, 127.2, 126.8, 56.9, 22.3.

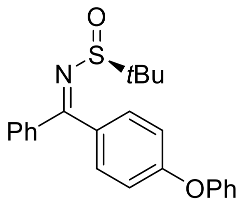

1c-IV

(*S*,*Z*)-2-methyl-*N*-((4-phenoxyphenyl)(phenyl)methylene)propane-2-sulfinamide (1c-IV):
Purified by using a flash column chromatography (PE/EA = 10/1 to 5/1); isolated yield = 57%, 1.0758 g; yellow liquid. ^{1}H NMR (400 MHz, Chloroform-*d*) *δ* 7.78–7.55 (m, 2H), 7.52-7.44 (m, 4H), 7.42–7.38 (m, 3H), 7.21–7.18 (m, 1H), 7.10 (d, *J* = 8.0 Hz, 2H), 6.99 (d, *J* = 8.4 Hz, 2H), 1.31 (s, 9H). ^{13}C NMR (101 MHz, Chloroform-*d*) *δ* 178.8, 171.7, 155.7, 131.7, 130.0, 128.3, 128.2, 124.5, 120.1, 117.3, 56.7, 22.3.

2.2 General procedure for the synthesis of substrates **1f**

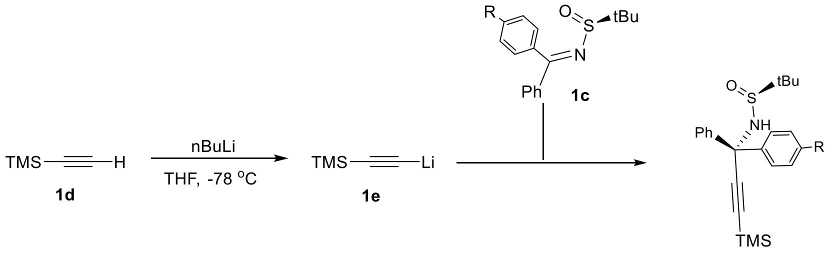

To a solution of terminal acetylene (0.3143g, 3.2 mmol) in dry THF (20 mL) at –78 °C was added *n*-butyllithium (2 mL, 1.6 M in hexanes). The resulting solution was stirred for 2 hours, a solution of **1** (3.2 mmol, 1 equiv) in dry THF (10 mL) was slowly added via syringe. Stirring was continued at –78 °C for 2 hours, and then heat the solution to room temperature for 14 hours. Subsequently, the saturated aqueous NH_4Cl was added to the solution, the organic phase was extracted with EA and dried over anhydrous $MgSO_4$. Chromatography (PE: EA= 10:1) afforded the diastereomerically pure propargyl **1f**.

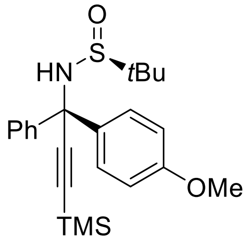

1f-I

(*S*)-*N*-((*R*)-1-(4-methoxyphenyl)-1-phenyl-3-(trimethylsilyl)prop-2-yn-1-yl)-2-methylpropane-2-sulfinamide (1f-I):
Purified by using a flash column chromatography (PE/EA = 10/1); isolated yield = 45%, 0.5956 g; yellow liquid. ^{1}H NMR (400 MHz, Chloroform-*d*) *δ* 7.59–7.52 (m, 4H), 7.33–7.23 (m, 3H), 6.88–6.84 (m, 2H), 3.97 (s, 1H), 3.79 (s, 3H), 1.23 (s, 9H), 0.25 (s, 9H). ^{13}C NMR (101 MHz, Chloroform-*d*) *δ* 159.2, 143.4, 135.5, 128.5, 128.2, 127.7, 127.5, 113.7, 107.1, 93.5, 63.9, 56.7, 55.2, 22.8, -0.2.

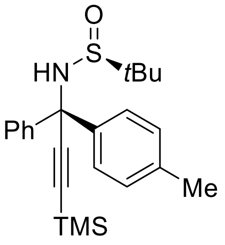

1f-II

(*S*)-2-methyl-*N*-((*R*)-1-phenyl-1-(p-tolyl)-3-(trimethylsilyl)prop-2-yn-1-yl)propane-2-sulfinamide (1f-II):

Purified by using a flash column chromatography (PE/EA = 10/1); isolated yield = 50%, 0.6362 g; yellow liquid. ^{1}H NMR (400 MHz, Chloroform-*d*) δ 7.64 (d, *J* = 7.6 Hz, 1H), 7.58 (d, *J* = 7.2 Hz, 1H), 7.51 (d, *J* = 8.0 Hz, 1H), 7.45 (d, *J* = 8.0 Hz, 1H), 7.36–7.23 (m, 3H), 7.12 (dd, *J* = 14.4, 8.0 Hz, 2H), 3.97 (s, 1H), 2.32 (d, *J* = 3.6 Hz, 3H), 1.23 (d, *J* = 2.0 Hz, 9H), 0.25 (s, 9H). 13C NMR (101 MHz, Chloroform-*d*) δ 143.7, 143.2, 140.6, 140.1, 137.6, 129.1, 128.9, 128.3, 128.2, 127.7, 127.5, 127.5, 127.0, 107.1, 107.0, 93.63, 93.58, 64.0, 56.7, 22.7, 223, 21.0, -0.2.

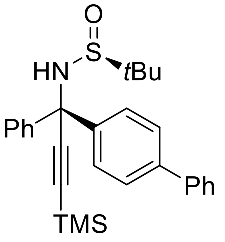

1f-III

(*S*)-*N*-((*R*)-1-([1,1'-biphenyl]-4-yl)-1-phenyl-3-(trimethylsilyl)prop-2-yn-1-yl)-2-methylpropane-2-sulfinamide (1f-III):

Purified by using a flash column chromatography (PE/EA = 10/1); isolated yield = 42%, 0.6178 g; yellow liquid. ^{1}H NMR (400 MHz, Chloroform-*d*) δ 7.73–7.67 (m, 4H), 7.61–7.56 (m, 4H), 7.45–7.43 (m, 2H), 7.4–27.36 (m, 3H), 7.34–7.30 (m, 1H), 4.06 (s, 1H), 1.29 (s, 9H), 0.30 (s, 9H). ^{13}C NMR (101 MHz, Chloroform-*d*) δ 143.6, 142.1, 140.7, 140.4, 128.8, 128.5, 128.1, 127.9, 127.5, 127.1, 127.1, 126.9, 106.8, 94.1, 64.1, 56.9, 22.8, -0.2.

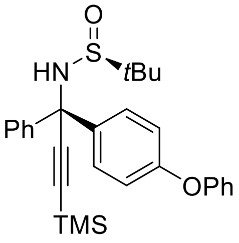

1f-IV

(*S*)-2-methyl-*N*-((*R*)-1-(4-phenoxyphenyl)-1-phenyl-3-(trimethylsilyl)prop-2-yn-1-yl)propane-2-sulfinamide (1f-IV):

Purified by using a flash column chromatography (PE/EA = 4/1); isolated yield = 39%, 0.5936 g; yellow liquid. ^{1}H NMR (400 MHz, Chloroform-*d*) δ 7.62 (d, *J* = 8.0 Hz, 2H), 7.54 (d, *J* = 6.8 Hz, 2H), 7.38–7.30 (m, 5H), 7.15–7.10 (m, 1H), 7.00 (d, *J* = 8.3 Hz, 2H), 6.93 (d, *J* = 7.0 Hz, 2H), 4.25 (br, 1H), 1.21

(s, 9H), 0.23 (s, 9H). ^{13}C NMR (101 MHz, Chloroform-*d*) δ 157.8, 156.1, 138.8, 133.1, 129.7, 128.6, 126.8, 123.8, 119.5, 118.0, 101.9, 96.1, 60.3, 31.0, 25.0, -0.5.

2.3 General procedure for the synthesis of substrates **2d**

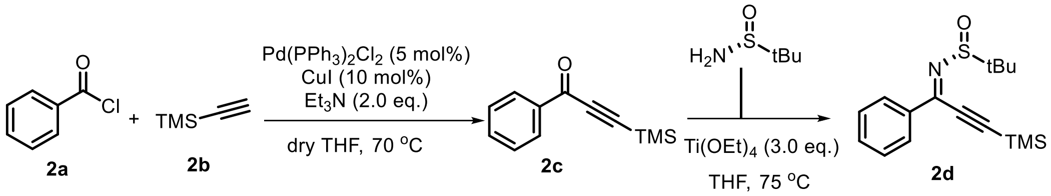

To a solution of $Pd(PPh_3)_2Cl_2$ (350.9 mg, 5 mol%), CuI (190.5 mg, 10 mol%), and Et_3N (2.8 mL, 20 mmol, 2.0 equiv.) in dry THF was added under Ar atmosphere. The benzoyl chloride (1.4 g, 10 mmol, 1.0 equiv.) and the terminal acetylene (1.2 g, 12 mmol, 1.2 equiv.) were then added to the mixture, which was heated under Ar atmosphere at 70 °C in oil bath for 18 h. After completed, the reaction mixture was quenched with water and extracted with ethyl acetate (3×10 mL). The combined organic layer was washed with brine, dried over $MgSO_4$, and concentrated in vacuo. The residue was purified by column chromatography on silica gel (petrol ether) to afford the desired product (90% yield).

To a solution of (*R*)-(+)-2-methyl-2-propane sulfinamide (1.08 g, 9 mmol, 1.0 eq.) and **2** (1.8 g, 9 mmol, 1.0 equiv.) in anhydrous THF (25 mL) at room temperature was added $Ti(OEt)_4$ (10.8 mL, 27 mmol, 3.0 equiv.). The mixture was heated at 75°C for 24 h. After cooling to room temperature, the mixture was poured into 5 mL of brine under vigourous stirring. The resulting suspension was filtered through a pad of Celite and the solid was washed with EtOAc (3×10 mL). The filtrate was dried over $MgSO_4$, and concentrated in vacuo. The residue was purified by column chromatography on silica gel (petrol ether/EtOAc = 10:1) to afford the desired product **2d** (60% yield).

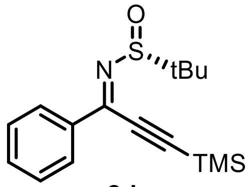

2d

(*R*,*Z*)-2-methyl-*N*-(1-phenyl-3-(trimethylsilyl)prop-2-yn-1-ylidene)propane-2-sulfinamide(2d): Purified by using a flash column chromatography (PE/EA = 10/1); isolated yield = 60%, 1.6497 g; yellow liquid. ^{1}H NMR (400 MHz, Chloroform-*d*) δ 8.14 (d, J = 8.0 Hz, 2H), 7.55–7.52 (m, 1H), 7.46–7.43 (m, 2H), 1.31 (s, 9H), 0.33 (s, 9H). ^{13}C NMR (101 MHz, Chloroform-*d*) δ 158.7, 135.6, 132.7, 128.7, 128.5, 112.3, 96.3, 58.1, 22.4, -0.7.

2.4 General procedure for the synthesis of substrates **2e**

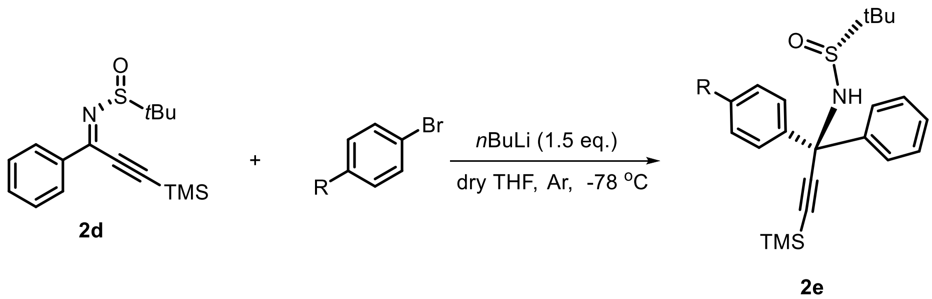

2e

To a solution of aryl bromide (8.1 mmol, 1.5 equiv.) in dry THF (30 mL) at –78 °C was added *n*-

butyllithium (3.4 mL, 1.6 M in hexanes) under Ar atmosphere. The resulting solution was stirred for 2 hours, a solution of **3** (1.62 g, 5.4 mmol, 1.0 equiv.) in dry THF (10 mL) was then slowly added via syringe. Stirring was continued at –78 °C for 10 hours. Subsequently, the reaction mixture was quenched with saturated NH_4Cl aqueous solution and extracted with ethyl acetate (3×10 mL). The organic layer was dried over $MgSO_4$, and concentrated in vacuo. The residue was purified by column chromatography on silica gel (PE/EA = 10:1 to 5:1) to afford the desired product **2e**.

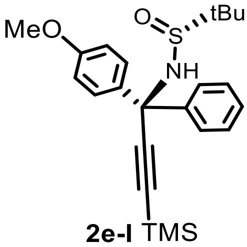

2e-I

(*R*)-*N*-((*R*)-1-(4-methoxyphenyl)-1-phenyl-3-(trimethylsilyl)prop-2-yn-1-yl)-2-methylpropane-2-sulfinamide (2e-I): Purified by using a flash column chromatography (PE/EA = 10/1); isolated yield = 63%, 1.4071 g; yellow liquid. ^{1}H NMR (400 MHz, Chloroform-*d*) δ 7.61–7.55 (m, 4H), 7.36–7.28 (m, 3H), 6.89 (d, J = 8.4 Hz, 2H), 3.99 (s, 1H), 3.81 (s, 3H), 1.26 (s, 9H), 0.27 (s, 9H). ^{13}C NMR (101 MHz, Chloroform-*d*) δ 159.2, 143.5, 135.5, 128.5, 128.2, 127.8, 127.5, 113.7, 107.1, 93.5, 63.9, 56.7, 55.3, 22.8, -0.2.

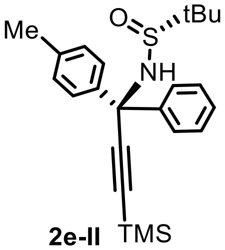

2e-II

(*R*)-2-methyl-*N*-((*R*)-1-phenyl-1-(p-tolyl)-3-(trimethylsilyl)prop-2-yn-1-yl)propane-2-sulfinamide (2e-II). Purified by using a flash column chromatography (PE/EA = 5/1); isolated yield = 72%, 1.5460 g; yellow liquid. ^{1}H NMR (400 MHz, Chloroform-d) δ 7.64 (d, J = 7.2 Hz, 1H), 7.58 (d, J = 7.6 Hz, 1H), 7.51 (d, J = 8.0 Hz, 1H), 7.45 (d, J = 8.0 Hz, 1H), 7.35–7.24 (m, 3H), 7.12 (dd, J = 16.0, 8.0 Hz, 2H), 3.98 (s, 1H), 2.32 (d, J = 3.6 Hz, 3H), 1.24 (s, 9H), 0.25 (s, 9H). ^{13}C NMR (101 MHz, Chloroform-d) δ 143.7, 143.2, 140.6, 140.1, 137.6, 129.1, 128.9, 128.3, 128.2, 127.7, 127.5, 127.5, 127.0, 107.1, 93.6, 64.0, 56.8, 22.7, 20.9, -0.2.

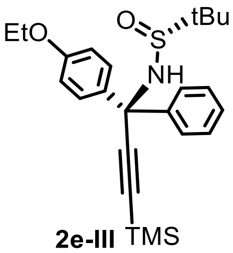

2e-III

(*R*)-*N*-((*R*)-1-(4-ethoxyphenyl)-1-phenyl-3-(trimethylsilyl)prop-2-yn-1-yl)-2-methylpropane-2-sulfinamide (2e-III): Purified by using a flash column chromatography (PE/EA = 10/1); isolated yield = 47%, 1.0852 g; yellow liquid. ^{1}H NMR (400 MHz, Chloroform-*d*) δ 7.62–7.59 (m, 2H), 7.52 (dd, J =

19.2, 8.2 Hz, 4H), 7.32 (d, J = 7.6 Hz, 2H), 6.87 (d, J = 8.6 Hz, 2H), 4.03 (t, J = 7.0 Hz, 2H), 3.98 (s, 1H), 1.41 (d, J = 7.4 Hz, 3H), 1.26 (s, 9H), 0.27 (s, 9H). ^{13}C NMR (101 MHz, Chloroform-*d*) δ 158.5, 143.5, 135.3, 128.5, 128.2, 127.7, 127.5, 114.2, 107.2, 93.5, 63.4, 56.6, 22.8, 14.7, -0.2.

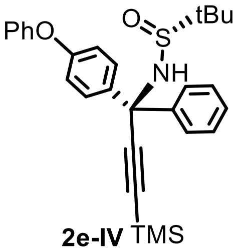

2e-IV

(*R*)-2-methyl-*N*-((*R*)-1-(4-phenoxyphenyl)-1-phenyl-3-(trimethylsilyl)prop-2-yn-1-yl)propane-2-sulfinamide (2e-IV). Purified by using a flash column chromatography (PE/EA = 5/1); isolated yield = 65%, 1.6697 g; yellow liquid. ^{1}H NMR (400 MHz, Chloroform-d) δ 7.61–7.58 (m, 4H), 7.36–7.25 (m, 5H), 7.11 (t, J = 7.4 Hz, 1H), 7.02 (d, J = 6.4 Hz, 2H), 6.95 (dd, J = 8.6, 2.2 Hz, 2H), 4.07 (s, 1H), 1.24 (s, 9H), 0.25 (s, 9H). ^{13}C NMR (101 MHz, Chloroform-d) δ 157.0, 156.4, 143.0, 137.8, 129.6, 128.5, 128.1, 127.7, 127.3, 123.5, 119.3, 117.9, 106.7, 93.7, 63.7, 56.7, 22.6, -0.4.

2.5 General procedure for the synthesis of substrates **3c**

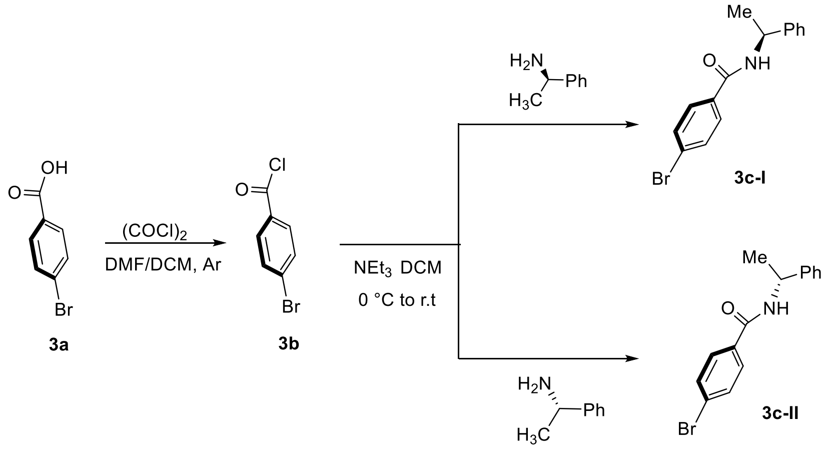

A 50 mL round bottom flask was charged with the aryl carboxylic acid (10 mmol) and dichloromethane (40 mL) under an atmosphere of nitrogen. The solution was cooled to 0 ºC in an ice bath and oxalyl chloride (12 mmol) was added to the reaction mixture followed by three drop of DMF. The reaction was stirred at 0 ºC for one hour and then warmed to room temperature and stirred for two hours. The solution was then concentrated under reduced pressure to get the p-bromobenzoyl chloride in 95% yield (2.1068 g, 9.5 mmol).

To a cooled (0 °C) solution of **3b** (9.5 mmol) in dry DCM (30 mL) was added Et_3N (28.5 mmol, 3 equiv) and (*S*)-1-phenylethan-1-amine or (*R*)-1-phenylethan-1-amine (9.5 mmol, 1 equiv). The reaction mixture was warmed to room temperature and stirred for 2 h. The mixture was washed with aq. HCl (1 M, 15 mL), saturated aq. $NaHCO_3$ (15 mL) successively. The combined organic layers were dried by $MgSO_4$ and concentrated the resulting mixture under reduced pressure. Finally, the **3c-I** or **3c-II** was obtained by recrystallizing the crude product.

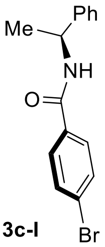

3c-I

(*S*)-4-bromo-*N*-(1-phenylethyl)benzamide (3c-I). Employing the general procedure and purified by recrystallization as white solid (3.2251 g, 93% yield). ^{1}H NMR (400 MHz, Chloroform-d) δ 7.62 (d, J = 8.4 Hz, 2H), 7.53 (d, J = 8.4 Hz, 2H), 7.38–7.33 (m, 4H), 7.30–7.26 (m, 1H), 6.39 (d, J = 8.4 Hz, 1H), 5.34–5.27 (m, 1H), 1.59 (d, J = 6.8 Hz, 3H). ^{13}C NMR (101 MHz, Chloroform-d) δ 165.6, 142.9, 133.4, 131.7, 128.8, 128.6, 127.5, 126.2, 126.1, 49.4, 21.6.

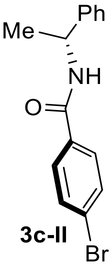

3c-II

(*R*)-4-bromo-*N*-(1-phenylethyl)benzamide (3c-II). Employing the general procedure and purified by recrystallization as white solid (3.2944 g, 95% yield). ^{1}H NMR (400 MHz, Chloroform-*d*) δ 7.63 (d, J = 8.4 Hz, 2H), 7.51 (d, J = 8.5 Hz, 2H), 7.37–7.34 (m, 4H), 7.31–7.28 (m, 1H), 6.69 (d, J = 6.7 Hz, 1H), 5.33–5.26 (m, 1H), 1.59 (d, J = 6.9 Hz, 3H). ^{13}C NMR (101 MHz, Chloroform-*d*) δ 165.8, 143.1, 133.5, 131.7, 128.7, 128.7, 127.5, 126.2, 126.1, 49.4, 21.7.

2.6 General procedure for the synthesis of substrates **3d**

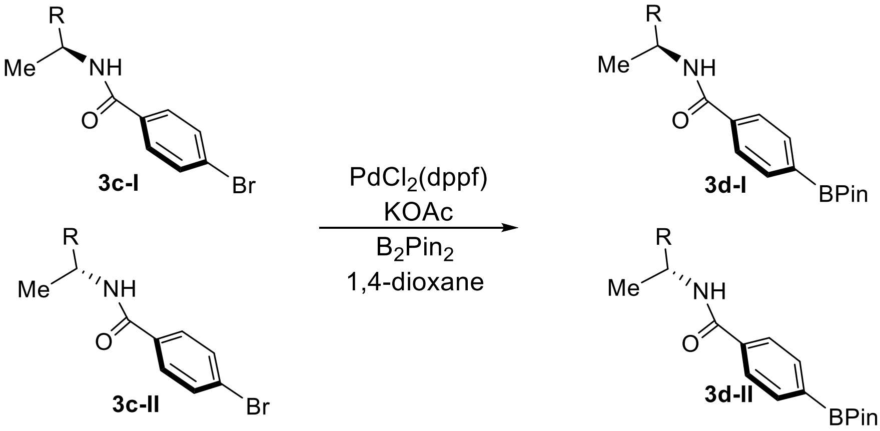

The mixture of **3c-I** (or **3c-II**) (2.7377 g, 9 mmol), bis(pinacolato)diboron (3.0473 g, 12 mmol), potassium acetate (1.7.665 g, 18 mmol), Pd(dppf)Cl_2 (0.7349 g, 0.9 mmol) was protected with Ar, and then the 80 mL dry 1,4-dioxane was added. The mixture was heated at 110 °C (oil bath) for 20 h. After cooling down the mixture was filtered through a pad of celite. Removal of solvent under reduced pressure afforded a residue which is purified by chromatography on silica gel (PE: EA=4:1) to afford the white product **3d-I** (or **3d-II**).

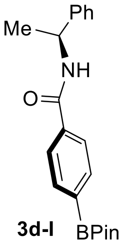

3d-I

(*S*)-*N*-(1-phenylethyl)-4-(4,4,5,5-tetramethyl-1,3,2-dioxaborolan-2-yl)benzamide (3d-I). Purified by using a flash column chromatography (PE/EA = 4/1); isolated yield = 60%, 1.8967 g; white solid. ^{1}H NMR (400 MHz, Chloroform-d) δ 7.85 (d, J = 8.4 Hz, 2H), 7.74 (d, J = 8.4 Hz, 2H), 7.40–7.33 (m, 4H), 7.29–7.25 (m, 1H), 6.42 (d, J = 7.6 Hz, 1H), 5.34 (m, 1H), 1.60 (d, J = 6.8 Hz, 3H), 1.35 (s, 12H). ^{13}C NMR (101 MHz, Chloroform-d) δ 166.5, 143.1, 136.80, 134.9, 128.7, 127.4, 126.3, 126.0, 84.1, 49.2, 24.8, 21.7.

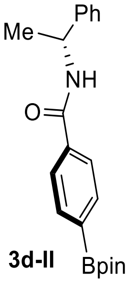

3d-II

(*R*)-*N*-(1-phenylethyl)-4-(4,4,5,5-tetramethyl-1,3,2-dioxaborolan-2-yl)benzamide (3d-II). Purified by using a flash column chromatography (PE/EA = 4/1); isolated yield = 71%, 2.2128 g; white solid.^{1}H NMR (400 MHz, Chloroform-*d*) δ 7.86 (d, J = 8.2 Hz, 2H), 7.78 (d, J = 8.1 Hz, 2H), 7.41–7.34 (m, 4H), 7.30–7.26 (m, 1H), 6.64 (s, 1H), 5.37–5.30 (m, 1H), 1.60 (d, J = 6.9 Hz, 3H), 1.37 (s, 12H). ^{13}C NMR (101 MHz, Chloroform-*d*) δ 166.6, 143.2, 136.9, 134.9, 128.7, 127.4, 127.4, 126.3, 126.1, 84.1, 49.3, 24.9, 21.7.

2.7 General procedure for the synthesis of substrates **3f**

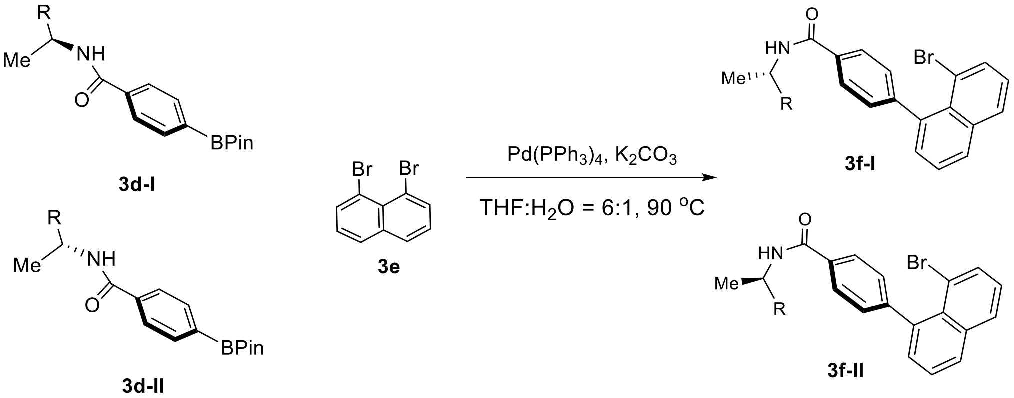

Place 1,8-dibromonaphtalene (1.4298 g, 5 mmol), **7** (or **8**) (1.7563 g, 5 mmol), K_2CO_3 (2.0730 g, 15 mmol) and $Pd(PPh_3)_4$ (0.5778, 0.5 mmol) successively to a flask under an argon atmosphere. Add THF (30 mL) and water (5 mL). Heat the mixture in an oil bath at 90 °C. Stir the mixture for 16 h. Cooled the mixture to room temperature and the liquid was separated with EA and water. The organic phase was taken and purified after concentration on silica gel (PE : EA = 5 : 1) to get the desired product **3f-I** (or **3f-II**).

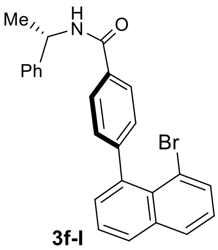

3f-I

(*S*)-4-(8-bromonaphthalen-1-yl)-*N*-(1-phenylethyl)benzamide (3f-I). Purified by using a flash column chromatography (PE/EA = 5/1); isolated yield = 60%, 1.2910 g; yellow solid. ^{1}H NMR (400 MHz, Chloroform-d) δ 7.89 (d, J = 8.4 Hz, 2H), 7.82–7.76 (m, 3H), 7.49 (m, 1H), 7.44 (d, J = 7.2 Hz, 2H), 7.40–7.35 (m, 5H), 7.32–7.27 (m, 2H), 6.47 (d, J = 7.6 Hz, 1H), 5.43–5.36 (m, 1H), 1.64 (d, J = 7.2 Hz, 3H). ^{13}C NMR (101 MHz, Chloroform-d) δ 166.5, 146.4, 143.2, 139.3, 136.1, 133.8, 133.0, 131.1, 130.33, 130.32, 129.4, 129.3, 128.9, 128.8, 127.5, 126.3, 126.2, 126.1, 126.0, 125.3, 119.9, 49.3, 21.7.

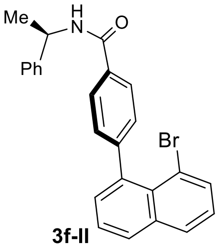

3f-II

(*R*)-4-(8-bromonaphthalen-1-yl)-*N*-(1-phenylethyl)benzamide (3f-II). Purified by using a flash column chromatography (PE/EA = 5/1); isolated yield = 61%, 1.3125 g; yellow solid.^{1}H NMR (400 MHz, Chloroform-*d*) δ 7.92 (d, J = 8.1 Hz, 2H), 7.84–7.80 (m, 3H), 7.53 (t, J = 7.6 Hz, 1H), 7.47 (d, J = 7.5 Hz, 2H), 7.43–7.39 (m, 5H), 7.35–7.30 (m, 2H), 6.49 (d, J = 7.3 Hz, 1H), 5.46–5.39 (m, 1H), 1.68 (d, J = 7.1 Hz, 3H). ^{13}C NMR (101 MHz, Chloroform-*d*) δ 166.5, 146.5, 143.2, 139.3, 136.1, 133.8, 133.1, 131.1, 130.4, 129.4, 129.3, 129.0, 128.8, 127.5, 126.4, 126.3, 126.1, 126.0, 125.3, 120.0, 49.3, 21.8.

2.8 General procedure for the synthesis of substrates **4a**

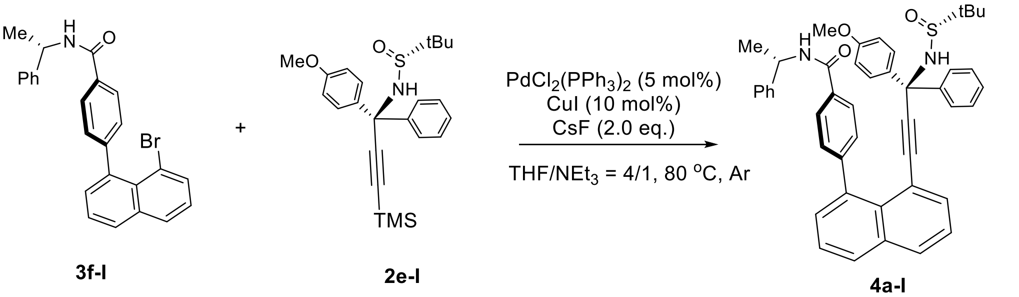

entry	variation of the reaction conditions	yield (%)[b]
1	none	63
2	Cs_2CO_3 instead of CsF	58
3	Na_2CO_3 instead of CsF	<10%
4	DMF instead of THF/NEt_3	30
5	THF instead of THF/NEt_3	52
6	toluene instead of THF/NEt_3	35
7	$Pd(OAc)_2$ instead of $PdCl_2(PPh_3)_2$	30

8	$PdCl_2$(dppf) instead of $PdCl_2(PPh_3)_2$	40
9	$Pd_2(dba)_3$ instead of $PdCl_2(PPh_3)_2$	27
10	$Pd(PPh_3)_4$ instead of $PdCl_2(PPh_3)_2$	trace
11	at 100 °C	60
12	$PdCl_2(PPh_3)_2$ (10 mol%)	62

[a]Reaction conditions: **3f-I** (0.1 mmol), **2e-I** (0.1 mmol), $PdCl_2(PPh_3)_2$ (5 mol%), CuI (10 mol%), CsF (2.0 equiv) solvent 2 mL under Ar conditions. [b]Isolated yield based on **4a-I**.

The mixture of **9** (0.1 mmol, 1.0 equiv.), $PdCl_2(PPh_3)_2$ (3.5 mg, 5 mol%), CuI (1.9 mg, 10 mol%), and CsF (30.4 mg, 0.2 mmol, 2.0 equiv.) in THF/NEt_3 (2 mL) was added the mixture of **3** (41.4 mg, 0.1 mmol, 1 equiv.) under Ar atmosphere. Then the mixture was heated in an oil bath at 80 °C and stirred for 12 h. After cooling to the temperature, to the mixture was added water (10 mL) and extracted with ethyl acetate (3×10 mL). The organic layer was dried over $MgSO_4$, and concentrated in vacuo. The residue was purified by column chromatography on silica gel (PE/EA = 5 : 1) to afford the desired product **4a**.

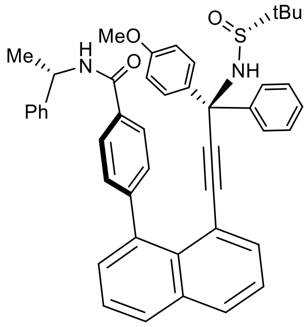

4a-I

4-(8-((*R*)-3-(((*R*)-tert-butylsulfinyl)amino)-3-(4-methoxyphenyl)-3-phenylprop-1-yn-1-yl)naphthalen-1-yl)-*N*-((*S*)-1-phenylethyl)benzamide (4a-I). Purified by using a flash column chromatography (PE/EA = 5/1); isolated yield = 63%, 43.5 mg; yellow liquid. ^{1}H NMR (400 MHz, Chloroform-d): δ 7.97 (dd, J = 7.2, 1.2 Hz, 1H), 7.93 (dd, J = 8.2, 1.4 Hz, 1H), 7.86 (dd, J = 8.2, 1.4 Hz, 1H), 7.52–7.44 (m, 5H), 7.39–7.36 (m, 3H), 7.32–7.30 (m, 3H), 7.28–7.25 (m, 4H), 7.22–7.16 (m, 4H), 7.09–7.04 (m, 3H), 6.49 (d, J = 9.2 Hz, 2H), 5.30–5.23 (m, 1H), 3.70 (s, 4H), 1.58 (d, J = 6.8 Hz, 3H), 1.22 (s, 9H). ^{13}C NMR (101 MHz, $CDCl_3$): δ 167.3, 159.0, 145.0, 143.9, 143.8, 139.6, 135.8, 135.4, 134.6, 133.9, 130.8, 130.1, 123.0, 129.2, 128.8, 128.5, 128.3, 128.0, 127.7, 127.2, 126.8, 126.5, 126.2, 125.4, 125.3, 119.8, 113.4, 99.2, 89.0, 64.4, 56.7, 55.4, 49.2, 22.8, 21.9. HRMS (ESI): *m/z* calcd. For $C_{45}H_{43}N_2O_3S$ $[M + H]^+$ 691.2989, found 691.2971, $[\alpha]_D^{25}$ = –41.0 (c = 0.60, $CHCl_3$).

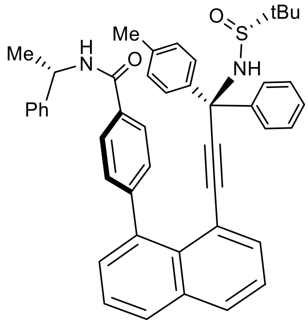

4a-II

4-(8-((*R*)-3-(((*R*)-tert-butylsulfinyl)amino)-3-phenyl-3-(*p*-tolyl)prop-1-yn-1-yl)naphthalen-1-yl)-*N*-((*S*)-1-phenylethyl)benzamide (4a-II). Purified by using a flash column chromatography (PE/EA = 5/1);

isolated yield = 55%, 37.1 mg; yellow liquid. ^{1}H NMR (400 MHz, Chloroform-d) δ 7.99 (dd, J = 7.2, 1.6 Hz, 1H), 7.92 (dd, J = 8.2, 1.4 Hz, 1H), 7.86 (dd, J = 8.2, 1.4 Hz, 1H), 7.52–7.46 (m, 3H), 7.41 (dd, J = 8.0, 1.6 Hz, 1H), 7.38–7.31 (m, 7H), 7.30–7.27 (m, 2H), 7.23–7.19 (m, 3H), 7.08 (d, J = 8.4 Hz, 3H), 6.94 (d, J = 8.0 Hz, 2H), 6.79 (d, J = 8.0 Hz, 1H), 5.32–5.24 (m, 1H), 3.79 (s, 1H), 2.26 (s, 3H), 1.54 (d, J = 6.8 Hz, 3H), 1.21 (s, 9H). ^{13}C NMR (101 MHz, Chloroform-d) δ 167.3, 145.2, 143.8, 143.4, 140.6, 139.5, 137.3, 135.9, 134.5, 133.8, 130.9, 130.7, 130.1, 129.9, 129.5, 128.9, 128.8, 128.6, 128.0, 127.9, 127.7, 127.1, 126.9, 126.44, 126.39, 126.2, 125.39, 125.36, 119.9, 99.3, 89.1, 64.3, 56.7, 49.0, 24.9, 22.8, 22.0, 20.9. HRMS (ESI): *m/z* calcd. For $C_{45}H_{42}N_2NaO_2S$ [M + Na]$^+$ 697.2860, found 697.2836. $[\alpha]_D^{25}$ = –114.9 (c = 0.61, $CHCl_3$).

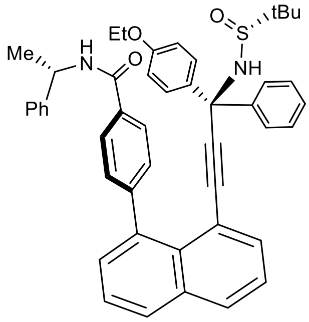

4a-III

4-(8-((*R*)-3-(((*R*)-tert-butylsulfinyl)amino)-3-(4-ethoxyphenyl)-3-phenylprop-1-yn-1-yl)naphthalen-1-yl)-*N*-((*S*)-1-phenylethyl)benzamide (4a-III). Purified by using a flash column chromatography (PE/EA = 5/1); isolated yield = 65%, 45.8 mg; yellow liquid. ^{1}H NMR (400 MHz, Chloroform-d) δ 7.97 (dd, J = 7.2, 1.6 Hz, 1H), 7.93 (dd, J = 8.4, 1.2 Hz, 1H), 7.86 (dd, J = 8.2, 1.4 Hz, 1H), 7.52–7.45 (m, 5H), 7.37 (t, J = 7.8 Hz, 3H), 7.33–7.29 (m, 4H), 7.27–7.24 (m, 1H), 7.24–7.18 (m, 3H), 7.10–7.04 (m, 4H), 6.52 (dd, J = 6.6, 2.2 Hz, 2H), 5.30–5.22 (m, 1H), 3.94–3.85 (m, 2H), 3.71 (s, 1H), 1.57 (d, J = 6.8 Hz, 3H), 1.38 (t, J = 7.0 Hz, 3H), 1.21 (s, 9H). ^{13}C NMR (101 MHz, Chloroform-d) δ 167.3, 158.4, 145.0, 143.8, 143.7, 139.5, 135.8, 135.3, 134.5, 133.9, 130.8, 130.8, 130.1, 130.0, 129.3, 128.8, 128.5, 128.3, 128.0, 127.8, 127.7, 127.2, 126.7, 126.4, 126.3, 125.4, 125.4, 119.8, 113.8, 99.2, 89.0, 64.3, 63.5, 56.7, 49.1, 26.9, 22.8, 21.8, 14.8. HRMS (ESI): *m/z* calcd. For $C_{46}H_{44}N_2NaO_3S$ [M + Na]$^+$ 727.2965, found 727.2943. $[\alpha]_D^{25}$ = –80.6 (c = 0.40, $CHCl_3$).

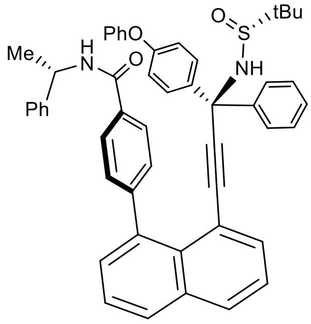

4a-IV

4-(8-((*R*)-3-(((*R*)-tert-butylsulfinyl)amino)-3-(4-phenoxyphenyl)-3-phenylprop-1-yn-1-yl)naphthalen-1-yl)-*N*-((*S*)-1-phenylethyl)benzamide (4a-IV). Purified by using a flash column chromatography (PE/EA = 5/1); isolated yield = 57%, 43.0 mg; yellow liquid. ^{1}H NMR (400 MHz, Chloroform-d) δ 7.99 (dd, J = 7.2, 1.6 Hz, 1H), 7.93 (dd, J = 8.2, 1.4 Hz, 1H), 7.87 (dd, J = 8.2, 1.4 Hz, 1H), 7.53–7.47 (m, 3H), 7.42 (dd, J = 7.8, 1.8 Hz, 1H), 7.39–7.30 (m, 9H), 7.29–7.27 (m, 2H), 7.25–

7.21 (m, 3H), 7.16–7.11 (m, 4H), 7.03 (dd, *J* = 8.6, 1.0 Hz, 2H), 6.87 (d, *J* = 8.4 Hz, 1H), 6.71 (dd, *J* = 6.4, 2.0 Hz, 2H), 5.30–5.23 (m, 1H), 3.74 (s, 1H), 1.55 (d, *J* = 7.2 Hz, 3H), 1.22 (s, 9H). ^{13}C NMR (101 MHz, Chloroform-d) δ 167.1, 156.9, 156.7, 145.2, 143.6, 143.3, 139.4, 138.0, 135.8, 134.5, 134.0, 131.0, 130.8, 130.0, 129.8, 129.4, 128.9, 128.6, 128.5, 128.1, 127.9, 127.8, 127.2, 126.6, 126.3, 126.2, 125.4, 123.6, 119.4, 117.8, 99.1, 64.1, 56.8, 49.1, 22.8, 21.9. HRMS (ESI): *m/z* calcd. For $C_{50}H_{45}N_2O_3S$ [M + H]$^+$ 753.3146, found 753.3122. $[\alpha]_D^{25}$ = –34.7 (*c* = 0.90, $CHCl_3$).

2.9 General procedure for the synthesis of substrates **5a**

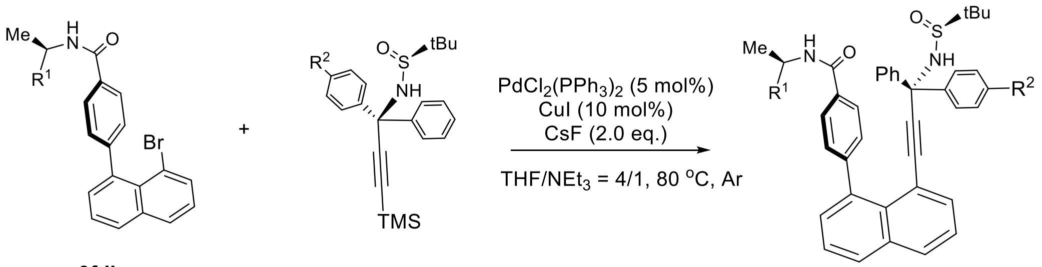

3f-II **1f** **5a**

The mixture of **3f-II** (0.1 mmol, 1.0 equiv.), $PdCl_2(PPh_3)_2$ (3.5 mg, 5 mol%), CuI (1.9 mg, 10 mol%), and CsF (30.4 mg, 0.2 mmol, 2.0 equiv.) in THF/NEt_3 (2 mL) was added the mixture of **1f** (41.4 mg, 0.1 mmol, 1 equiv.) under Ar atmosphere. Then the mixture was heated in an oil bath at 80 °C and stirred for 12 h. After cooling to the temperature, to the mixture was added water (10 mL) and extracted with ethyl acetate (3×10 mL). The organic layer was dried over $MgSO_4$, and concentrated in vacuo. The residue was purified by column chromatography on silica gel (PE/EA = 5 : 1) to afford the desired product **5a**.

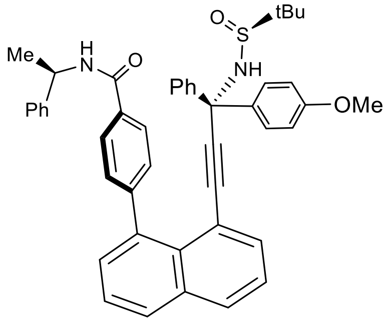

5a-I

4-(8-((*R*)-3-(((*S*)-tert-butylsulfinyl)amino)-3-(4-methoxyphenyl)-3-phenylprop-1-yn-1-yl)naphthalen-1-yl)-*N*-((*R*)-1-phenylethyl)benzamide (5a-I): Purified by using a flash column chromatography (PE/EA = 5/1); isolated yield = 50%, 34.5 mg; yellow liquid. ^{1}H NMR (400 MHz, Chloroform-*d*) δ 8.01 (dd, *J* = 7.2, 1.2 Hz, 1H), 7.97–7.94 (m, 1H), 7.91–7.88 (m, 1H), 7.55–7.49 (m, 2H), 7.48–7.45 (m, 1H), 7.43–7.41 (m, 1H), 7.40–7.34 (m, 7H), 7.31–7.29 (m, 2H), 7.26–7.22 (m, 4H), 7.13–7.11 (m, 2H), 6.91 (d, *J* = 8.0 Hz, 1H), 6.71–6.68 (m, 2H), 5.30 (q, *J* = 7.2 Hz, 1H), 3.80 (s, 1H), 3.78 (s, 3H), 1.58 (d, *J* = 6.9 Hz, 3H), 1.24 (s, 9H). ^{13}C NMR (101 MHz, Chloroform-*d*) δ 167.3, 159.1, 145.1, 143.8, 143.6, 139.5, 136.0, 135.5, 134.5, 133.9, 130.8, 130.8, 130.1, 130.0, 129.4, 128.9, 128.5, 128.4, 128.0, 127.9, 127.7, 127.1, 126.5, 126.4, 126.3, 125.4, 119.8, 113.4, 99.3, 99.1, 64.2, 56.6, 55.4, 49.1, 22.8, 22.0. HRMS (ESI-TOF) m/z: [M+H]$^+$ Calcd for $C_{45}H_{43}N_2O_3S$ 691.2989; Found 691.2969. $[a]_D^{25}$ = –109.3 (c = 0.80, $CHCl_3$).

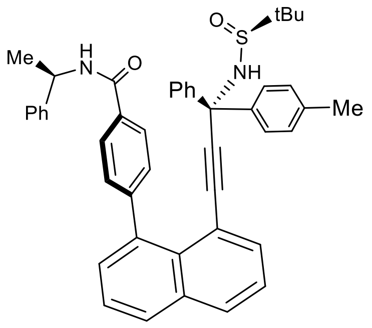

5a-II

4-(8-((*R*)-3-(((*S*)-tert-butylsulfinyl)amino)-3-phenyl-3-(p-tolyl)prop-1-yn-1-yl)naphthalen-1-yl)-*N*-((*R*)-1-phenylethyl)benzamide (5a-II): Purified by using a flash column chromatography (PE/EA = 15/1); isolated yield =44%, 29.6 mg; yellow liquid. ^{1}H NMR (400 MHz, Chloroform-*d)* δ 8.01 (d, J = 7.1 Hz, 1H), 7.95 (d, J = 8.1 Hz, 1H), 7.89 (d, J = 8.0 Hz, 1H), 7.55–7.49 (m, 3H), 7.46–7.41 (m, 2H), 7.40–7.35 (m, 4H), 7.32–7.30 (m, 2H), 7.26–7.22 (m, 4H), 7.17–7.11 (m, 3H), 7.06–7.02 (m, 3H), 6.90 (d, J = 8.0 Hz, 1H), 5.35–5.28 (m, 1H), 3.79 (s, 1H), 2.31 (s, 3H), 1.58 (d, J = 7.0 Hz, 3H), 1.25 (s, 9H). ^{13}C NMR (101 MHz, Chloroform-*d)* δ 167.3, 145.1, 143.9, 143.7, 140.4, 139.5, 137.5, 135.9, 134.5, 133.9, 130.9, 130.7, 130.1, 129.9, 129.4, 128.8, 128.8, 128.5, 128.1, 127.8, 127.5, 127.1, 126.9, 126.5, 126.4, 125.4, 119.9, 99.3, 89.0, 64.2, 56.7, 49.0, 22.8, 22.1, 21.0. HRMS (ESI-TOF) m/z: [M+H] $^{+}$ Calcd for $C_{45}H_{43}N_2O_2S$ 675.3040; Found 675.3019. $[a]_D^{25}$ = –54.0 (c = 0.30, $CHCl_3$).

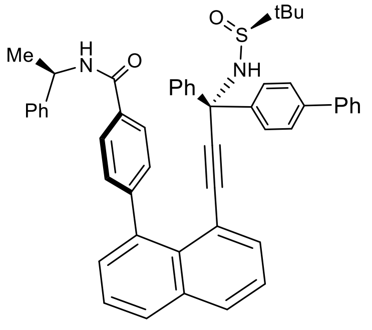

5a-III

4-(8-((*R*)-3-([1,1'-biphenyl]-4-yl)-3-(((*S*)-tert-butylsulfinyl)amino)-3-phenylprop-1-yn-1-yl)naphthalen-1-yl)-*N*-((*R*)-1-phenylethyl)benzamide (5a-III): Purified by using a flash column chromatography (PE/EA = 5/1); isolated yield = 46%, 33.9 mg; yellow liquid. ^{1}H NMR (400 MHz, Chloroform-*d)* δ 8.06–8.03 (m, 1H), 7.97 (d, J = 8.2 Hz, 1H), 7.90 (d, J = 7.4 Hz, 1H), 7.60 (d, J = 7.3 Hz, 2H), 7.57–7.51 (m, 3H), 7.50–7.44 (m, 3H), 7.43–7.37 (m, 8H), 7.34–7.29 (m, 5H), 7.28–7.26 (m, 4H), 7.12 (d, J = 7.8 Hz, 1H), 7.03 (d, J = 8.0 Hz, 1H), 5.34 (p, J = 7.1 Hz, 1H), 3.92 (s, 1H), 1.57 (d, J = 6.9 Hz, 3H), 1.28 (s, 9H). ^{13}C NMR (101 MHz, Chloroform-*d)* δ 167.4, 145.0, 143.8, 143.3, 142.6, 140.6, 136.0, 134.6, 134.0, 131.0, 130.8, 130.1, 129.3, 128.9, 128.5, 128.1, 127.9, 127.9, 127.5, 127.4, 127.2, 127.1, 126.9, 126.5, 126.4, 126.3, 125.5, 125.4, 99.0, 89.4, 64.4, 56.8, 49.0, 22.8, 22.0. HRMS (ESI-TOF) m/z: [M+H] $^{+}$ Calcd for $C_{50}H_{45}N_2O_2S$ 737.3196; Found 737.3169. $[a]_D^{25}$ = –149.7 (c = 0.50, $CHCl_3$).

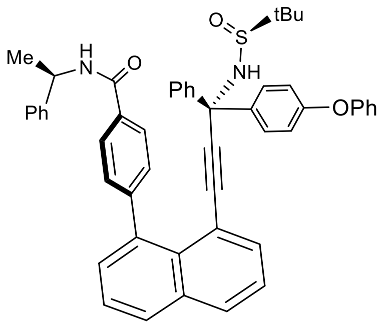

5a-IV

4-(8-((*R*)-3-(((*S*)-tert-butylsulfinyl)amino)-3-(4-phenoxyphenyl)-3-phenylprop-1-yn-1-yl)naphthalen-1-yl)-*N*-((*R*)-1-phenylethyl)benzamide (5a-IV): Purified by using a flash column chromatography (PE/EA = 15/1); isolated yield = 39%, 28.7 mg; yellow liquid. ^{1}H NMR (400 MHz, Chloroform-*d*) δ 8.01 (d, J = 7.2 Hz, 1H), 7.96 (d, J = 8.2 Hz, 1H), 7.90 (d, J = 7.8 Hz, 1H), 7.55–7.50 (m, 3H), 7.45–7.41 (m, 2H), 7.39–7.36 (m, 5H), 7.34–7.29 (m, 4H), 7.27–7.23 (m, 5H), 7.19–7.15 (m, 3H), 7.09 (d, J = 7.8 Hz, 2H), 6.90 (d, J = 8.0 Hz, 1H), 6.79 (d, J = 8.8 Hz, 2H), 5.34–5.27 (m, 1H), 3.82 (s, 1H), 1.56 (d, J = 8.0Hz, 3H), 1.25 (s, 9H). ^{13}C NMR (101 MHz, Chloroform-*d*) δ 167.3, 157.0, 156.7, 145.2, 143.7, 143.3, 139.4, 137.9, 135.9, 134.5, 134.0, 131.0, 130.8, 130.1, 129.9, 129.4, 128.9, 128.6, 128.5, 128.1, 127.9, 127.8, 127.1, 126.5, 126.3, 125.4, 125.4, 123.6, 119.7, 119.4, 117.8, 99.1, 89.2, 64.2, 56.7, 49.1, 22.8, 22.0. HRMS (ESI): *m/z* calcd. For $C_{50}H_{45}N_2O_3S$ $[M + H]^+$ 753.3146, found 753.3126. $[\alpha]_D^{25}$ = –57.2 (*c* = 0.50, $CHCl_3$).

3. NMR Spectrums

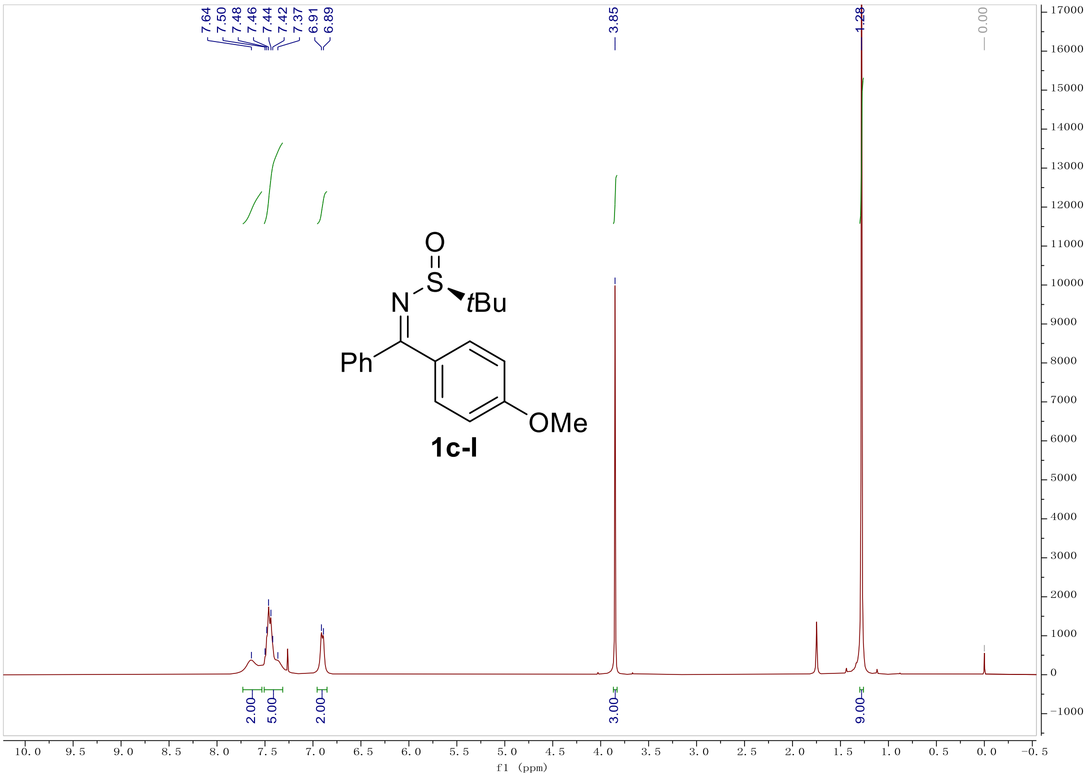

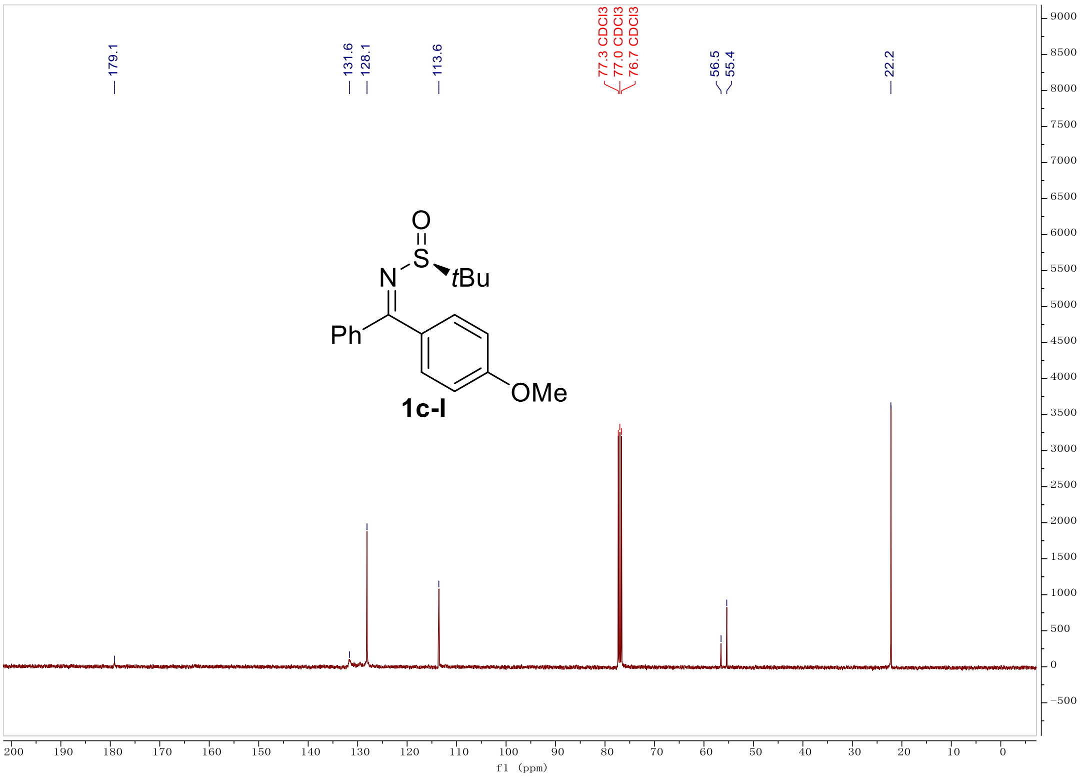

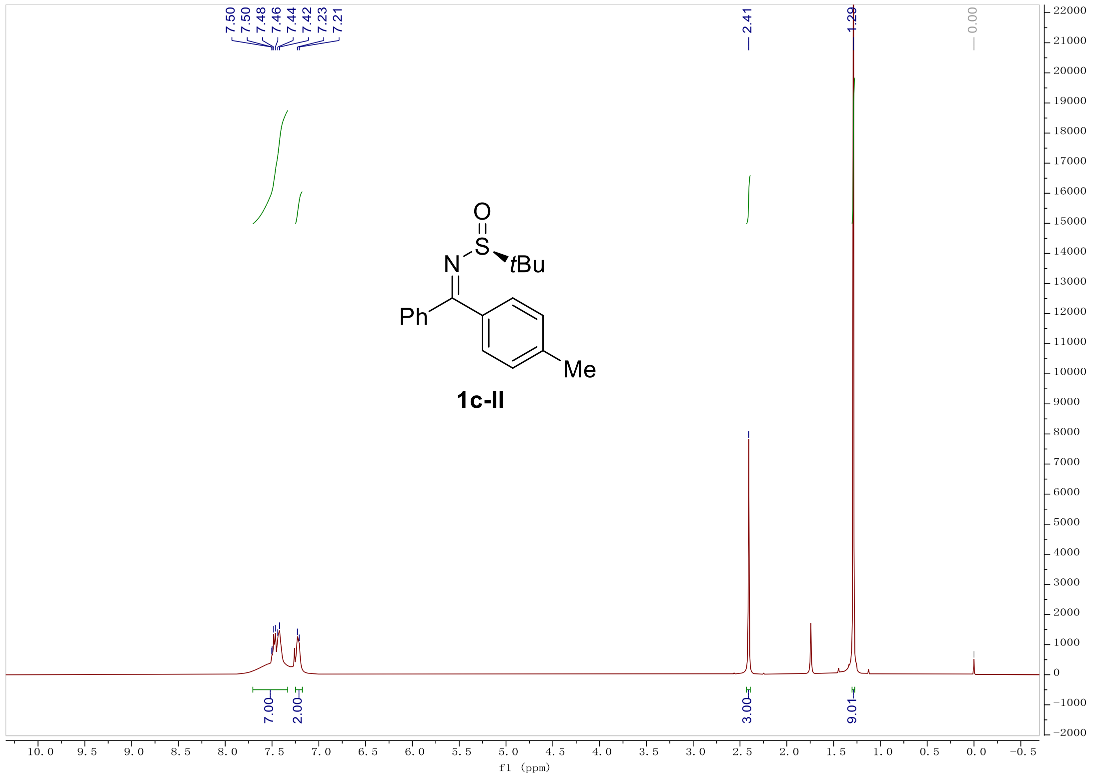
7.50
7.50
7.48
7.46
7.44
7.42
7.23
7.21
2.41
1.29
0.00
O
S
N
tBu
Ph
Me
1c-II
7.00
2.00
3.00
9.01
f1 (ppm)

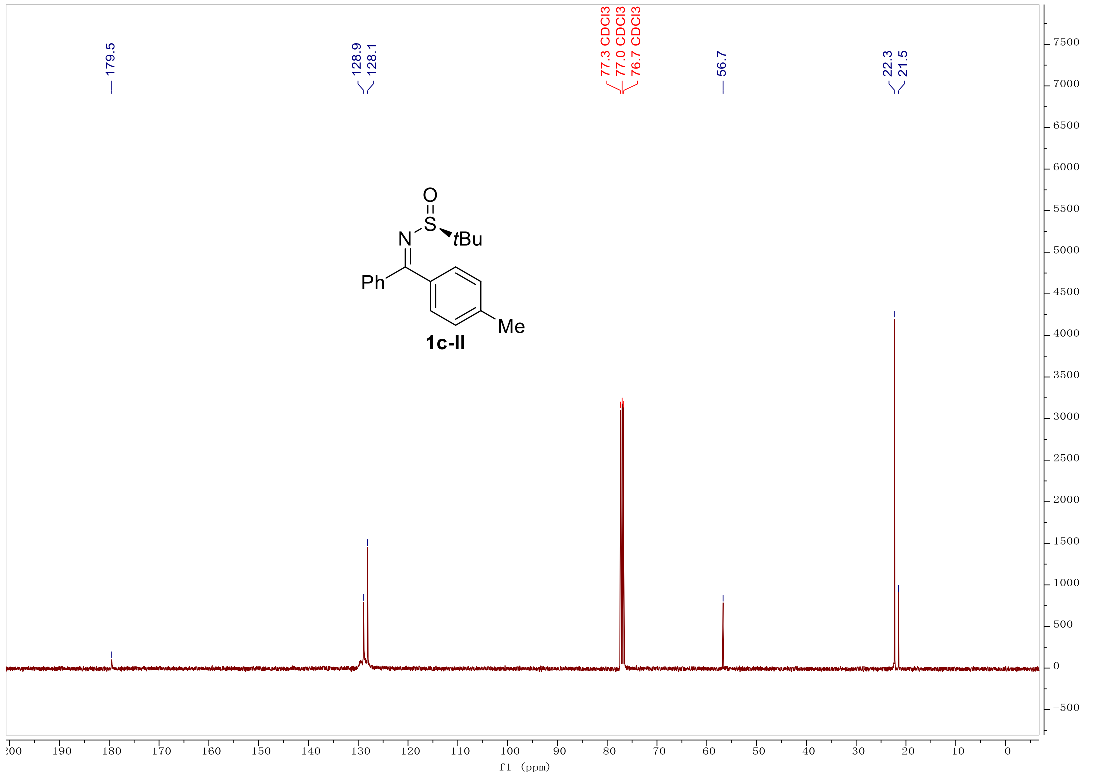
179.5
128.9
128.1
77.3 CDCl3
77.0 CDCl3
76.7 CDCl3
56.7
22.3
21.5
O
S
N
tBu
Ph
Me
1c-II
f1 (ppm)

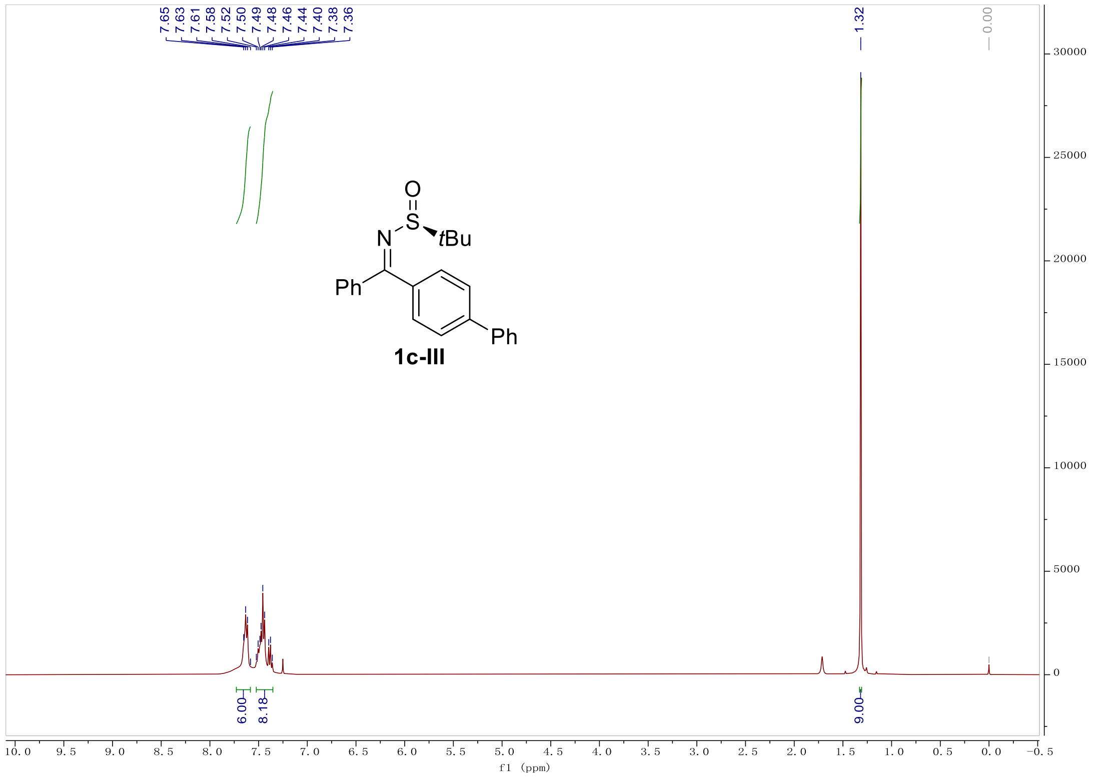
7.65
7.63
7.61
7.58
7.52
7.50
7.49
7.48
7.46
7.44
7.40
7.38
7.36
1.32
0.00
O
S
N
tBu
Ph
Ph
1c-III
6.00
8.18
9.00
f1 (ppm)

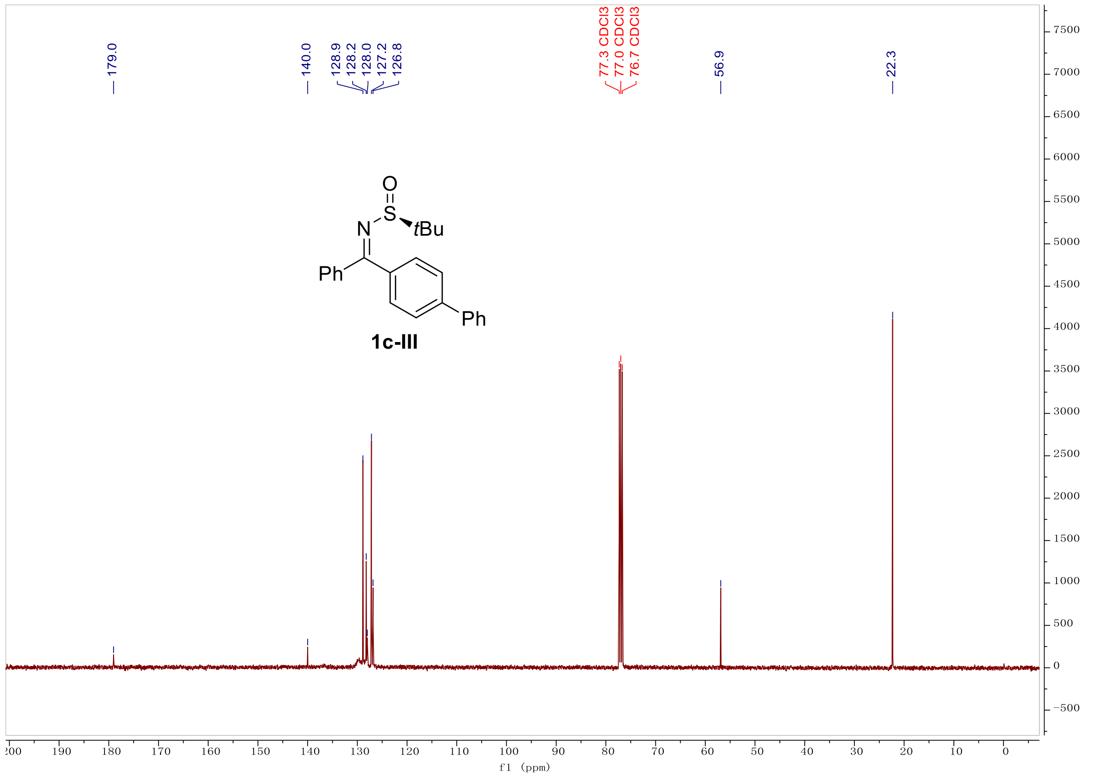
179.0
140.0
128.9
128.2
128.0
127.2
126.8
77.3 CDCl3
77.0 CDCl3
76.7 CDCl3
56.9
22.3
O
S
N
tBu
Ph
Ph
1c-III
f1 (ppm)

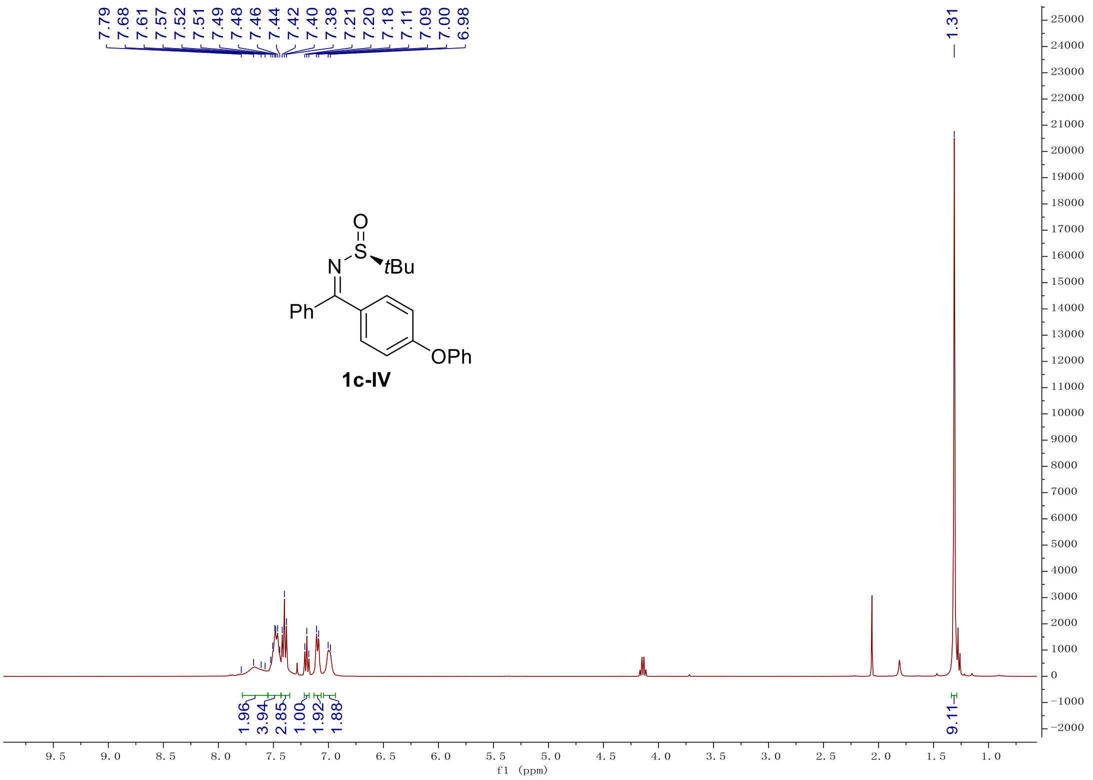
7.79
7.68
7.61
7.57
7.52
7.51
7.49
7.48
7.46
7.44
7.42
7.40
7.38
7.21
7.20
7.18
7.11
7.09
7.00
6.98
1.31
O
S
N
tBu
Ph
OPh
1c-IV
1.96
3.94
2.85
1.00
1.92
1.88
9.11
f1 (ppm)

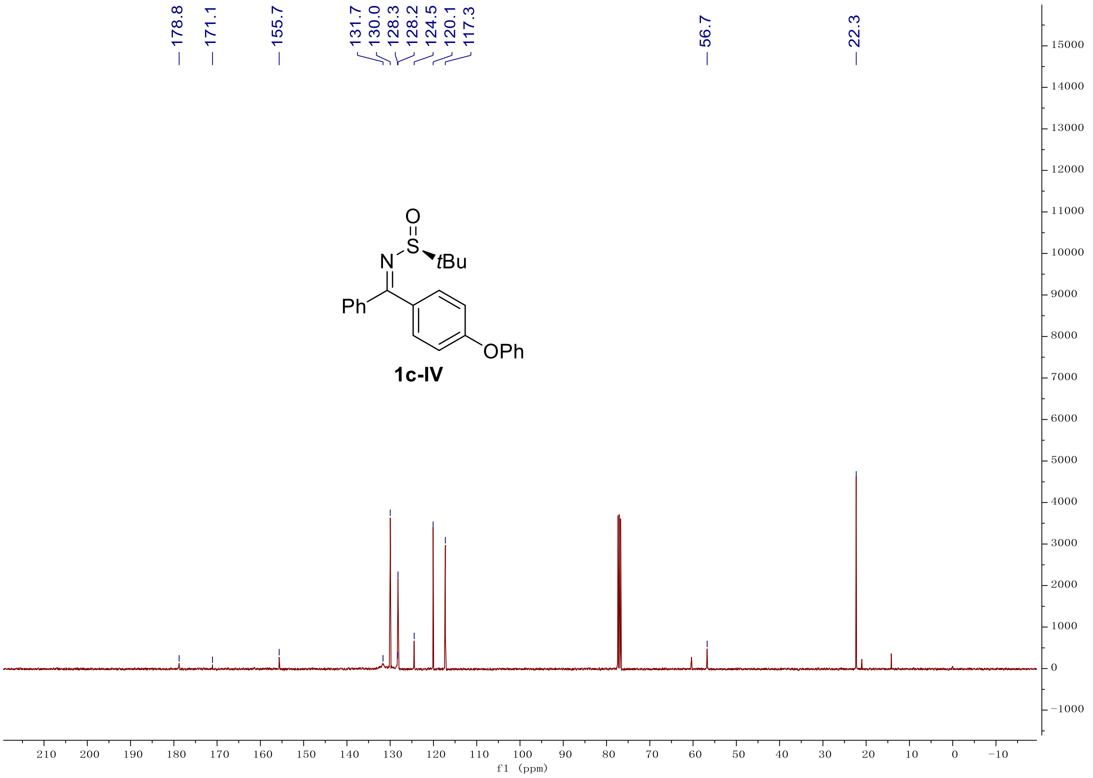
178.8
171.1
155.7
131.7
130.0
128.3
128.2
124.5
120.1
117.3
56.7
22.3
O
S
N
tBu
Ph
OPh
1c-IV
f1 (ppm)

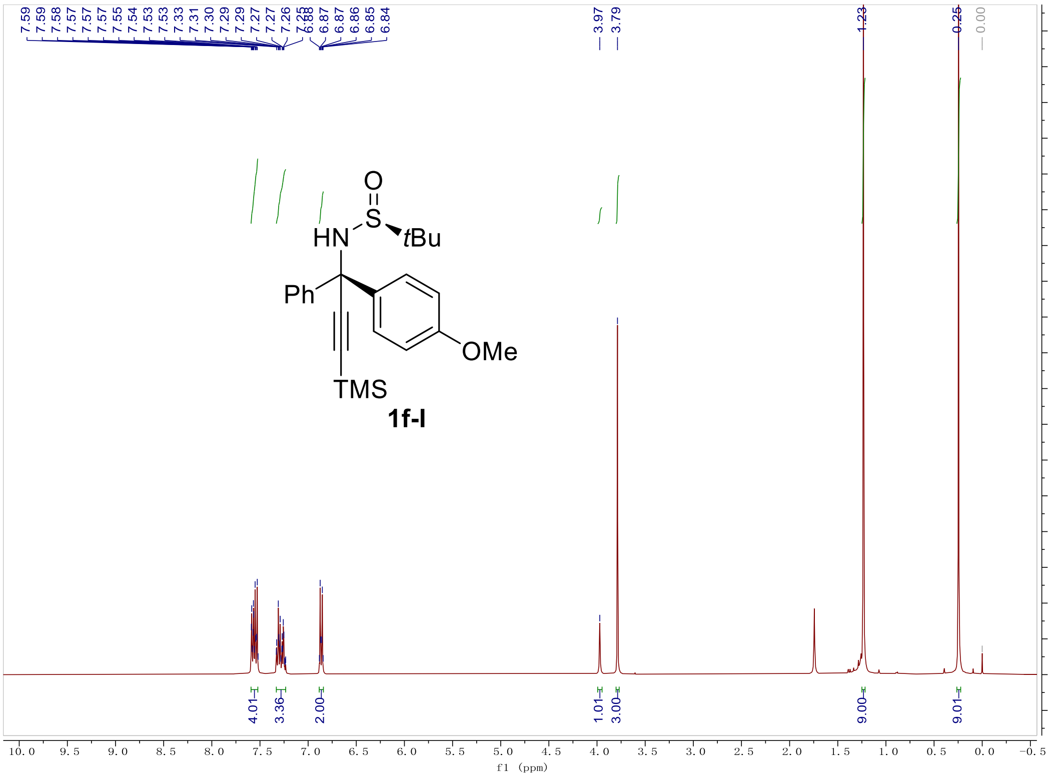
7.59
7.59
7.58
7.57
7.57
7.57
7.55
7.54
7.53
7.53
7.33
7.31
7.30
7.29
7.29
7.27
7.27
7.26
6.87
6.87
6.86
6.85
6.84
3.97
3.79
1.23
0.25
0.00
HN
S
O
tBu
Ph
OMe
TMS
1f-I
4.01
3.36
2.00
1.01
3.00
9.00
9.01
f1 (ppm)

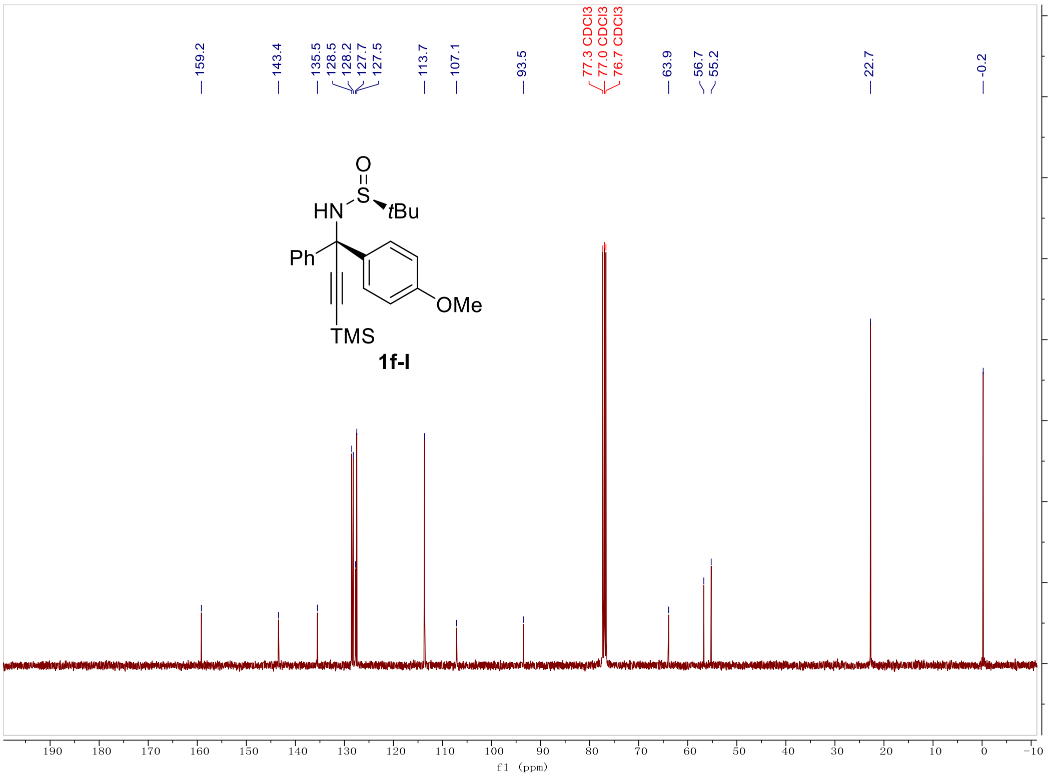
159.2
143.4
135.5
128.5
128.2
127.7
127.5
113.7
107.1
93.5
77.3 CDCl3
77.0 CDCl3
76.7 CDCl3
63.9
56.7
55.2
22.7
-0.2
HN
S
O
tBu
Ph
OMe
TMS
1f-I
f1 (ppm)

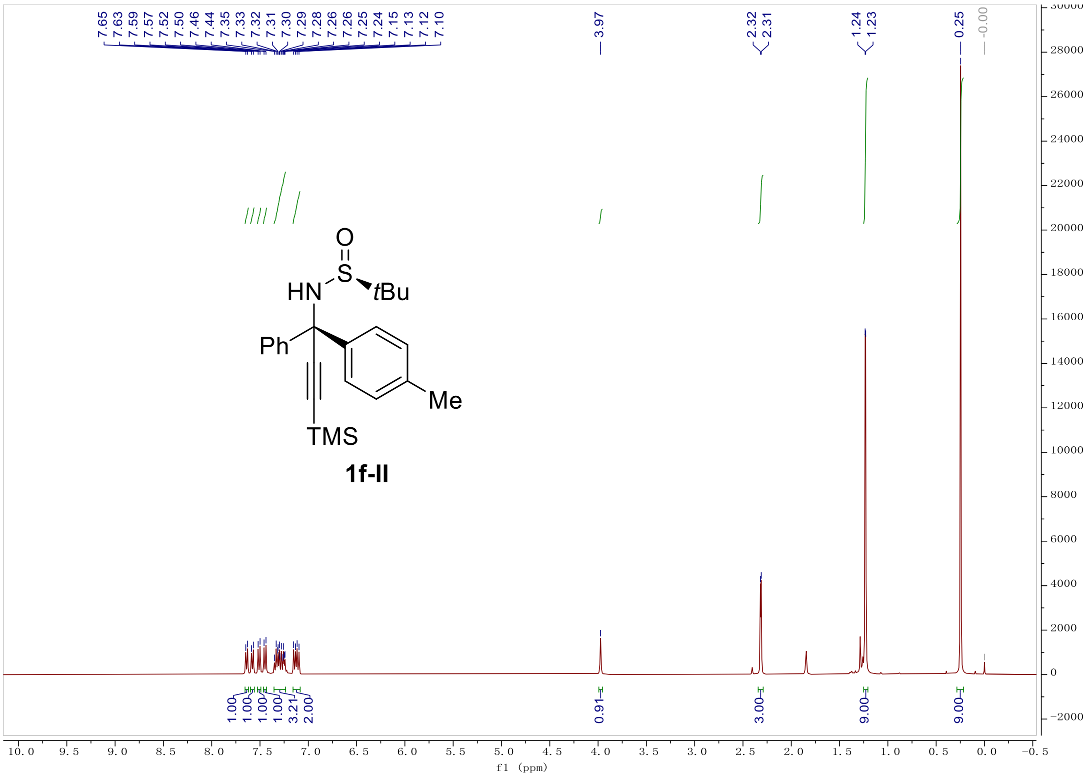
7.65
7.63
7.59
7.57
7.52
7.50
7.46
7.44
7.35
7.33
7.32
7.31
7.30
7.29
7.28
7.26
7.26
7.25
7.24
7.15
7.13
7.12
7.10
3.97
2.32
2.31
1.24
1.23
0.25
-0.00
HN
S
O
tBu
Ph
Me
TMS
1f-II
1.00
1.00
1.00
1.00
3.21
2.00
0.91
3.00
9.00
9.00
f1 (ppm)

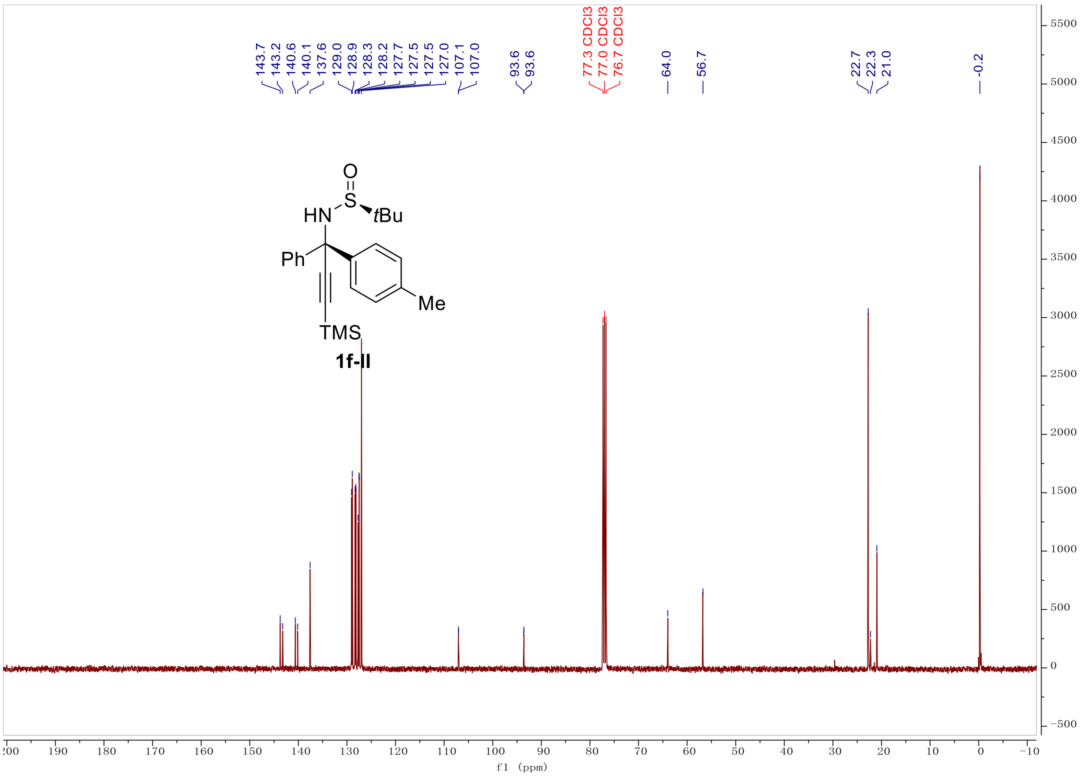
143.7
143.2
140.6
140.1
137.6
129.0
128.9
128.3
128.2
127.7
127.5
127.0
107.1
107.0
93.6
93.6
77.3 CDCl3
77.0 CDCl3
76.7 CDCl3
64.0
56.7
22.7
22.3
21.0
-0.2
HN
S
O
tBu
Ph
Me
TMS
1f-II
f1 (ppm)

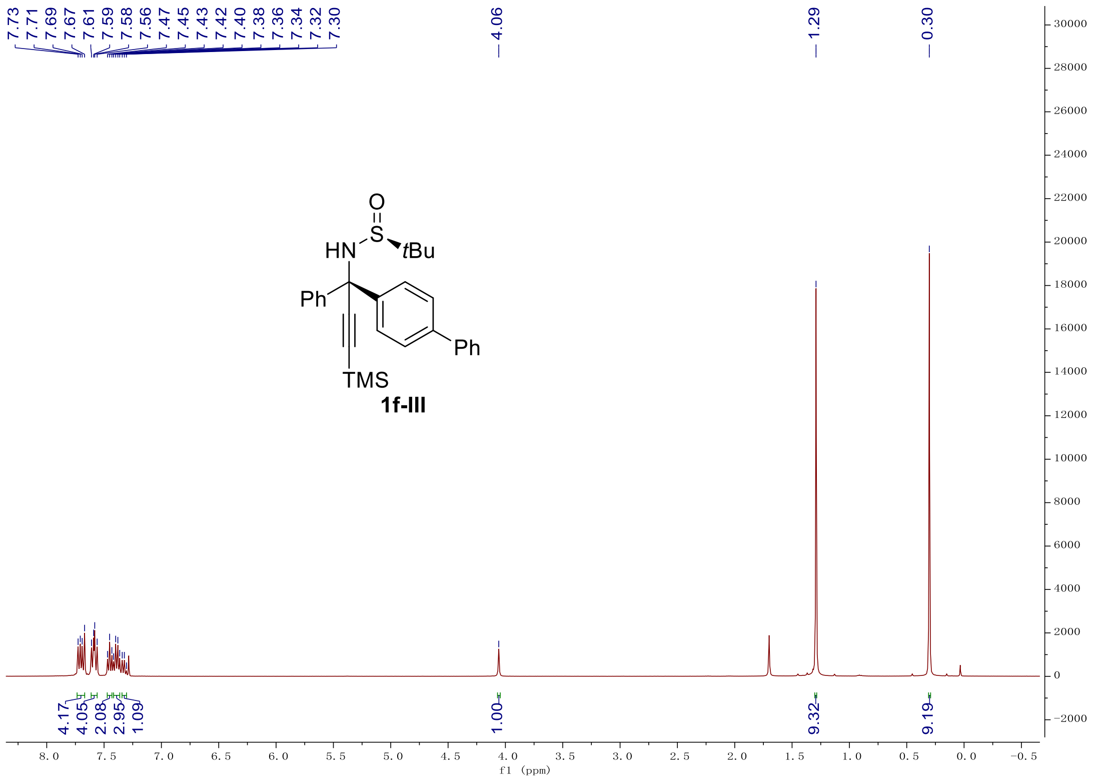
7.73 7.71 7.69 7.67 7.61 7.59 7.58 7.56 7.47 7.45 7.43 7.42 7.40 7.38 7.36 7.34 7.32 7.30
4.06
1.29
0.30
HN
S
O
tBu
Ph
Ph
TMS
1f-III
4.17 4.05 2.08 2.95 1.09
1.00
9.32
9.19
f1 (ppm)

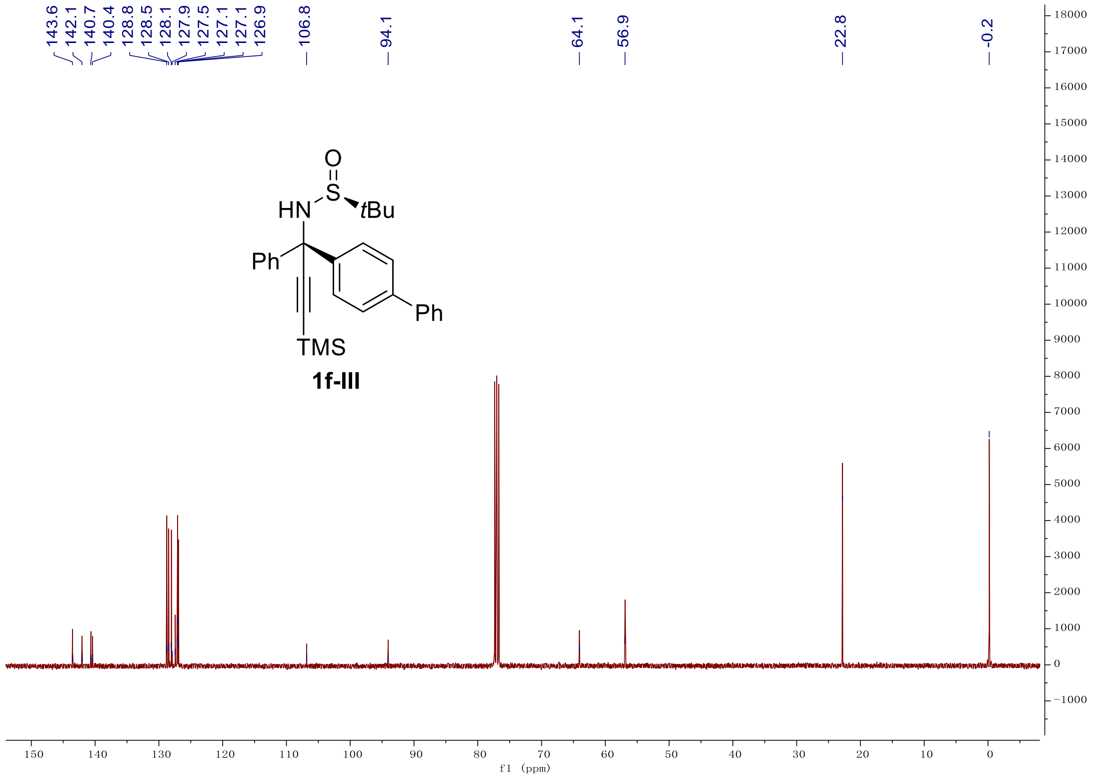
143.6 142.1 140.7 140.4 128.8 128.5 128.1 127.9 127.5 127.1 127.1 126.9
106.8
94.1
64.1
56.9
22.8
-0.2
HN
S
O
tBu
Ph
Ph
TMS
1f-III
f1 (ppm)

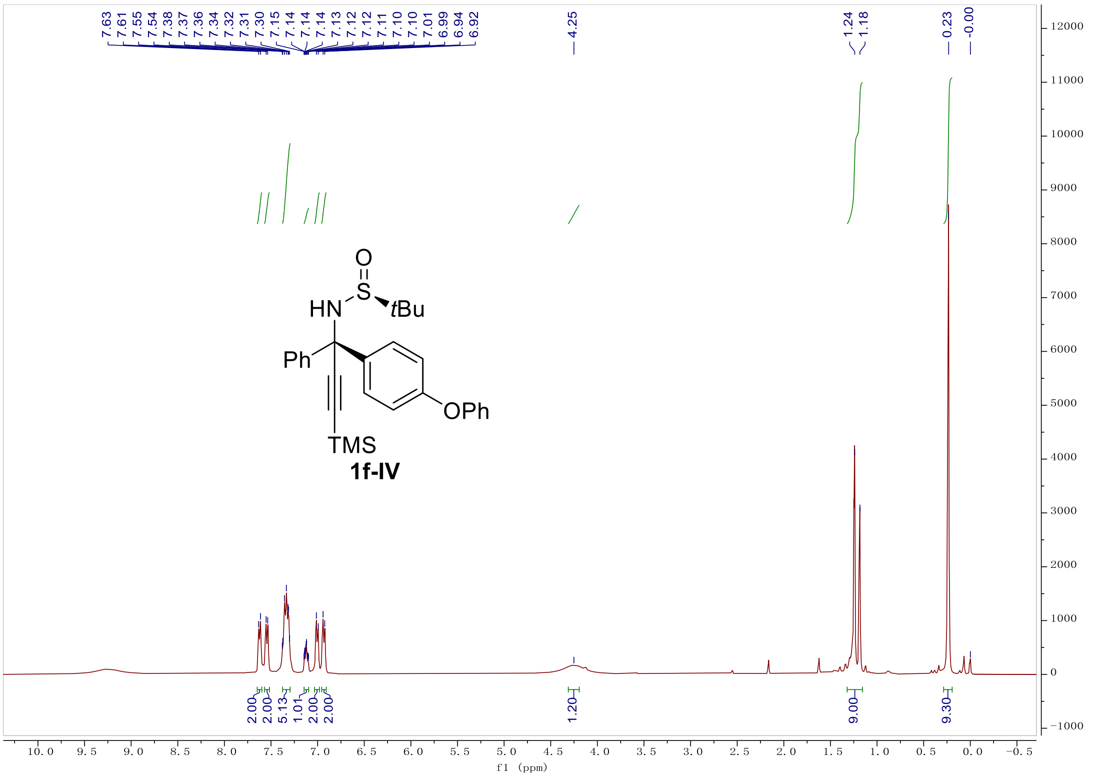
7.63
7.61
7.55
7.54
7.38
7.37
7.36
7.34
7.32
7.31
7.30
7.15
7.14
7.14
7.14
7.13
7.12
7.12
7.11
7.10
7.10
7.01
6.99
6.94
6.92
4.25
1.24
1.18
0.23
-0.00
HN
S
O
tBu
Ph
OPh
TMS
1f-IV
2.00
2.00
5.13
1.01
2.00
2.00
1.20
9.00
9.30
f1 (ppm)

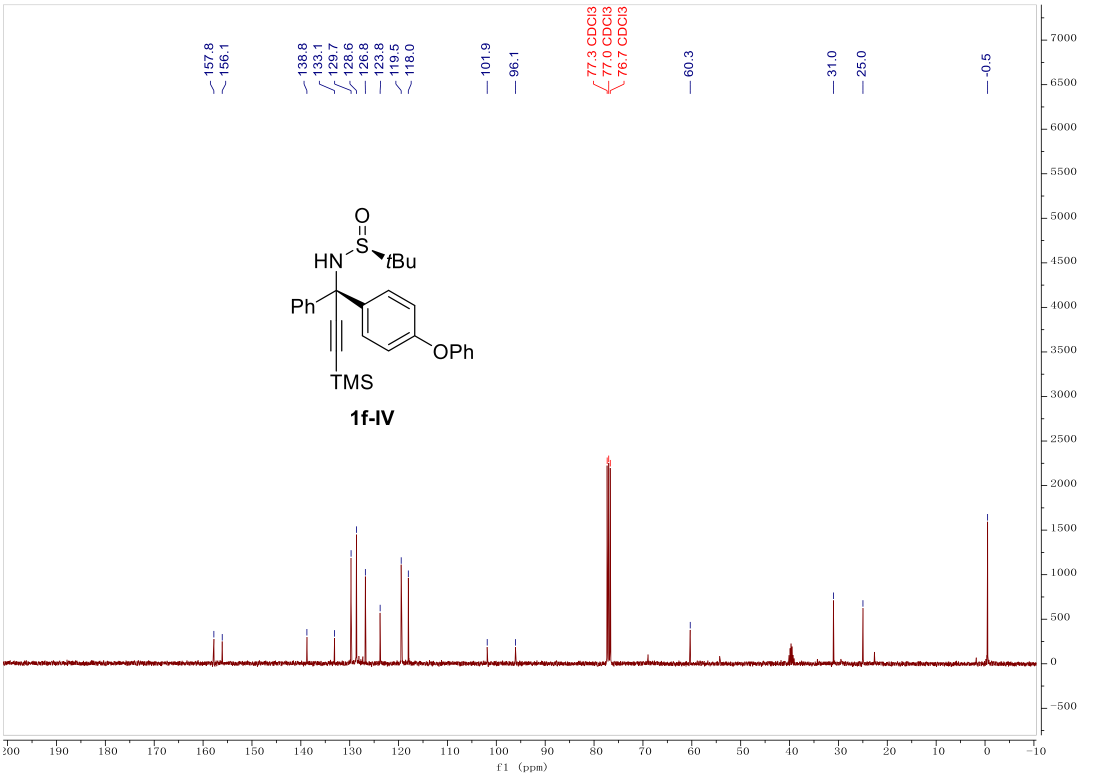
157.8
156.1
138.8
133.1
129.7
128.6
126.8
123.8
119.5
118.0
101.9
96.1
77.3 CDCl3
77.0 CDCl3
76.7 CDCl3
60.3
31.0
25.0
-0.5
HN
S
O
tBu
Ph
OPh
TMS
1f-IV
f1 (ppm)

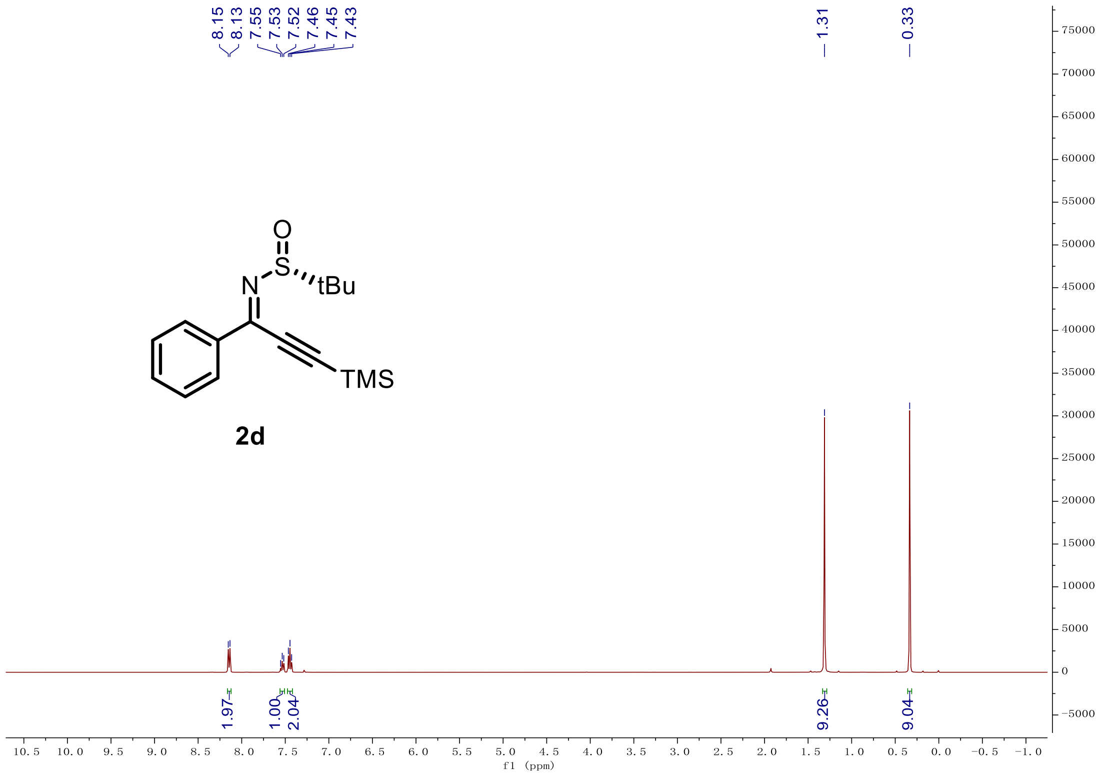
8.15
8.13
7.55
7.53
7.52
7.46
7.45
7.43
1.31
0.33
tBu
TMS
2d
1.97
1.00
2.04
9.26
9.04
f1 (ppm)

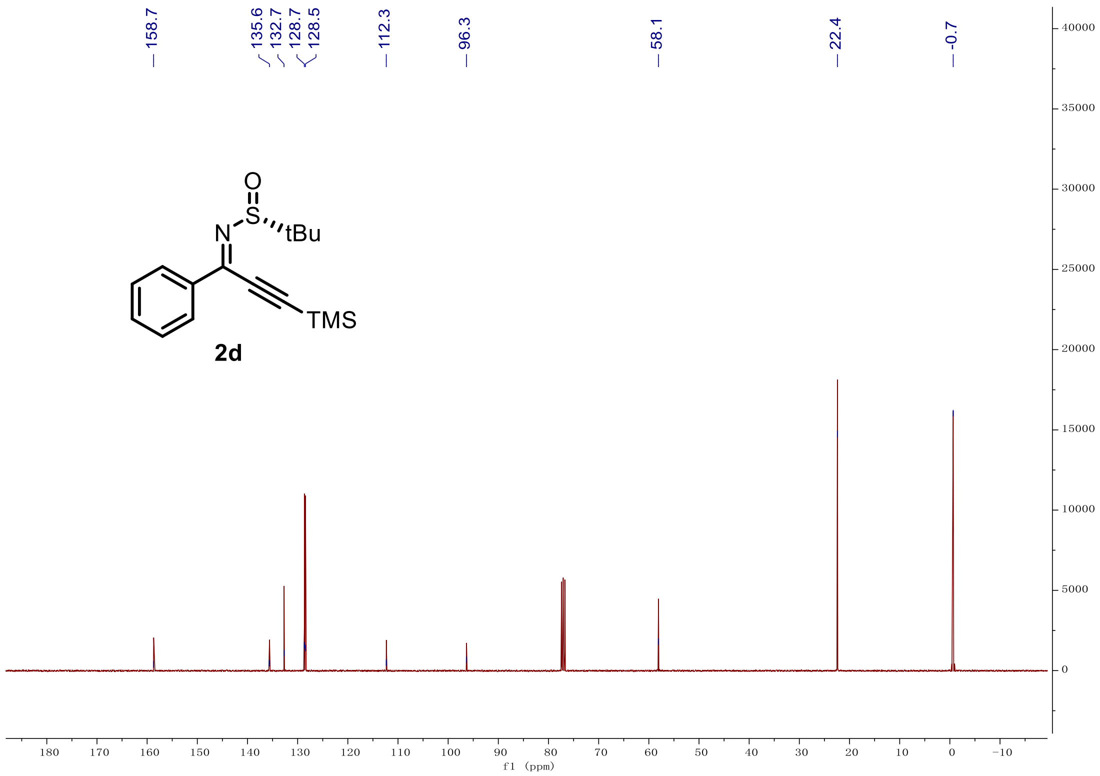
158.7
135.6
132.7
128.7
128.5
112.3
96.3
58.1
22.4
-0.7
tBu
TMS
2d
f1 (ppm)

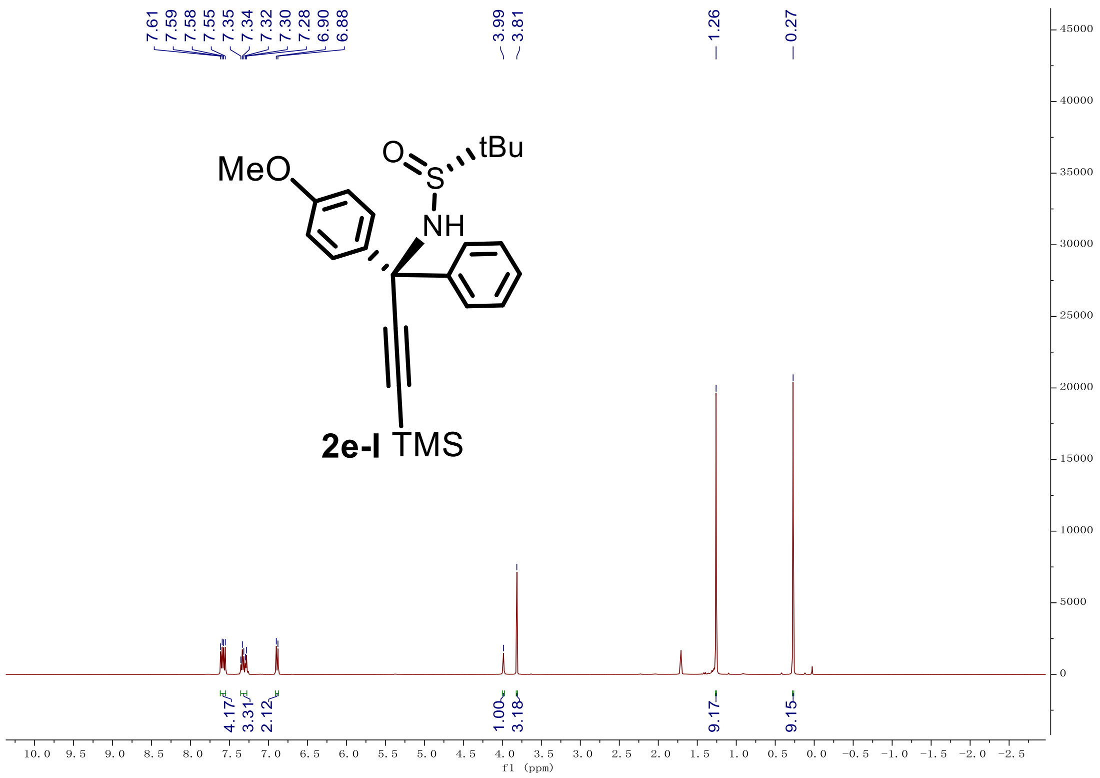

7.61 7.59 7.58 7.55 7.35 7.34 7.32 7.30 7.28 6.90 6.88
3.99 3.81
1.26
0.27
MeO
O
S
tBu
NH
2e-I
TMS
4.17 3.31 2.12
1.00 3.18
9.17
9.15
f1 (ppm)

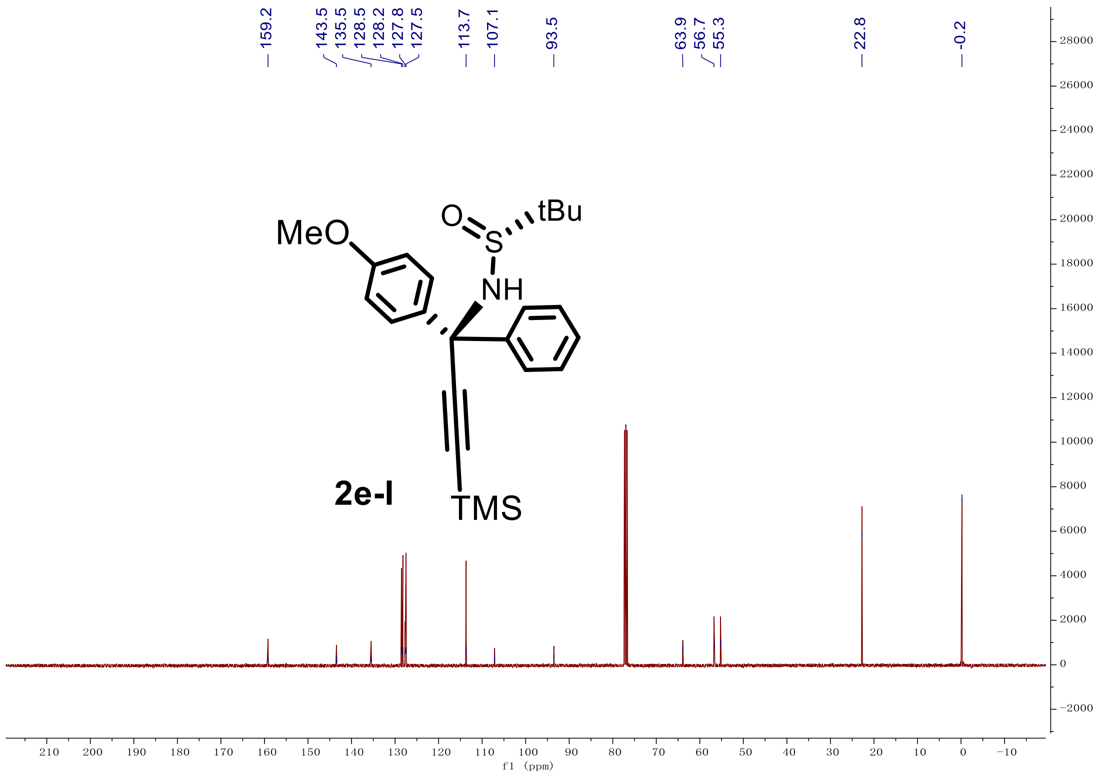

159.2
143.5 135.5 128.5 128.2 127.8 127.5
113.7
107.1
93.5
63.9 56.7 55.3
22.8
-0.2
MeO
O
S
tBu
NH
2e-I
TMS
f1 (ppm)

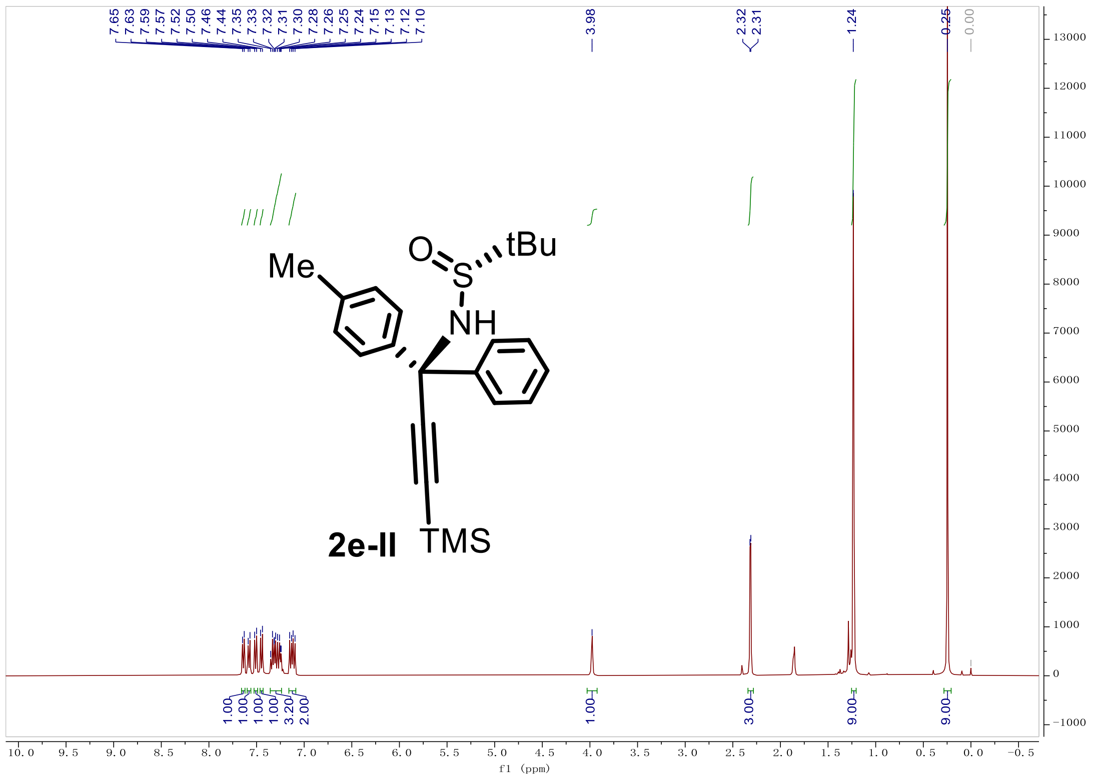
7.65
7.63
7.59
7.57
7.52
7.50
7.46
7.44
7.35
7.33
7.32
7.31
7.30
7.28
7.26
7.25
7.24
7.15
7.13
7.12
7.10
3.98
2.32
2.31
1.24
0.25
0.00
Me
O
S
tBu
NH
2e-II
TMS
1.00
1.00
1.00
1.00
3.20
2.00
1.00
3.00
9.00
9.00
f1 (ppm)

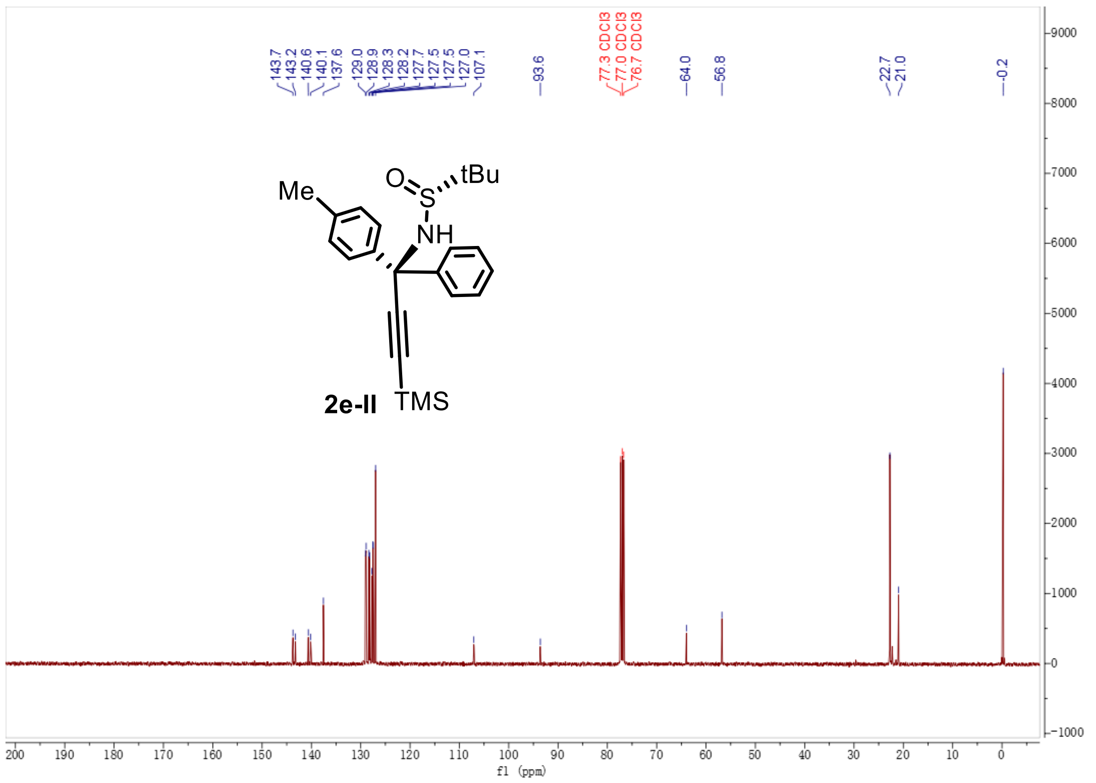
143.7
143.2
140.6
140.1
137.6
129.0
128.9
128.3
128.2
127.7
127.5
127.5
127.0
107.1
93.6
77.3 CDCl3
77.0 CDCl3
76.7 CDCl3
64.0
56.8
22.7
21.0
-0.2
Me
O
S
tBu
NH
2e-II
TMS
f1 (ppm)

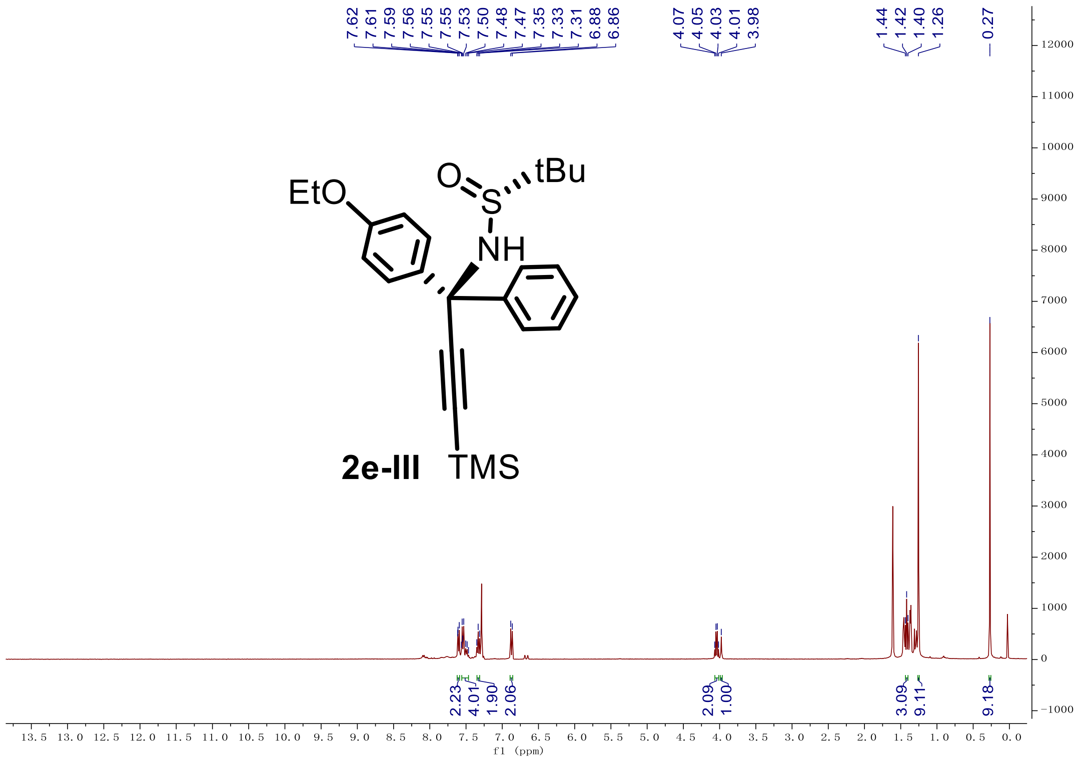
7.62
7.61
7.59
7.56
7.55
7.55
7.53
7.50
7.48
7.47
7.35
7.33
7.31
6.88
6.86
4.07
4.05
4.03
4.01
3.98
1.44
1.42
1.40
1.26
0.27
EtO
O
S
tBu
NH
2e-III
TMS
2.23
4.01
1.90
2.06
2.09
1.00
3.09
9.11
9.18
f1 (ppm)

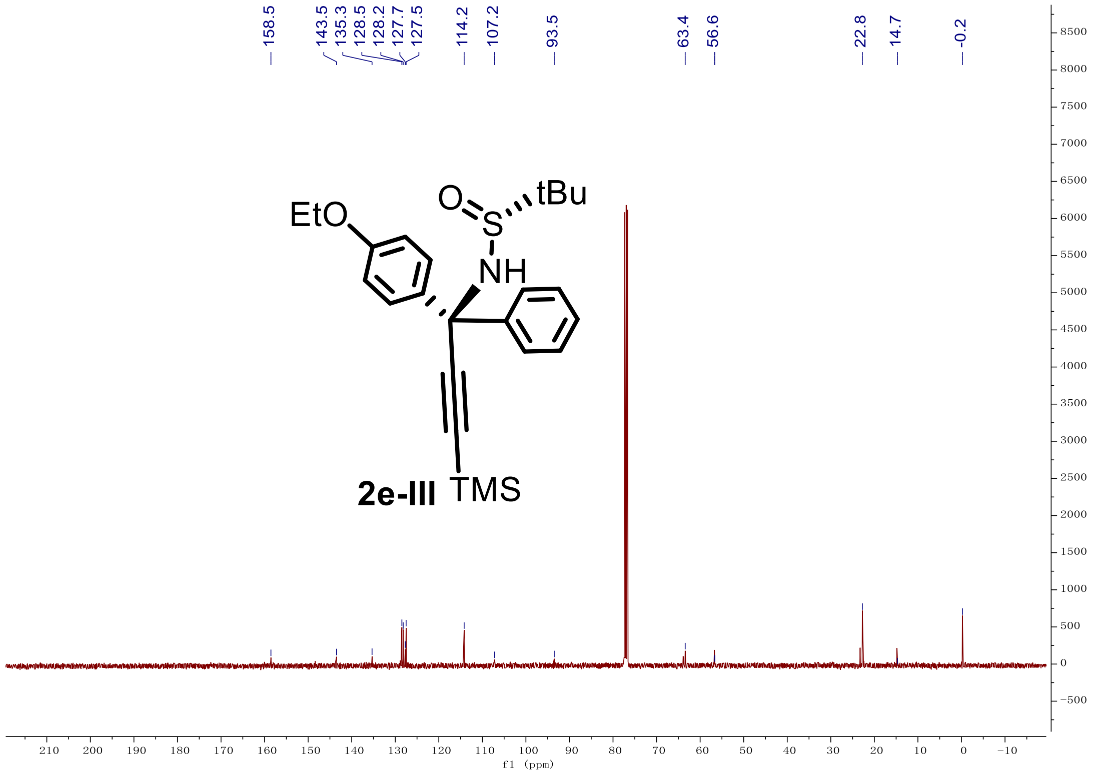
158.5
143.5
135.3
128.5
128.2
127.7
127.5
114.2
107.2
93.5
63.4
56.6
22.8
14.7
-0.2
EtO
O
S
tBu
NH
2e-III
TMS
f1 (ppm)

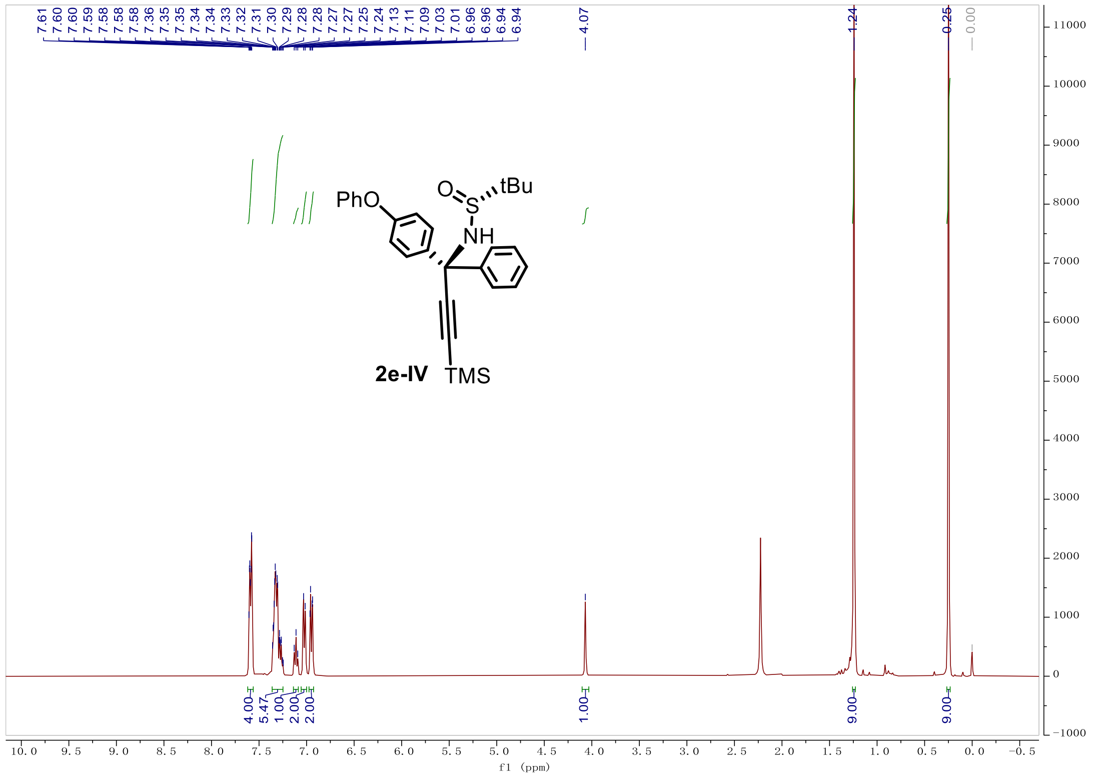
7.61
7.60
7.60
7.59
7.58
7.58
7.58
7.36
7.35
7.35
7.34
7.33
7.32
7.31
7.30
7.29
7.28
7.28
7.27
7.27
7.25
7.24
7.13
7.11
7.09
7.03
7.01
6.96
6.96
6.94
6.94
4.07
1.24
0.25
0.00
PhO
O
S
tBu
NH
2e-IV
TMS
4.00
5.47
1.00
2.00
2.00
1.00
9.00
9.00
f1 (ppm)

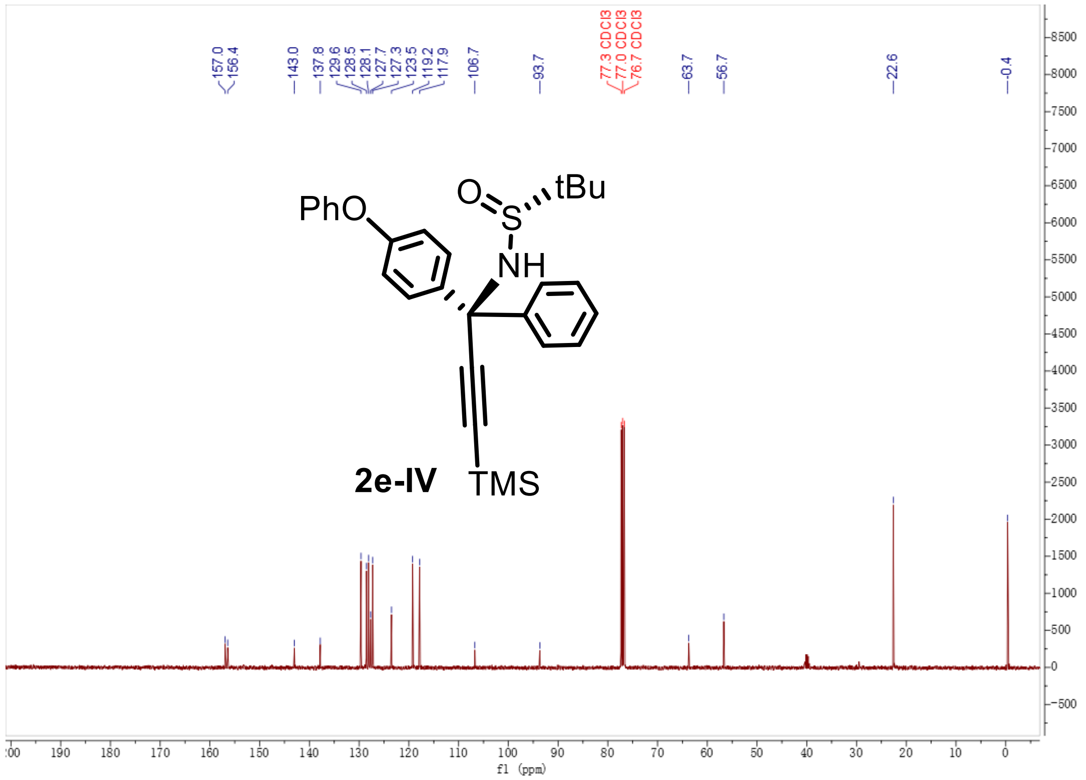
157.0
156.4
143.0
137.8
129.6
128.5
128.1
127.7
127.3
123.5
119.2
117.9
106.7
93.7
77.3 CDCl3
77.0 CDCl3
76.7 CDCl3
63.7
56.7
22.6
-0.4
PhO
O
S
tBu
NH
2e-IV
TMS
f1 (ppm)

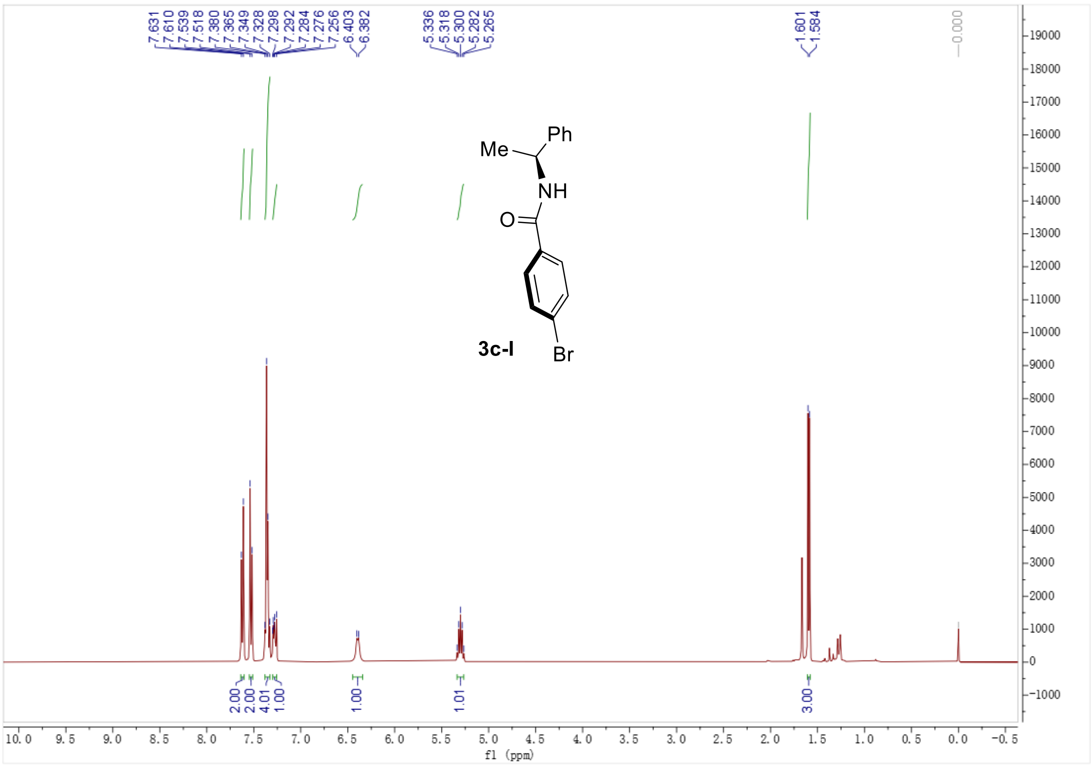
7.631
7.610
7.539
7.518
7.380
7.365
7.349
7.328
7.298
7.292
7.284
7.276
7.256
6.403
6.382
5.336
5.318
5.300
5.282
5.265
1.601
1.584
0.000
Me
Ph
NH
O
3c-I
Br
2.00
2.00
4.01
1.00
1.00
1.01
3.00
f1 (ppm)

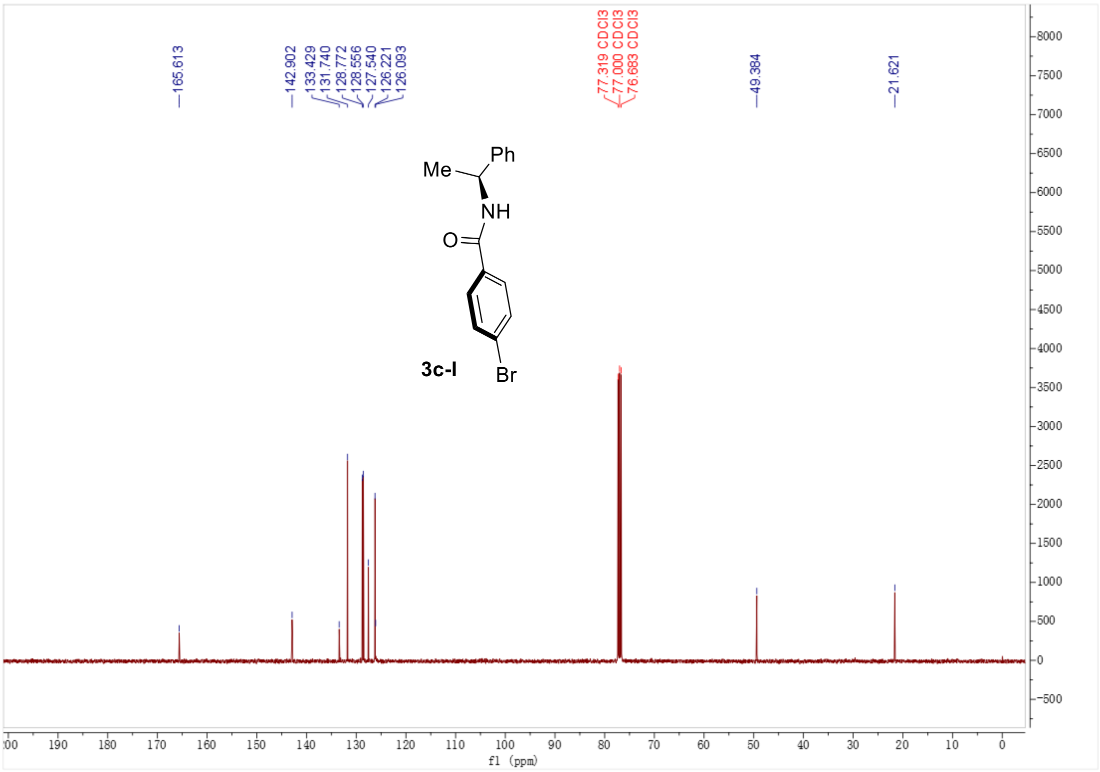
165.613
142.902
133.429
131.740
128.772
128.556
127.540
126.221
126.093
77.319 CDCl3
77.000 CDCl3
76.683 CDCl3
49.384
21.621
Me
Ph
NH
O
3c-I
Br
f1 (ppm)

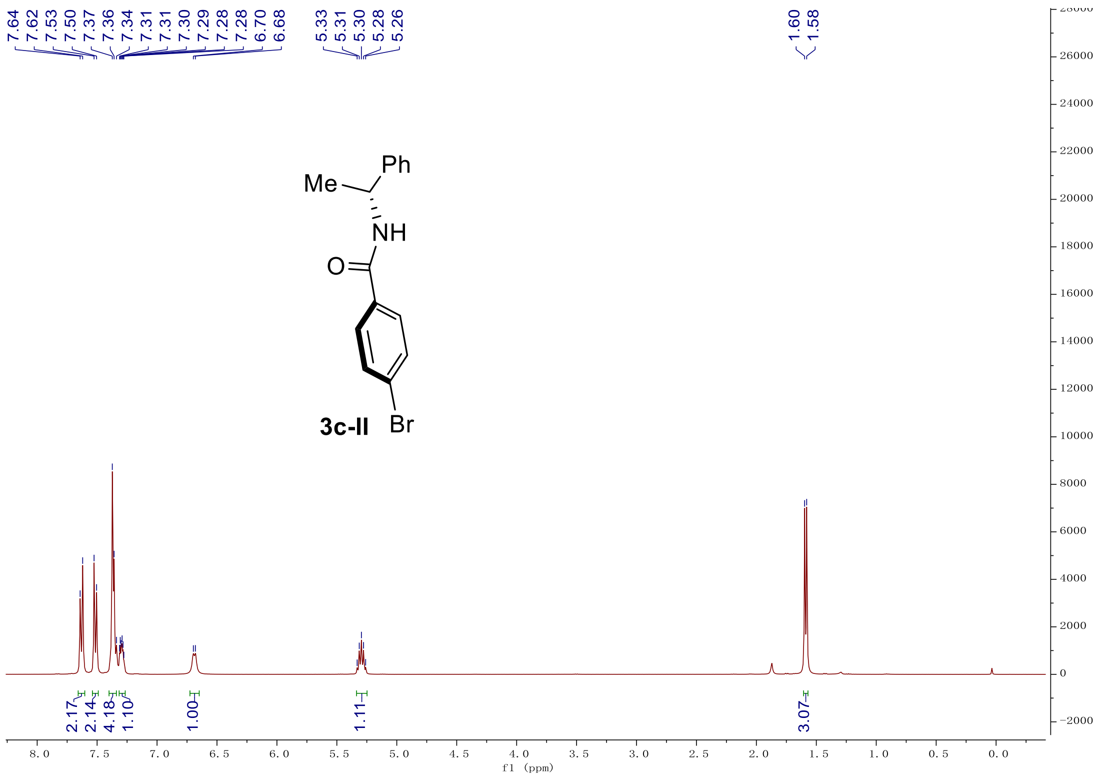
7.64
7.62
7.53
7.50
7.37
7.36
7.34
7.31
7.31
7.30
7.29
7.28
7.28
6.70
6.68
5.33
5.31
5.30
5.28
5.26
1.60
1.58
Ph
Me
NH
O
Br
3c-II
2.17
2.14
4.18
1.10
1.00
1.11
3.07
f1 (ppm)

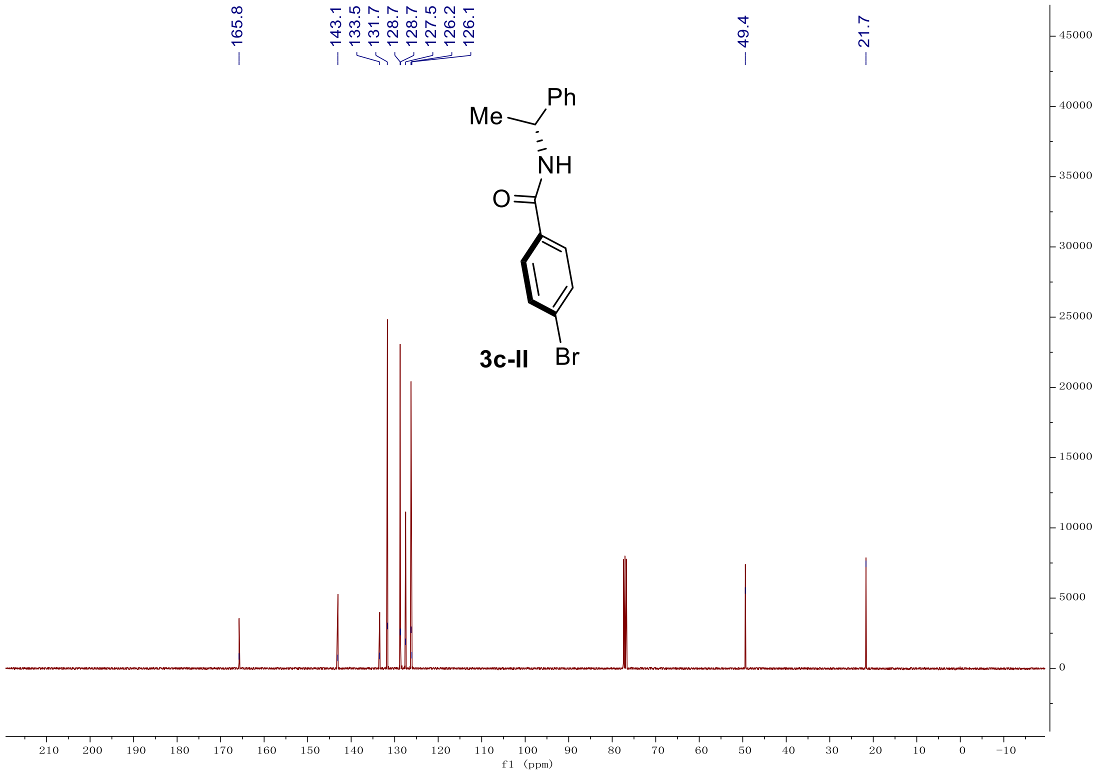
165.8
143.1
133.5
131.7
128.7
128.7
127.5
126.2
126.1
49.4
21.7
Ph
Me
NH
O
Br
3c-II
f1 (ppm)

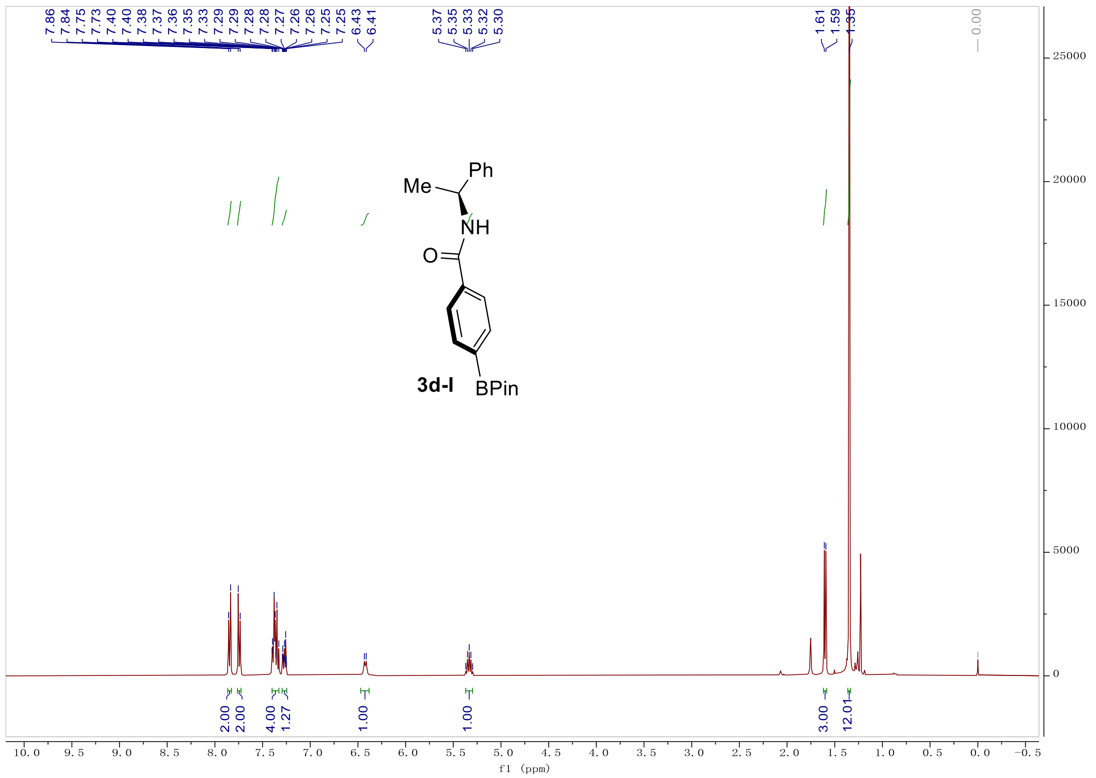
7.86
7.84
7.75
7.73
7.40
7.40
7.38
7.37
7.36
7.35
7.33
7.29
7.29
7.28
7.28
7.27
7.26
7.26
7.25
7.25
6.43
6.41
5.37
5.35
5.33
5.32
5.30
1.61
1.59
1.35
0.00
Me
Ph
NH
O
3d-I
BPin
2.00
2.00
4.00
1.27
1.00
1.00
3.00
12.01
f1 (ppm)

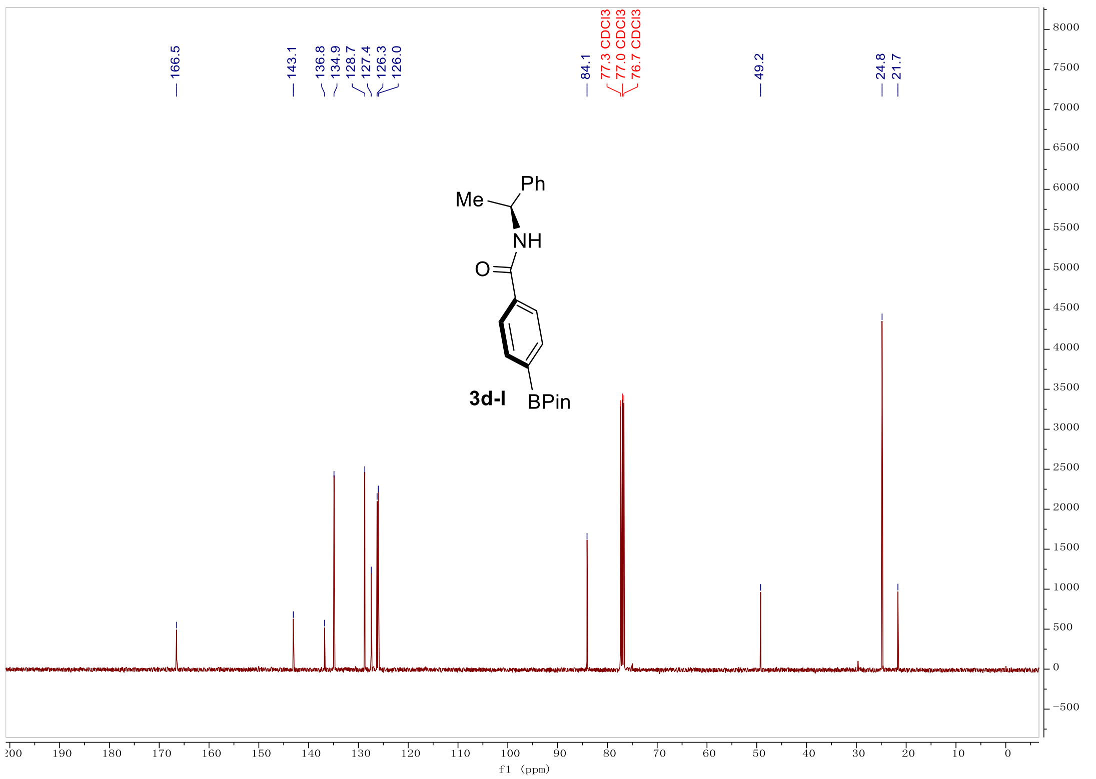
166.5
143.1
136.8
134.9
128.7
127.4
126.3
126.0
84.1
77.3 CDCl3
77.0 CDCl3
76.7 CDCl3
49.2
24.8
21.7
Me
Ph
NH
O
3d-I
BPin
f1 (ppm)

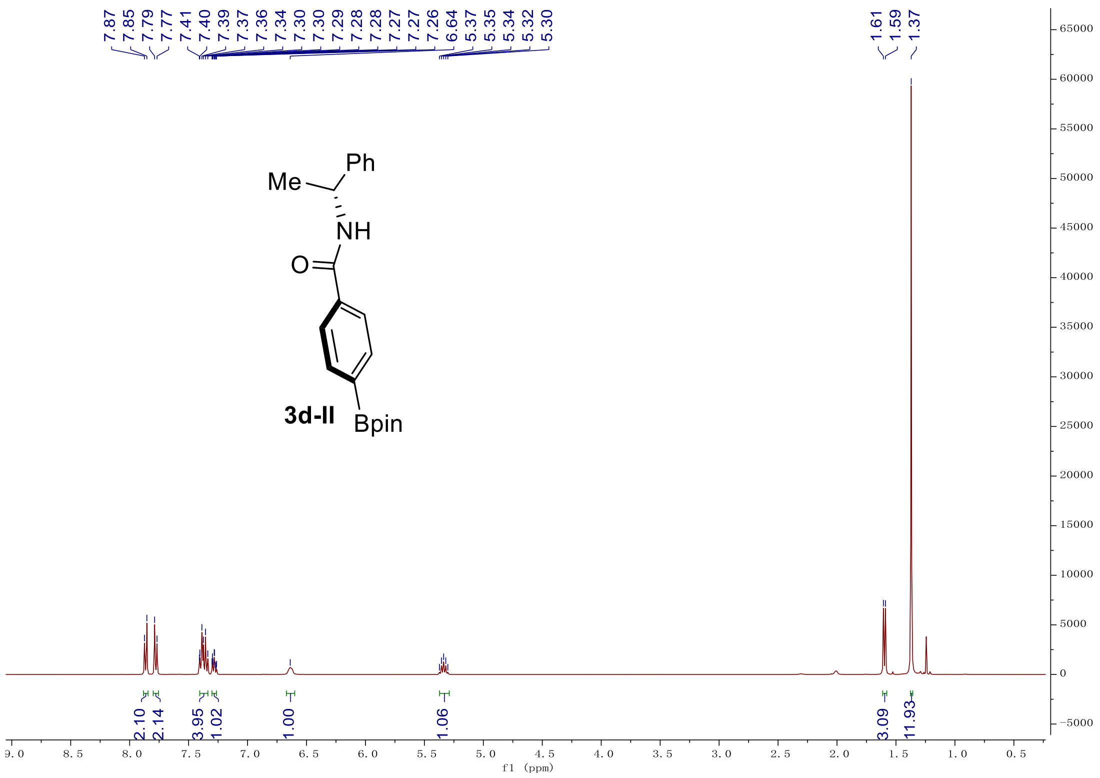
7.87
7.85
7.79
7.77
7.41
7.40
7.39
7.37
7.36
7.34
7.30
7.30
7.29
7.28
7.28
7.27
7.27
7.26
6.64
5.37
5.35
5.34
5.32
5.30
1.61
1.59
1.37
Ph
Me
NH
O
3d-II
Bpin
2.10
2.14
3.95
1.02
1.00
1.06
3.09
11.93
f1 (ppm)

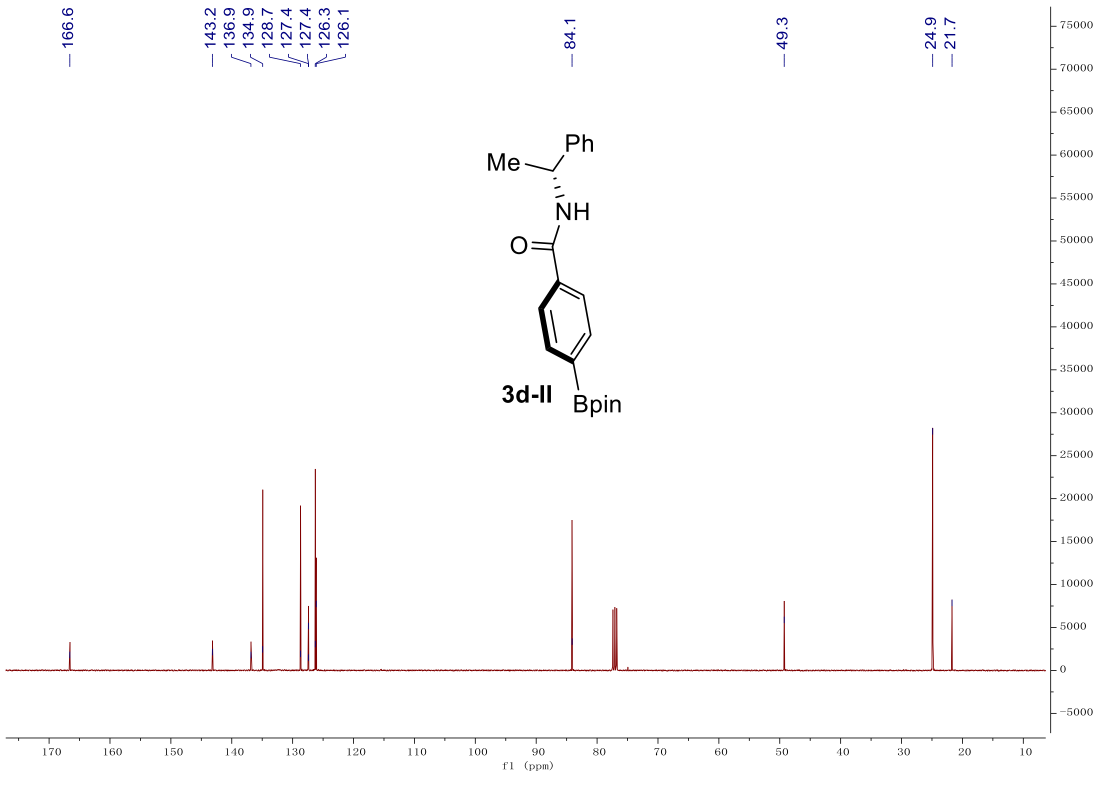
166.6
143.2
136.9
134.9
128.7
127.4
127.4
126.3
126.1
84.1
49.3
24.9
21.7
Ph
Me
NH
O
3d-II
Bpin
f1 (ppm)

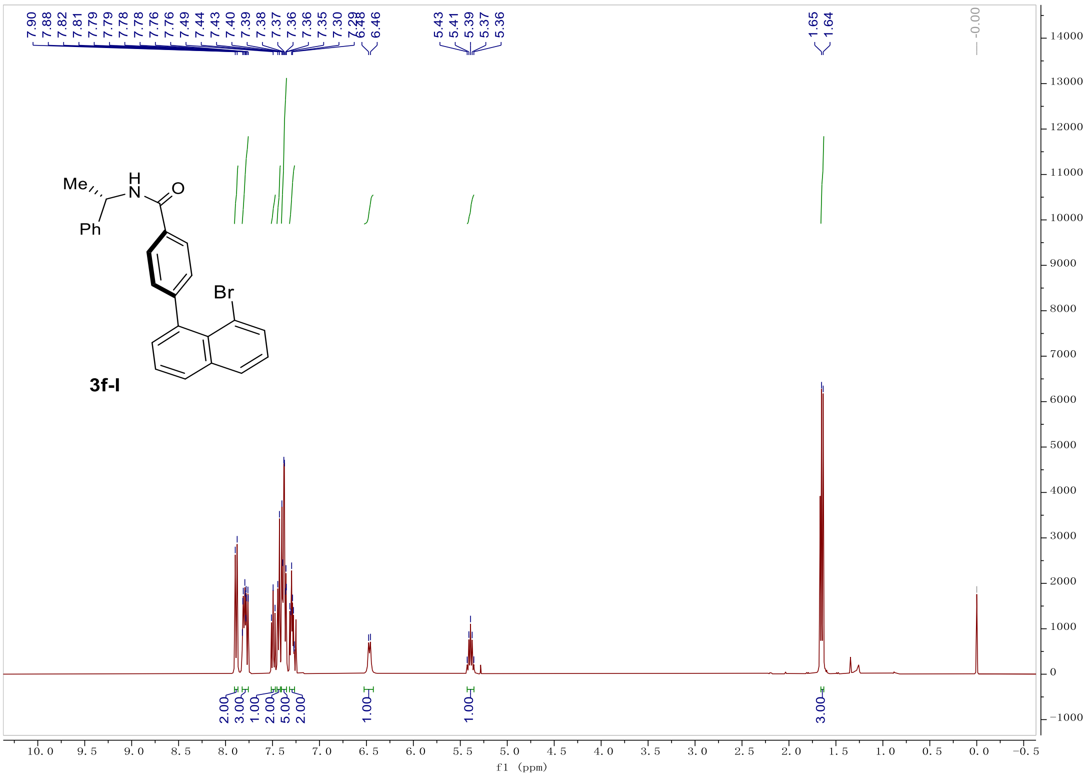
7.90
7.88
7.82
7.81
7.79
7.79
7.78
7.78
7.76
7.49
7.44
7.43
7.40
7.39
7.38
7.37
7.36
7.36
7.35
7.30
7.29
6.48
6.46
5.43
5.41
5.39
5.37
5.36
1.65
1.64
-0.00
Me
H
N
O
Ph
Br
3f-I
2.00
3.00
1.00
2.00
5.00
2.00
1.00
1.00
3.00
f1 (ppm)

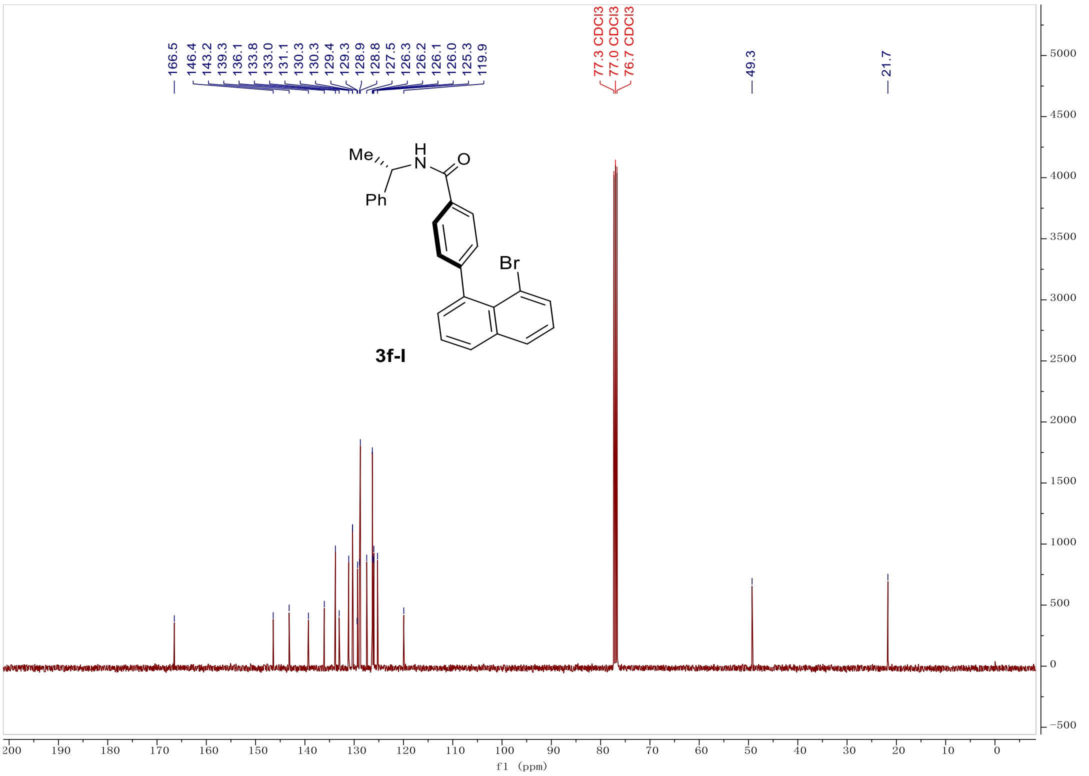
166.5
146.4
143.2
139.3
136.1
133.8
133.0
131.1
130.3
130.3
129.4
129.3
128.9
128.8
127.5
126.3
126.2
126.1
126.0
125.3
119.9
77.3 CDCl3
77.0 CDCl3
76.7 CDCl3
49.3
21.7
Me
H
N
O
Ph
Br
3f-I
f1 (ppm)

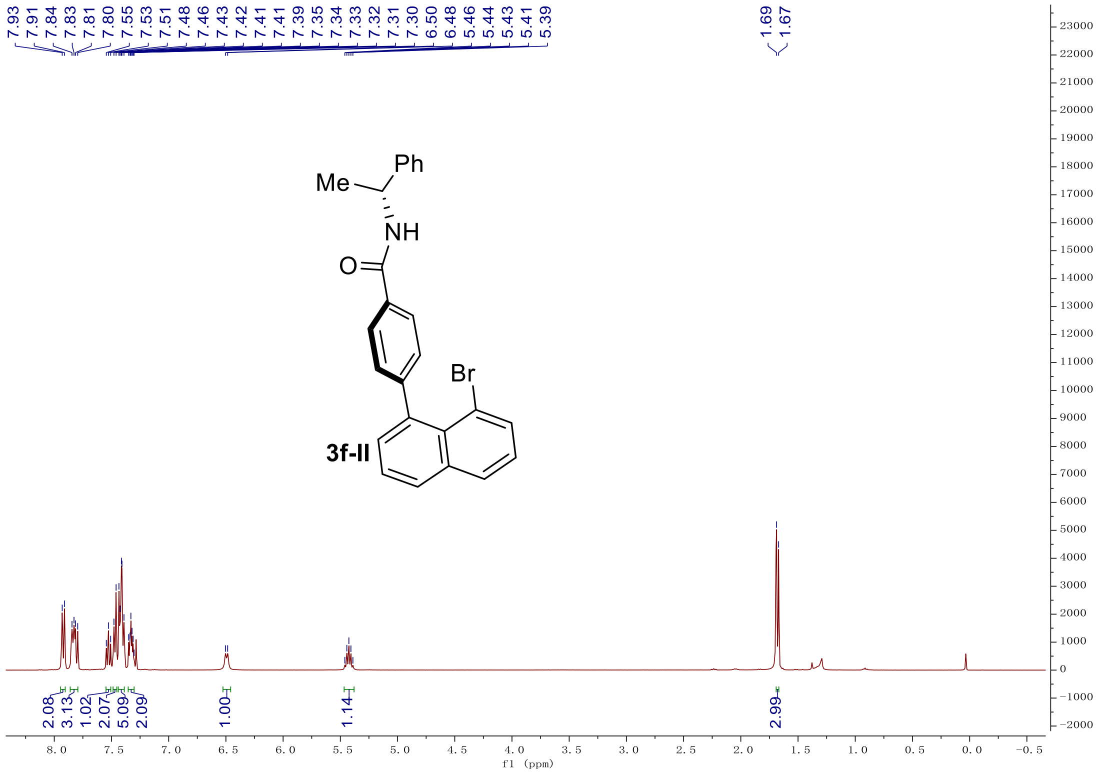
7.93 7.91 7.84 7.83 7.81 7.80 7.55 7.53 7.51 7.48 7.46 7.43 7.42 7.41 7.41 7.39 7.35 7.34 7.33 7.32 7.31 7.30 6.50 6.48 5.46 5.44 5.43 5.41 5.39
1.69 1.67
Me
Ph
NH
O
Br
3f-II
2.08 3.13 1.02 2.07 5.09 2.09
1.00
1.14
2.99
f1 (ppm)

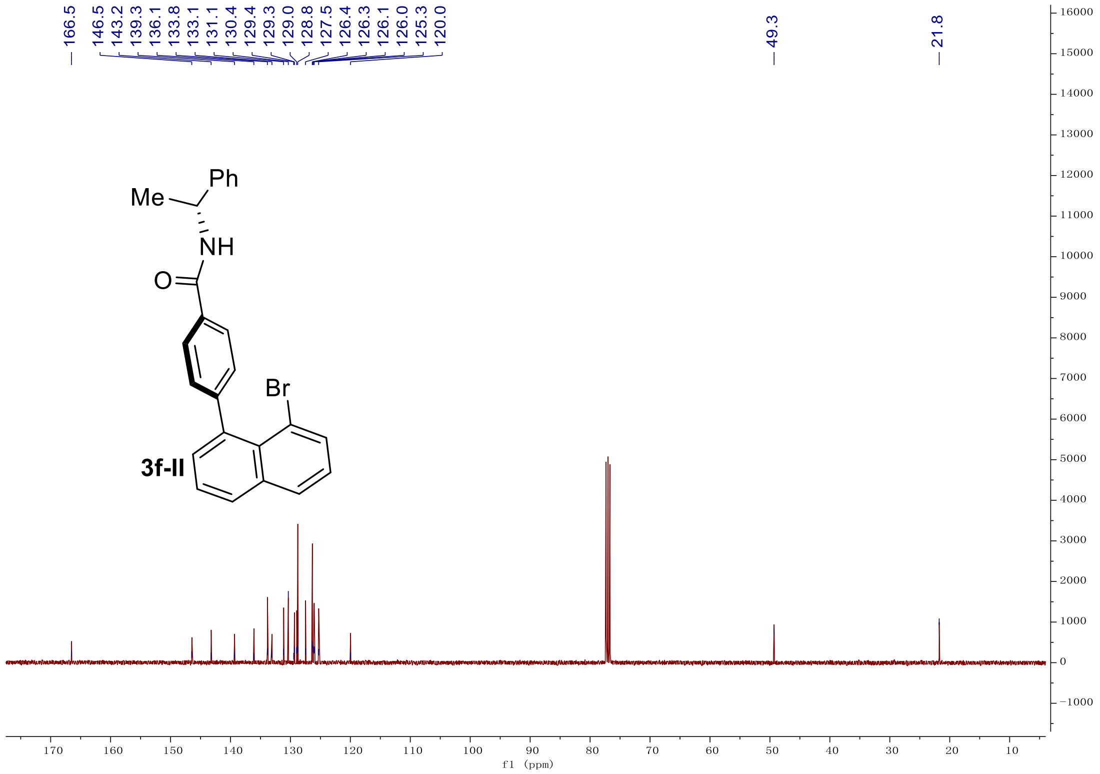
166.5
146.5 143.2 139.3 136.1 133.8 133.1 131.1 130.4 129.4 129.3 129.0 128.8 127.5 126.4 126.3 126.1 126.0 125.3 120.0
49.3
21.8
Me
Ph
NH
O
Br
3f-II
f1 (ppm)

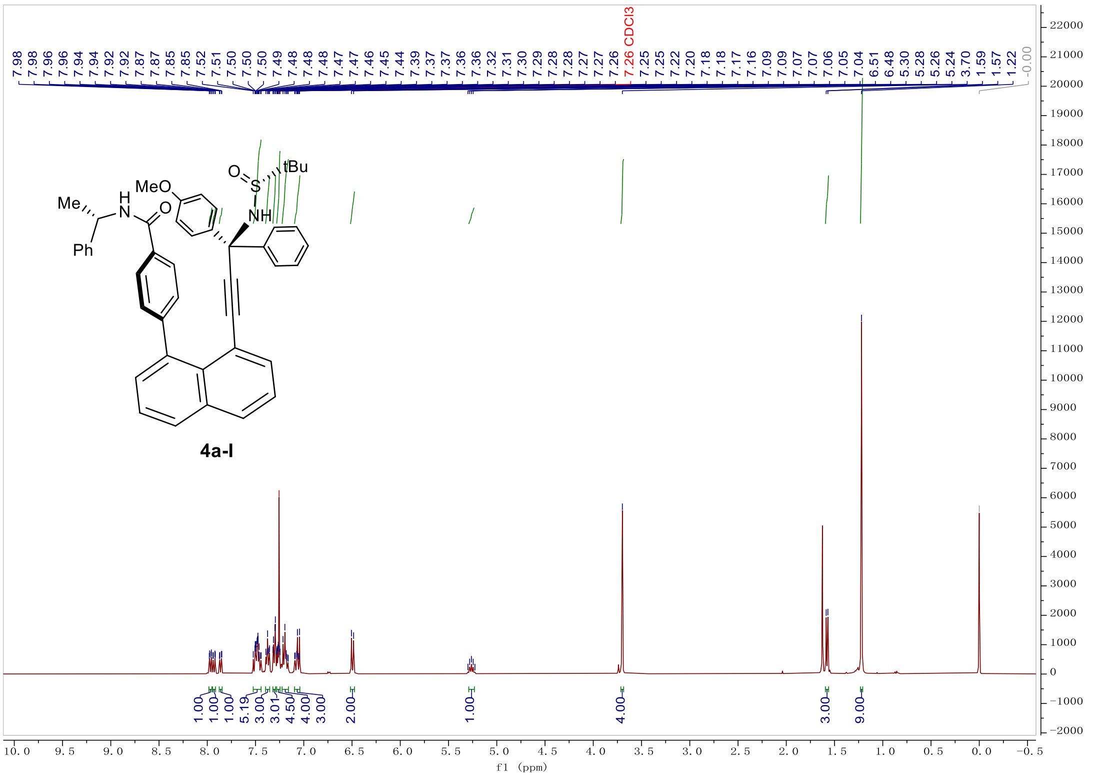
7.98 7.98 7.96 7.96 7.94 7.94 7.92 7.92 7.87 7.85 7.52 7.51 7.50 7.50 7.50 7.49 7.48 7.48 7.47 7.47 7.46 7.45 7.44 7.39 7.37 7.37 7.36 7.36 7.32 7.31 7.30 7.29 7.28 7.28 7.27 7.27 7.26
7.26 CDCl3
7.25 7.25 7.22 7.20 7.18 7.17 7.16 7.09 7.09 7.07 7.07 7.06 7.05 7.04 6.51 6.48 5.30 5.28 5.26 5.24 3.70 1.59 1.57 1.22
-0.00
MeO
O
S
tBu
NH
Me
H
N
O
Ph
4a-l
1.00 1.00 1.00 5.19 3.00 3.01 4.50 4.00 3.00 2.00 1.00 4.00 3.00 9.00
f1 (ppm)

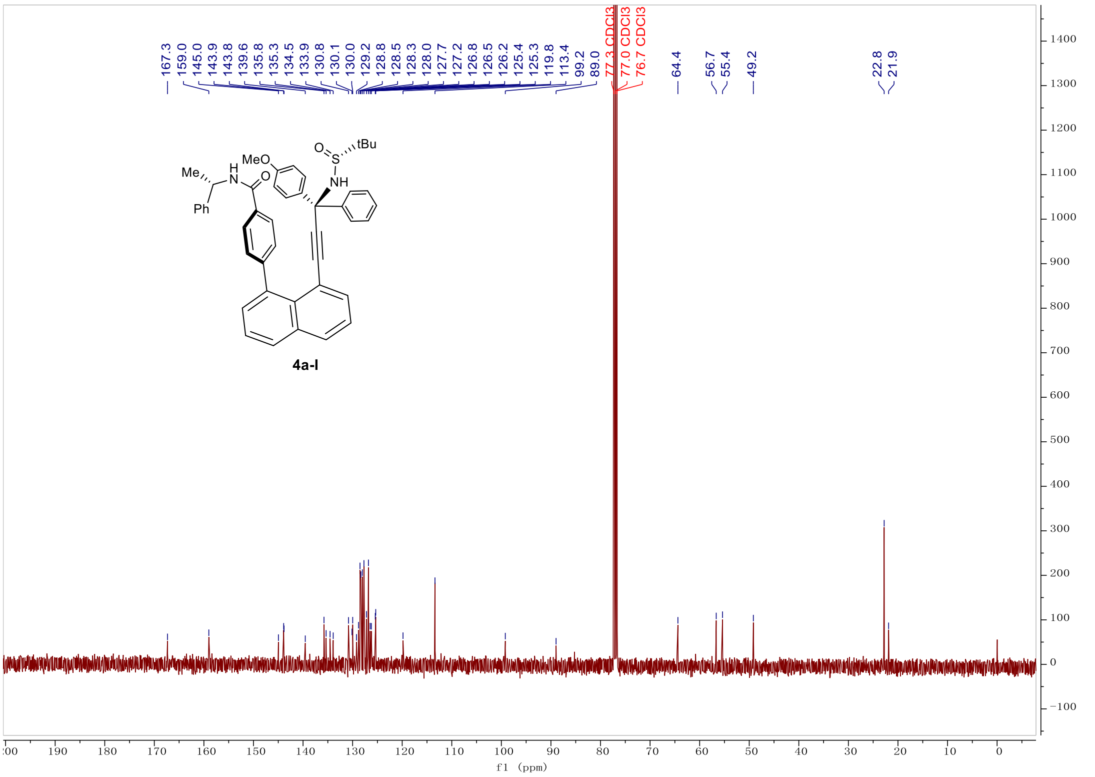
167.3 159.0 145.0 143.9 143.8 139.6 135.8 135.3 134.5 133.9 130.8 130.1 130.0 129.2 128.8 128.5 128.3 128.0 127.7 127.2 126.8 126.5 126.2 125.4 125.3 119.8 113.4 99.2 89.0
77.3 CDCl3
77.0 CDCl3
76.7 CDCl3
64.4 56.7 55.4 49.2 22.8 21.9
MeO
O
S
tBu
NH
Me
H
N
O
Ph
4a-l
f1 (ppm)

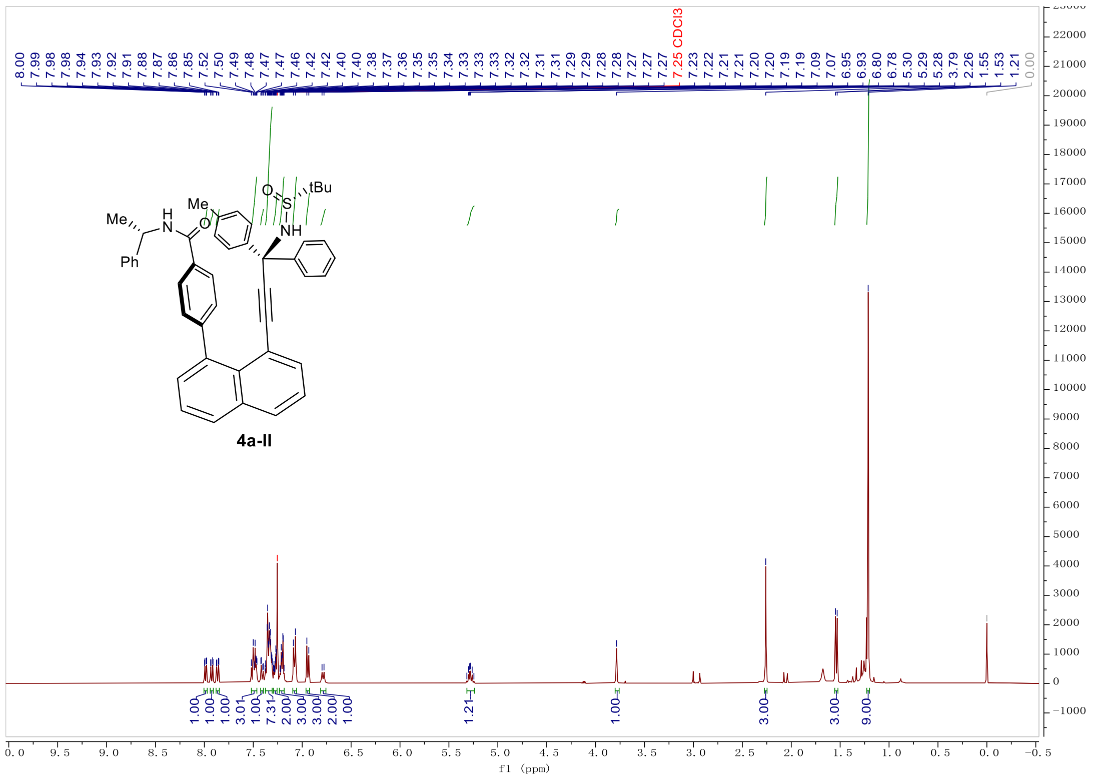

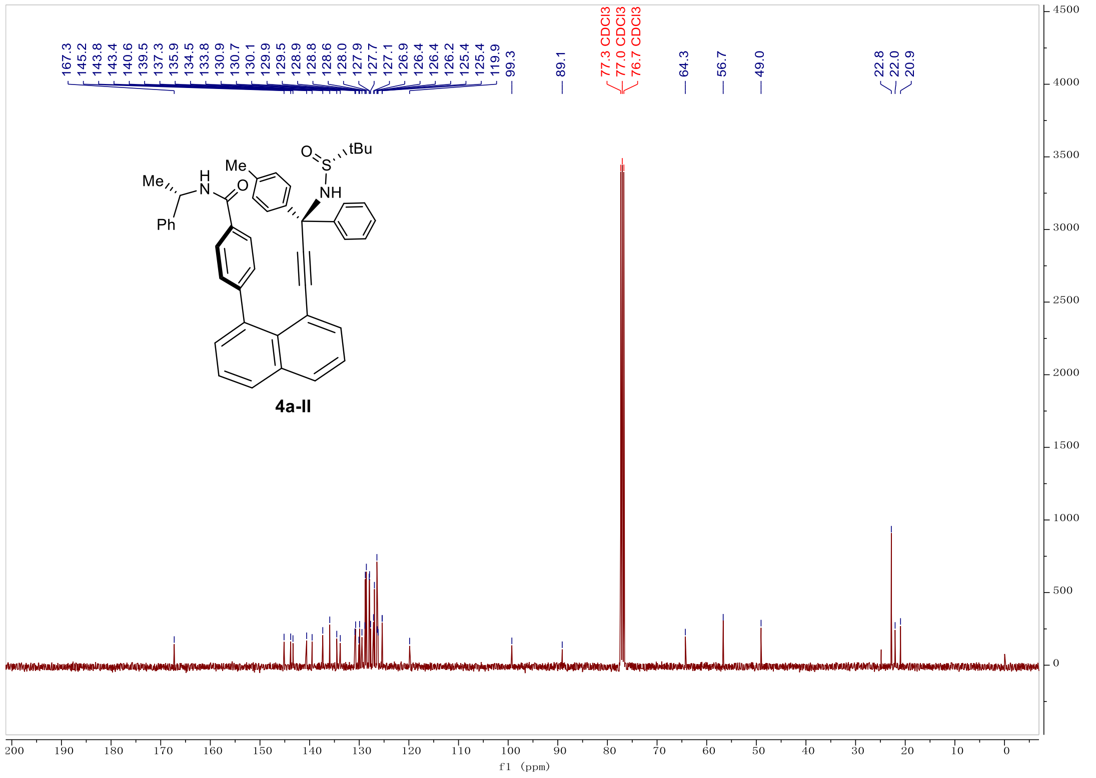

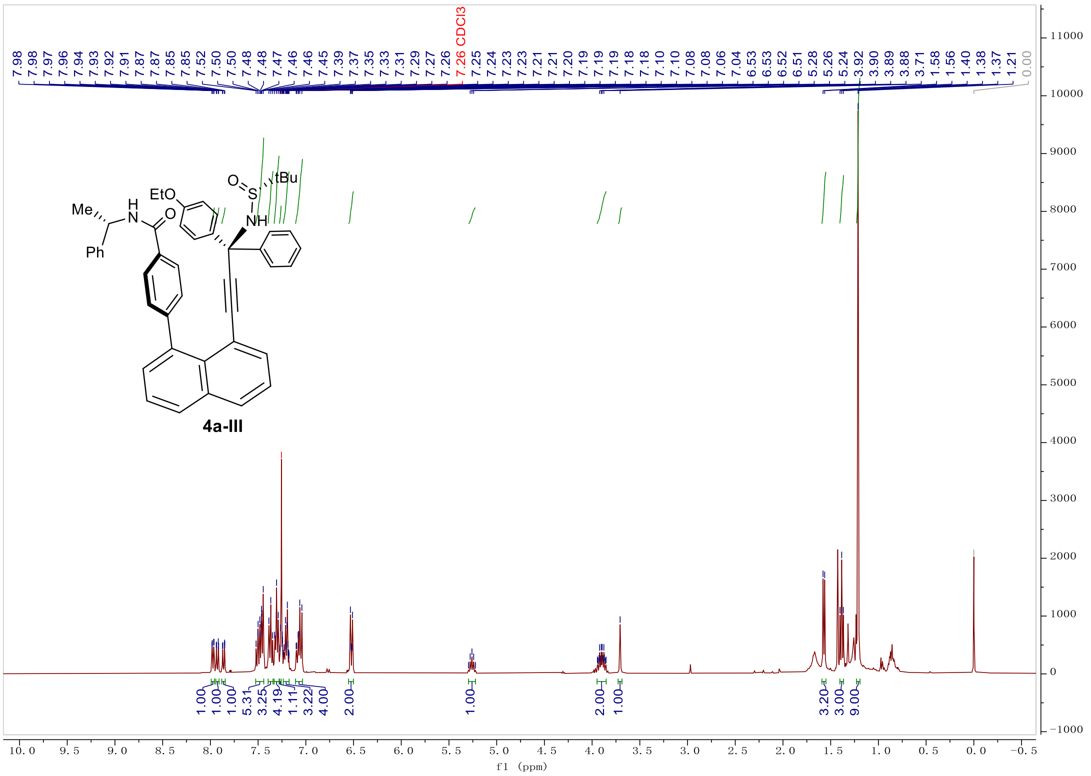
7.98 7.98 7.97 7.96 7.94 7.93 7.92 7.91 7.87 7.87 7.85 7.52 7.50 7.50 7.48 7.48 7.47 7.46 7.46 7.45 7.39 7.37 7.35 7.33 7.31 7.29 7.27 7.26 CDCl3 7.25 7.24 7.23 7.23 7.21 7.21 7.20 7.19 7.19 7.19 7.18 7.18 7.10 7.10 7.08 7.08 7.06 7.04 6.53 6.53 6.52 6.51 5.28 5.26 5.24 3.92 3.90 3.89 3.88 3.71 1.58 1.56 1.40 1.38 1.37 1.21 0.00
Me
H
N
O
Ph
EtO
O
S
tBu
NH
4a-III
1.00 1.00 1.00 5.31 3.25 4.19 1.11 3.22 4.00 2.00 1.00 2.00 1.00 3.20 3.00 9.00
f1 (ppm)

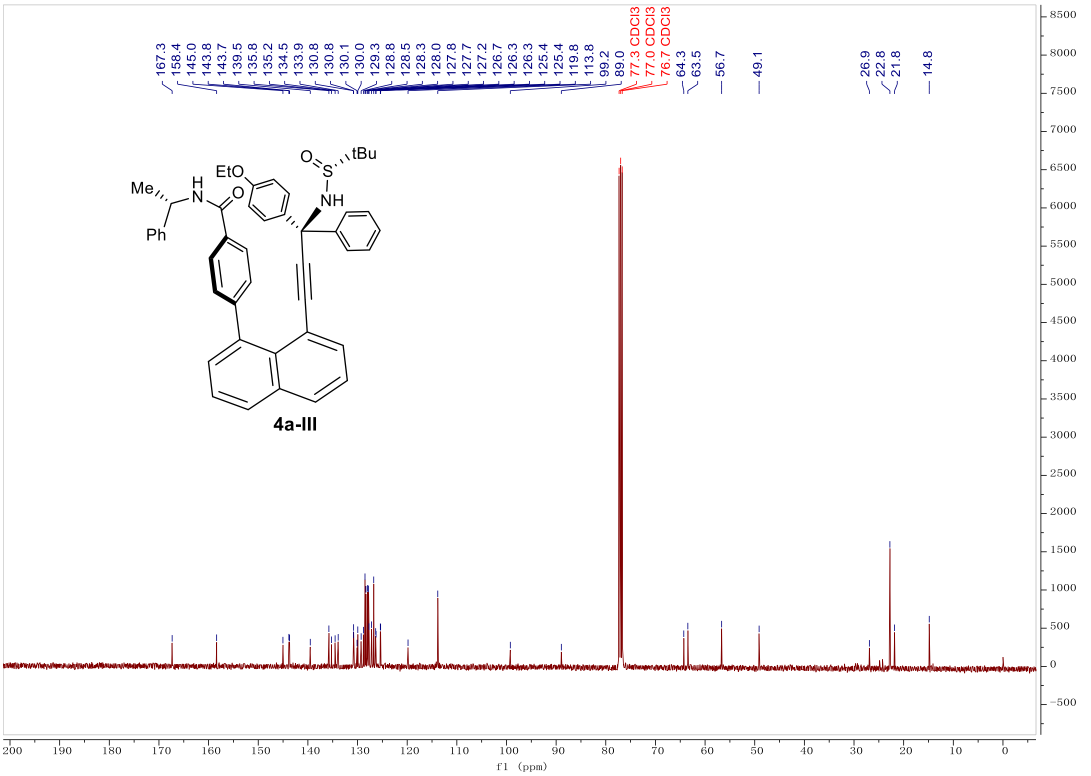
167.3 158.4 145.0 143.8 143.7 139.5 135.8 135.2 134.5 133.9 130.8 130.8 130.1 130.0 129.3 128.8 128.5 128.3 128.0 127.8 127.7 127.2 126.7 126.3 126.3 125.4 119.8 113.8 99.2 89.0 77.3 CDCl3 77.0 CDCl3 76.7 CDCl3 64.3 63.5 56.7 49.1 26.9 22.8 21.8 14.8
Me
H
N
O
Ph
EtO
O
S
tBu
NH
4a-III
f1 (ppm)

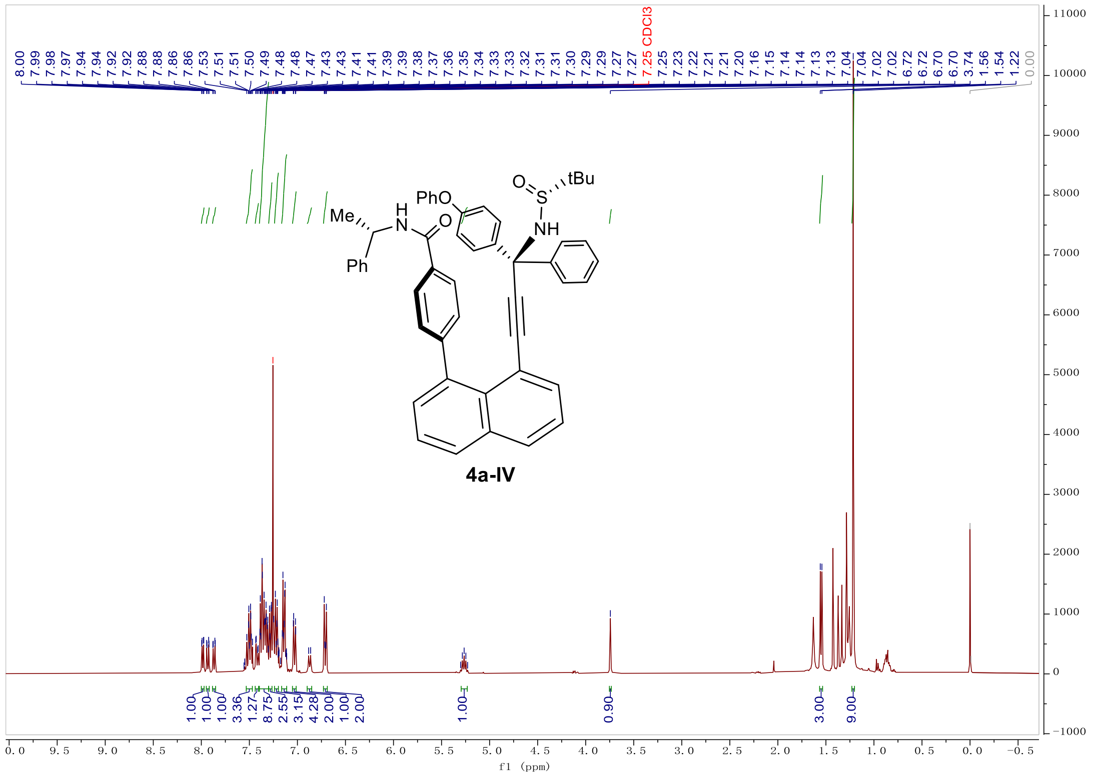
8.00
7.99
7.98
7.97
7.94
7.94
7.92
7.92
7.88
7.86
7.86
7.53
7.51
7.51
7.50
7.49
7.48
7.47
7.43
7.43
7.41
7.39
7.39
7.38
7.37
7.36
7.35
7.34
7.33
7.33
7.32
7.31
7.30
7.29
7.29
7.27
7.27
7.25 CDCl3
7.25
7.23
7.22
7.21
7.21
7.20
7.16
7.15
7.14
7.14
7.13
7.13
7.04
7.04
7.02
7.02
6.72
6.72
6.70
6.70
3.74
1.56
1.54
1.22
0.00
PhO
O
S
tBu
NH
Me
H
N
O
Ph
4a-IV
1.00
1.00
1.00
3.36
1.27
8.75
2.55
3.15
4.28
2.00
1.00
2.00
1.00
0.90
3.00
9.00
f1 (ppm)

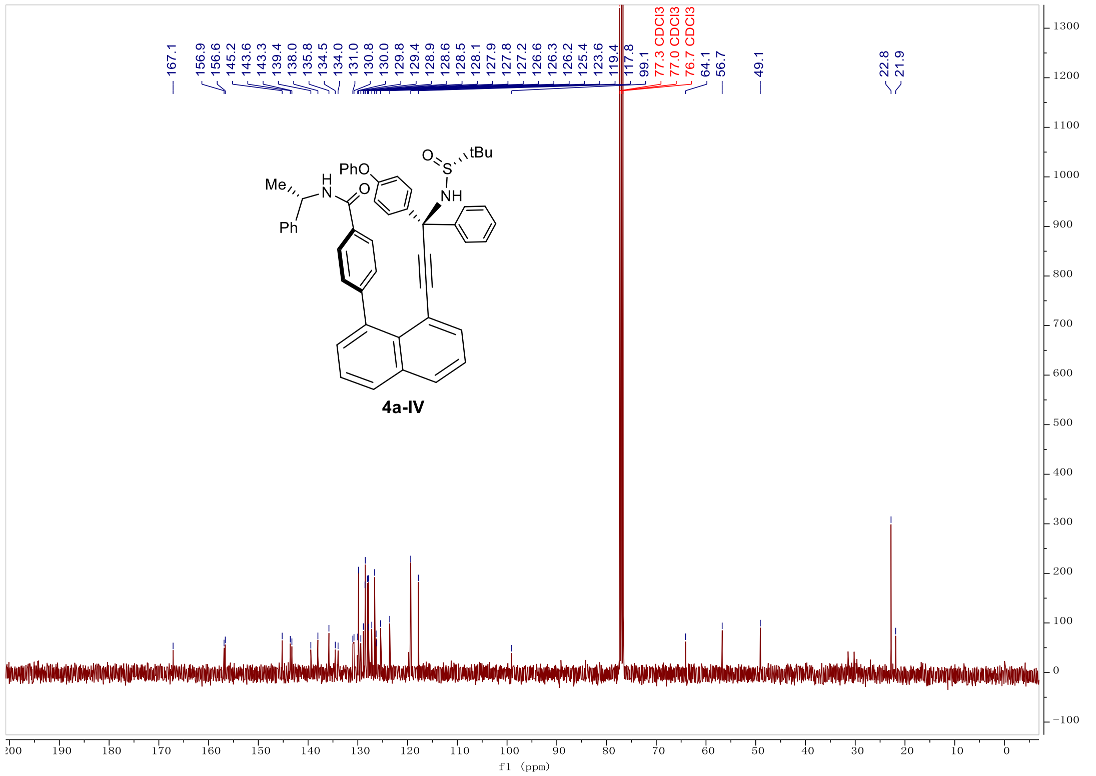
167.1
156.9
156.6
145.2
143.6
143.3
139.4
138.0
135.8
134.5
134.0
131.0
130.8
130.0
129.8
129.4
128.9
128.6
128.5
128.1
127.9
127.8
127.2
126.6
126.3
126.2
125.4
123.6
119.4
117.8
99.1
77.3 CDCl3
77.0 CDCl3
76.7 CDCl3
64.1
56.7
49.1
22.8
21.9
PhO
O
S
tBu
NH
Me
H
N
O
Ph
4a-IV
f1 (ppm)

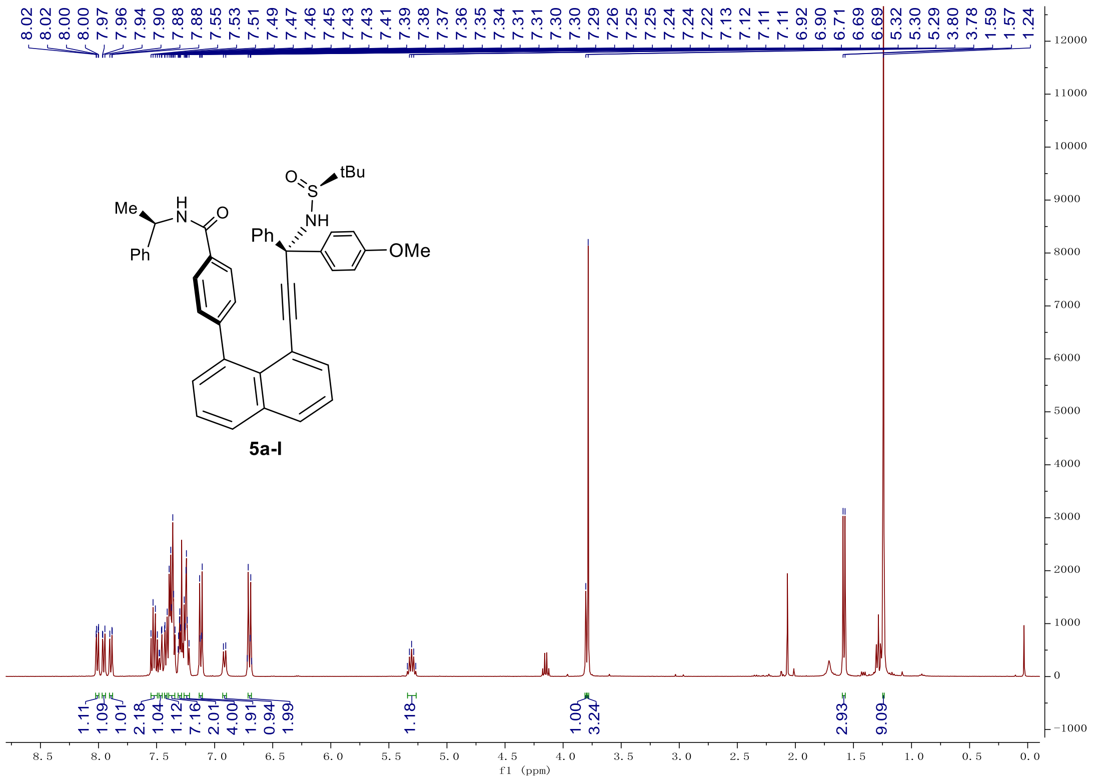
8.02 8.02 8.00 7.97 7.96 7.94 7.90 7.88 7.55 7.53 7.51 7.49 7.47 7.46 7.45 7.43 7.41 7.39 7.38 7.37 7.36 7.35 7.34 7.31 7.31 7.30 7.30 7.29 7.26 7.25 7.25 7.24 7.24 7.22 7.13 7.12 7.11 7.11 6.92 6.90 6.71 6.69 5.32 5.30 5.29 3.80 3.78 1.59 1.57 1.24
Me
H
N
O
Ph
O
S
tBu
NH
Ph
OMe
5a-l
1.11 1.09 1.01 2.18 1.04 1.12 7.16 2.01 4.00 1.91 0.94 1.99 1.18 1.00 3.24 2.93 9.09
f1 (ppm)

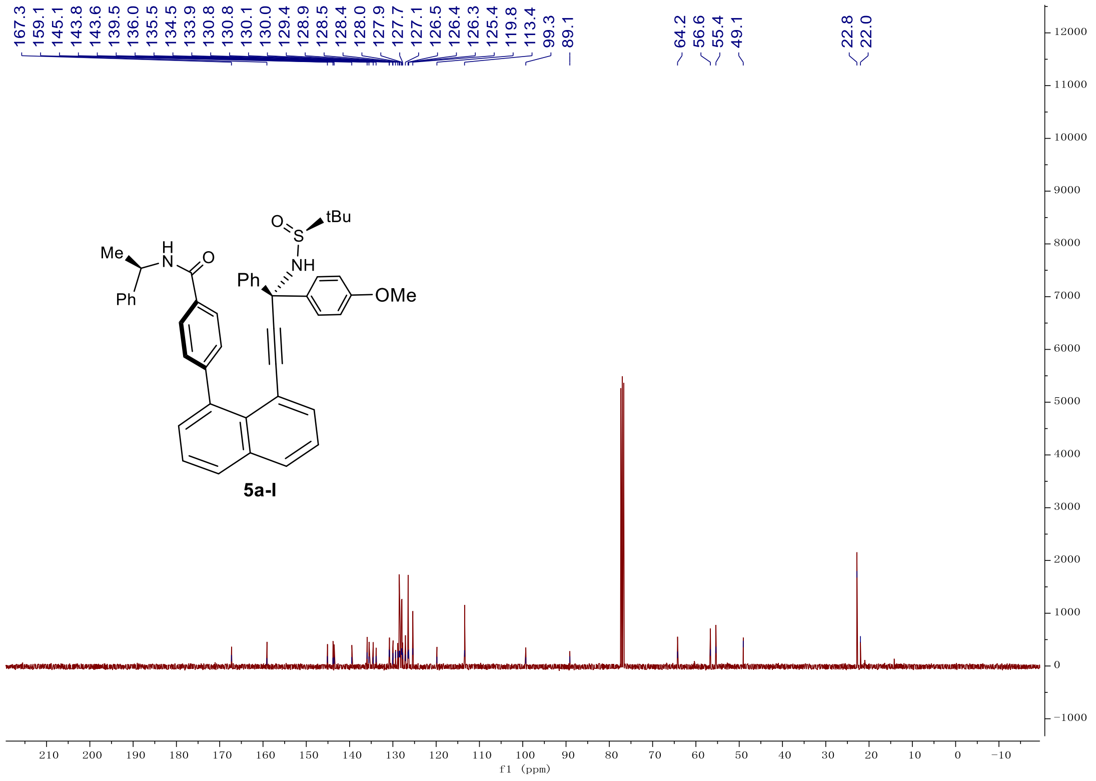
167.3 159.1 145.1 143.8 143.6 139.5 136.0 135.5 134.5 133.9 130.8 130.1 130.0 129.4 128.9 128.5 128.4 128.0 127.9 127.7 127.1 126.5 126.4 126.3 125.4 119.8 113.4 99.3 89.1 64.2 56.6 55.4 49.1 22.8 22.0
Me
H
N
O
Ph
O
S
tBu
NH
Ph
OMe
5a-l
f1 (ppm)

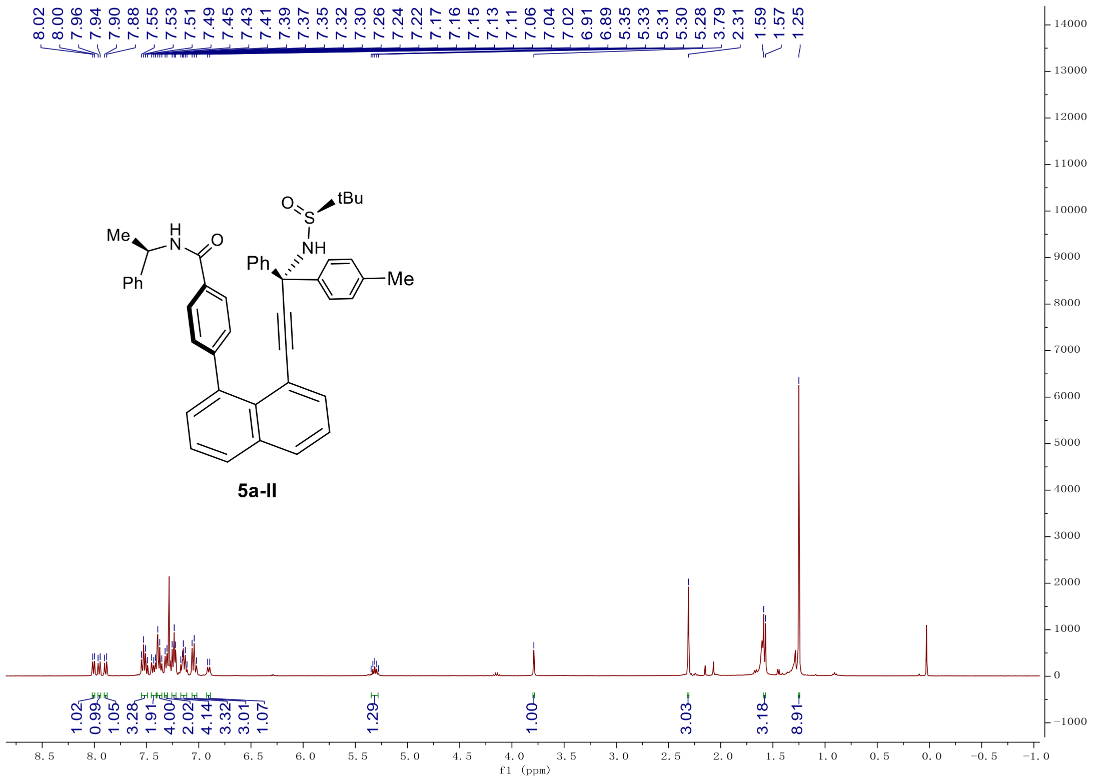
8.02
8.00
7.96
7.94
7.90
7.88
7.55
7.53
7.51
7.49
7.45
7.43
7.41
7.39
7.37
7.35
7.32
7.30
7.26
7.24
7.22
7.17
7.16
7.15
7.13
7.11
7.06
7.04
7.02
6.91
6.89
5.35
5.33
5.31
5.30
5.28
3.79
2.31
1.59
1.57
1.25
Me
H
N
O
O
S
tBu
NH
Ph
Me
Ph
5a-II
1.02
0.99
1.05
3.28
1.91
4.00
2.02
4.14
3.32
3.01
1.07
1.29
1.00
3.03
3.18
8.91
f1 (ppm)

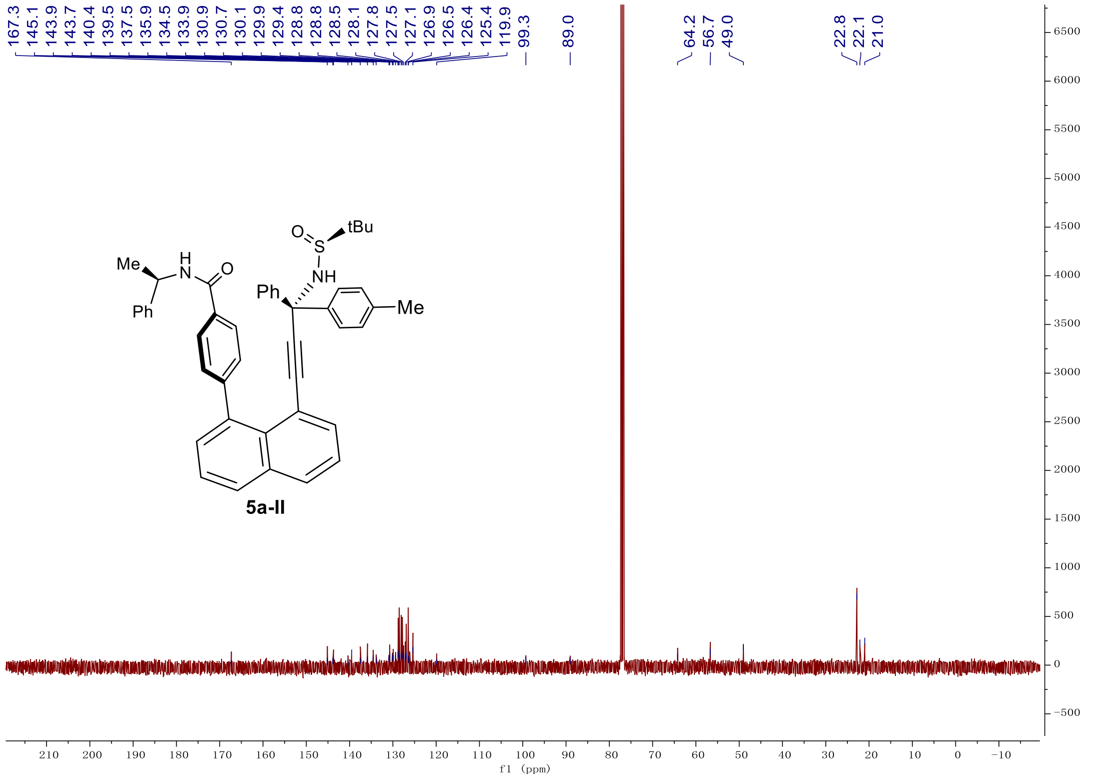
167.3
145.1
143.9
143.7
140.4
139.5
137.5
135.9
134.5
133.9
130.9
130.7
130.1
129.9
129.4
128.8
128.8
128.5
128.1
127.8
127.5
127.1
126.9
126.5
126.4
125.4
119.9
99.3
89.0
64.2
56.7
49.0
22.8
22.1
21.0
Me
H
N
O
O
S
tBu
NH
Ph
Me
Ph
5a-II
f1 (ppm)

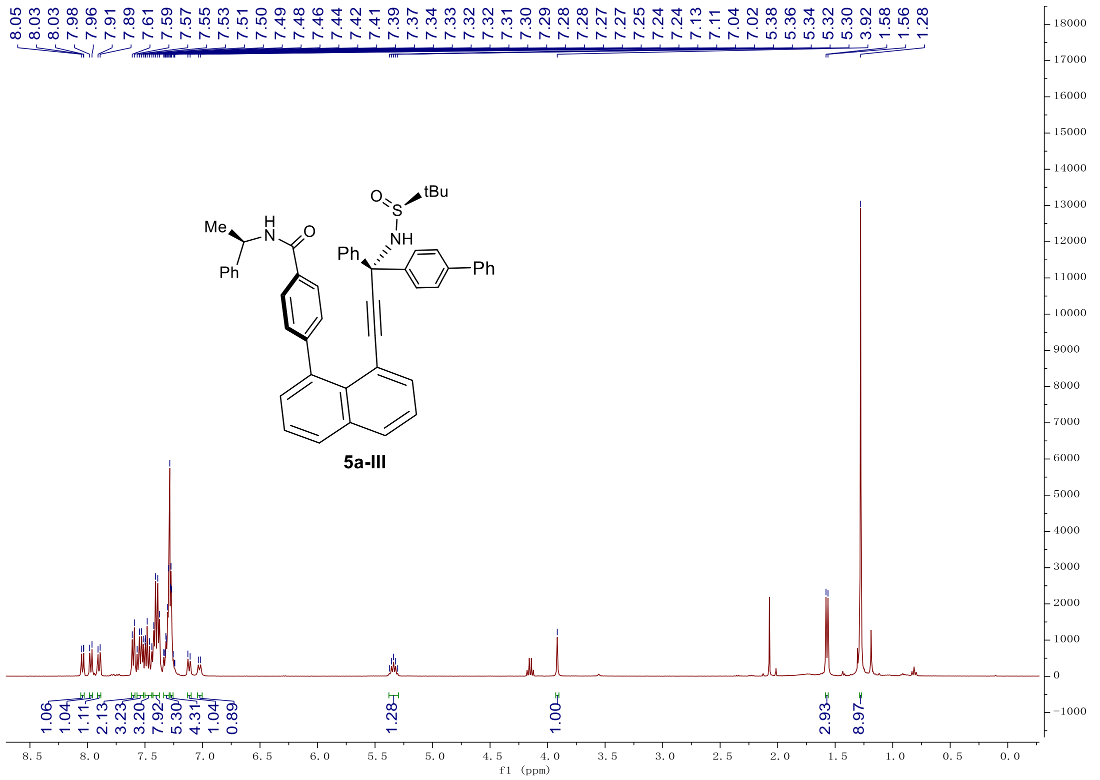
8.05 8.03 8.03 7.98 7.96 7.91 7.89 7.61 7.59 7.57 7.55 7.53 7.51 7.50 7.49 7.48 7.46 7.44 7.42 7.41 7.39 7.37 7.34 7.33 7.32 7.32 7.31 7.30 7.29 7.28 7.28 7.27 7.27 7.25 7.24 7.24 7.13 7.11 7.04 7.02 5.38 5.36 5.34 5.32 5.30 3.92 1.58 1.56 1.28
Me
H
N
O
Ph
O
S
tBu
NH
Ph
Ph
5a-III
1.06 1.04 1.11 2.13 3.23 3.20 7.92 5.30 4.31 1.04 0.89
1.28
1.00
2.93
8.97
f1 (ppm)

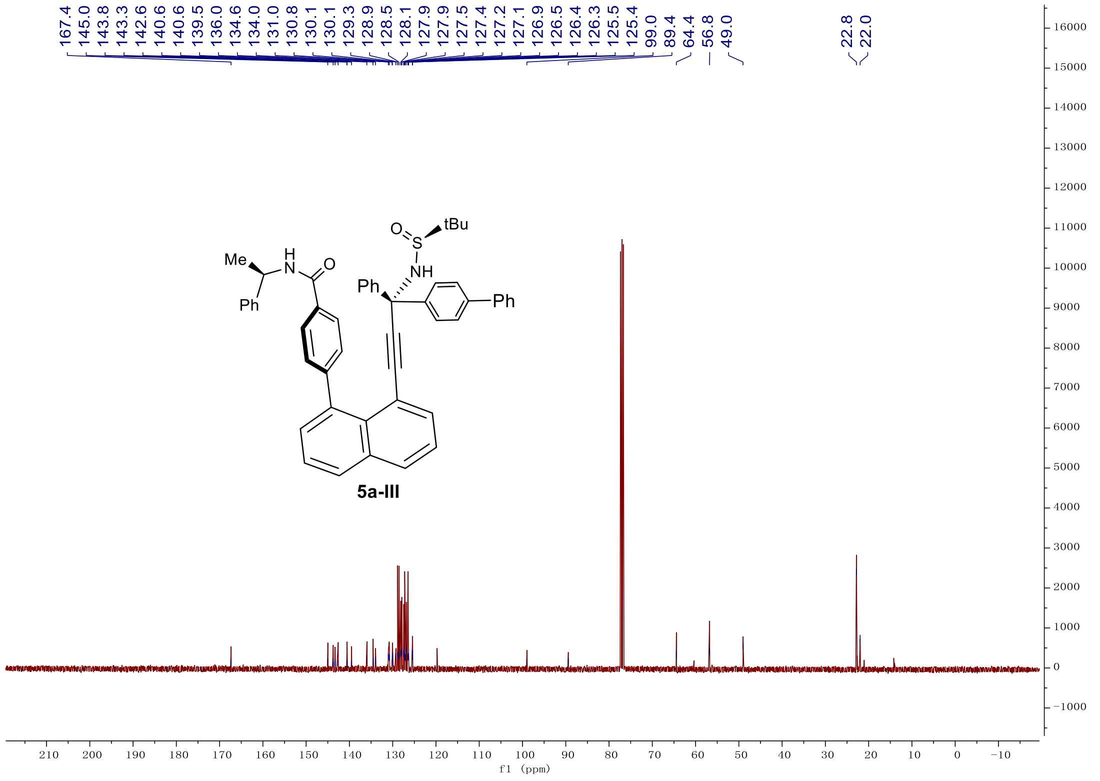
167.4 145.0 143.8 143.3 142.6 140.6 140.6 139.5 136.0 134.6 134.0 131.0 130.8 130.1 130.1 129.3 128.9 128.5 128.1 127.9 127.5 127.4 127.2 127.1 126.9 126.5 126.4 126.3 125.5 125.4 99.0 89.4 64.4 56.8 49.0
22.8 22.0
Me
H
N
O
Ph
O
S
tBu
NH
Ph
Ph
5a-III
f1 (ppm)

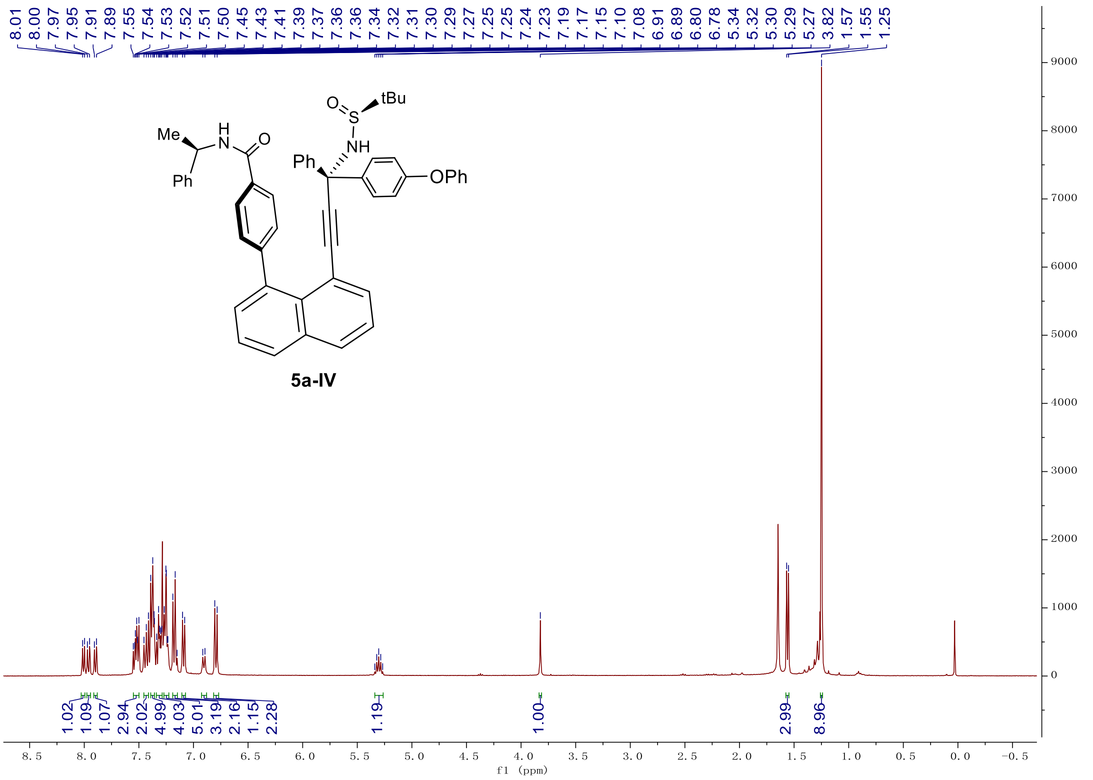
8.01 8.00 7.97 7.95 7.91 7.89 7.55 7.54 7.53 7.52 7.51 7.50 7.45 7.43 7.41 7.39 7.37 7.36 7.36 7.34 7.32 7.31 7.30 7.29 7.27 7.25 7.25 7.24 7.23 7.19 7.17 7.15 7.10 7.08 6.91 6.89 6.80 6.78 5.34 5.32 5.30 5.29 5.27 3.82 1.57 1.55 1.25
1.02 1.09 1.07 2.94 2.02 4.99 4.03 5.01 3.19 2.16 1.15 2.28 1.19 1.00 2.99 8.96
Me
H
N
O
Ph
O
S
tBu
NH
Ph
OPh
5a-IV
f1 (ppm)

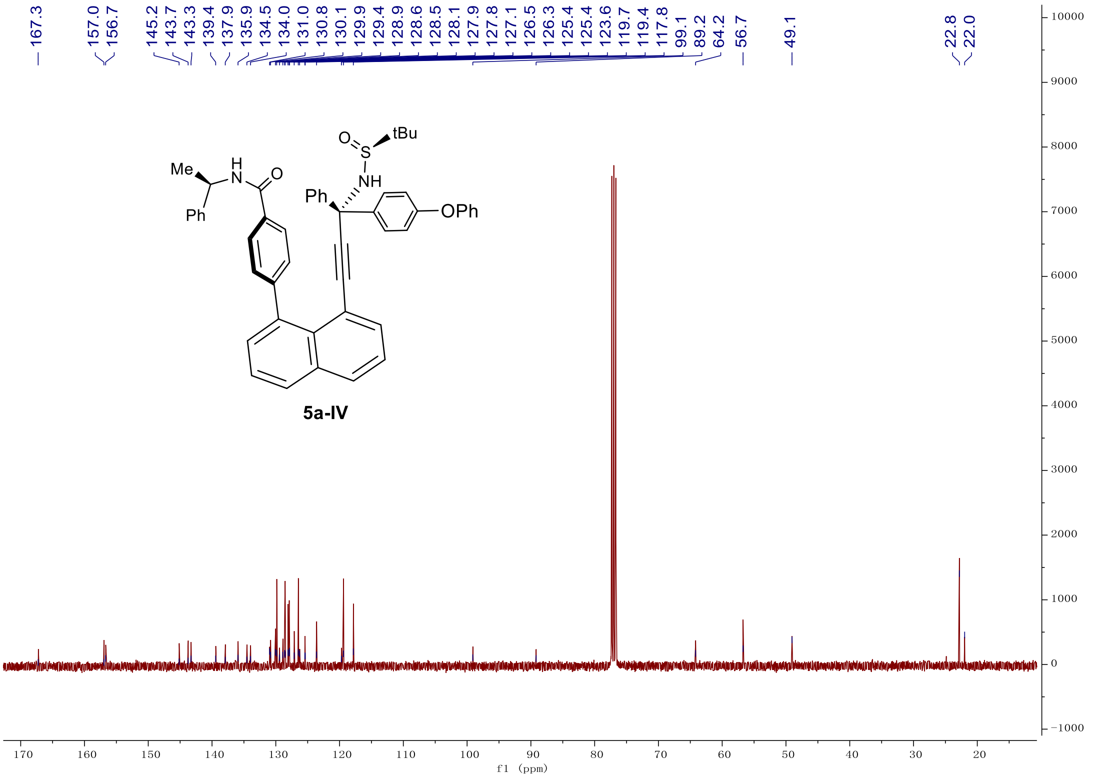
167.3 157.0 156.7 145.2 143.7 143.3 139.4 137.9 135.9 134.5 134.0 131.0 130.8 130.1 129.9 129.4 128.9 128.6 128.5 128.1 127.9 127.8 127.1 126.5 126.3 125.4 123.6 119.7 119.4 117.8 99.1 89.2 64.2 56.7 49.1 22.8 22.0
Me
H
N
O
Ph
O
S
tBu
NH
Ph
OPh
5a-IV
f1 (ppm)

4. X-ray Data & calculation

Figure S1

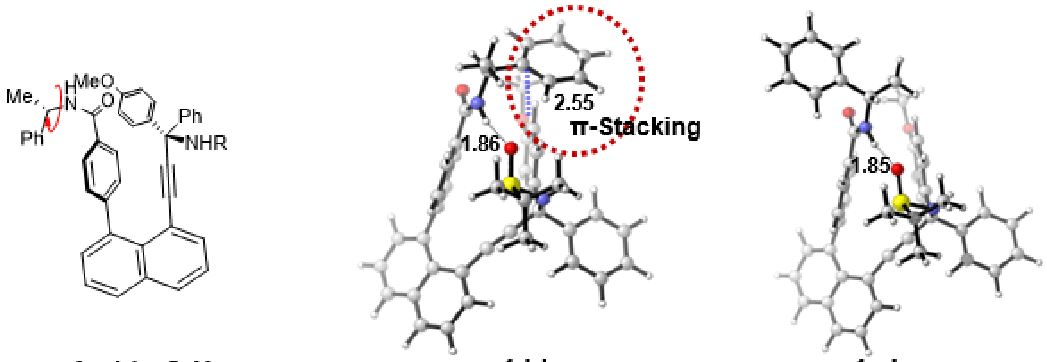

Amide *C-N* bond rotation | **4d-I** ΔG = 0.0 | **4e-I** ΔG = + 3.7

Figure S2

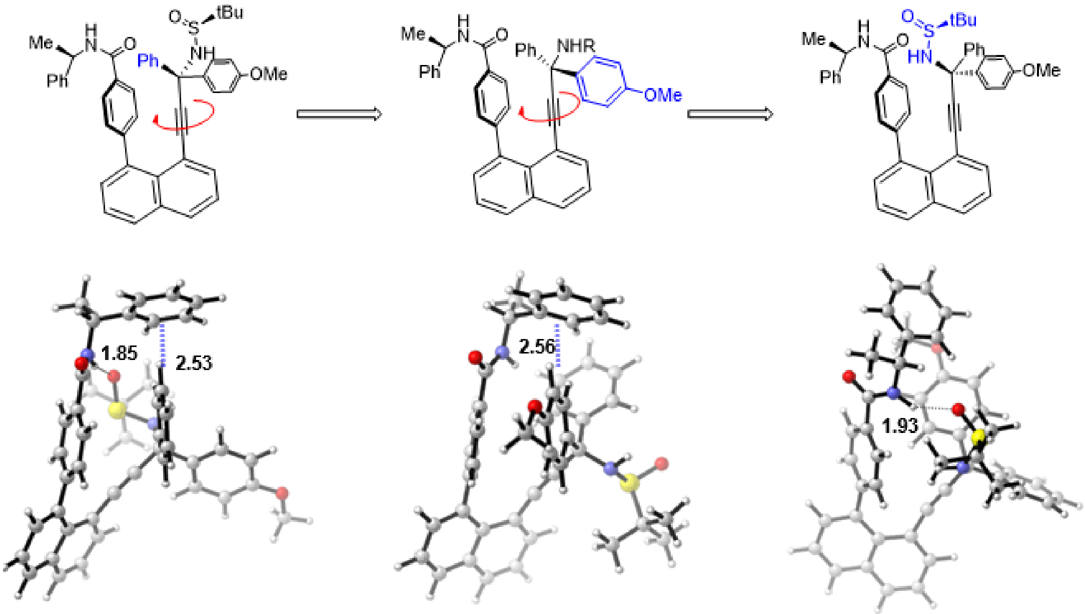

5a-I ΔG = 0.0 | **5b-I** ΔG = + 2.5 | **5c-I** ΔG = + 7.4

Figure S3

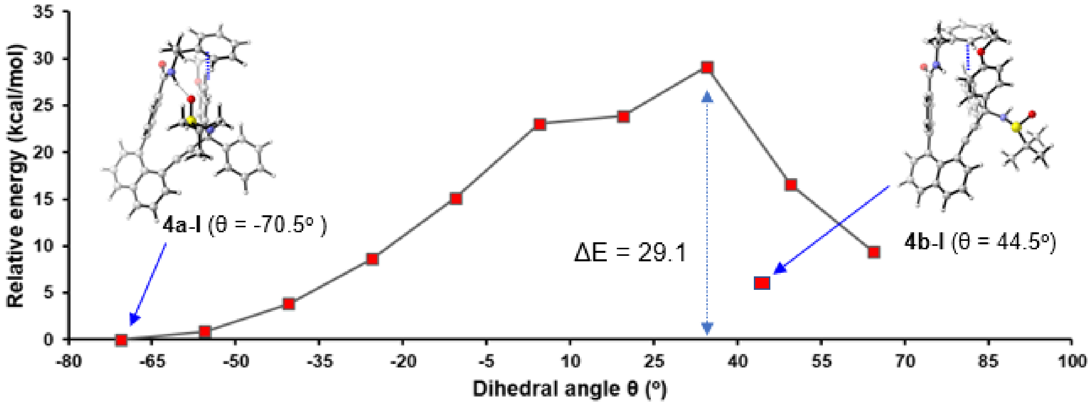

Relative energy (kcal/mol)
35
30
25
20
15
10
5
0
-80
-65
-50
-35
-20
-5
10
25
40
55
70
85
100
Dihedral angle θ (º)
4a-I (θ = -70.5º)
4b-I (θ = 44.5º)
ΔE = 29.1
